# A tumour-derived organoid biobank maps cancer gene dependencies

**DOI:** 10.1038/s41586-026-10830-y

**Published:** 2026-08-05

**Authors:** C. Herranz-Ors, S. G. Bhosle, A. E. Beck, J. G. R. Gilbert, G. Picco, J. Espejo Valle-Inclan, F. Muyas, S. Valentini, A. E. Andres, R. Ansari, S. Barthorpe, G. Battarbee, C. M. Beaver, S. Brocklesby, J. Cantwell, C. A. Collins, J. Davis, H. G. Dimitrova, J. Doran, E. Efendi, K. Evans, M. Fekry, T. A. Fowler, M. Garcia-Casado, J. A. T. Griffiths, C. Hall, R. Hamer, C. Hardy, Z. Hewitson, E. Hitch, L. Holland, D. A. Jackson, N. Joshi, A. Kavasakali, L. Letchford, H. B. Lightfoot, H. Lingala, I. Mali, K. May, T. Mironenko, J. Morris, C. Pacini, S. Price, G. Robert-Tissot, H. A. Rogers, J. V. Smith, K. Smith, E. Souster, W. J. Spence, F. Thomas, S. F. Vieira, S. Walker, G. Alfonsin, H. Bermingham, H. Coles, D. P. Ennis, A. Freeman, G. Giannone, N. Grehan, E. A. Griffiths, J. Hall, S. L. Lee, E. Y. L. Leung, C. Loreno, C. Millington, A. Mirnezami, B. Nutzinger, K. Orzechowska, C. M. A. Pinna, A. M. Redmond, K. Roberts, S. Roy, D. A. Sanders, P. Taniere, M. Vias, K. Wanigasooriya, OCCAMS Consortium, H. Coles, A. Freeman, N. Grehan, B. Nutzinger, A. M. Redmond, P. Taniere, P. A. W. Edwards, S. Abbas, E. C. Smyth, M. O’Donovan, A. Miremadi, S. Malhotra, M. Tripathi, C. Cheah, M. Eldridge, M. Secrier, G. Devonshire, S. Jammula, J. Davies, C. Crichton, N. Carroll, R. H. Hardwick, P. Safranek, A. Hindmarsh, V. Sujendran, S. J. Hayes, Y. Ang, A. Sharrocks, S. R. Preston, I. Bagwan, V. Save, R. J. E. Skipworth, J. R. O’Neill, O. Tucker, A. Beggs, S. Puig, G. Contino, B. L. Grace, J. Lagergren, J. Gossage, A. Davies, F. Chang, U. Mahadeva, V. Goh, F. D. Ciccarelli, G. Sanders, D. Chan, E. Cheong, B. Kumar, L. Sreedharan, S. L. Parsons, I. Soomro, P. Kaye, J. Saunders, L. Lovat, R. Haidry, M. Scott, S. Sothi, G. B. Hanna, C. J. Peters, K. Moorthy, A. Grabowska, R. Turkington, D. McManus, H. Coleman, R. D. Petty, F. Bartlett, T. D. L. Crosby, T. J. Underwood, R. C. Fitzgerald, M. R. Stratton, L. M. Staudt, U. McDermott, J. D. Brenton, I. A. McNeish, A. Biankin, O. J. Sansom, I. Cortes-Ciriano, T. J. Underwood, R. C. Fitzgerald, A. D. Beggs, H. E. Francies, M. J. Garnett

**Affiliations:** 1 Wellcome Sanger Institute, Cambridge, UK; 2 European Molecular Biology Laboratory, European Bioinformatics Institute, Cambridge, UK; 3 Cancer Research UK Cambridge Institute, University of Cambridge, Li Ka Shing Centre, Cambridge, UK; 4 AstraZeneca, The Discovery Centre (DISC), Cambridge, UK; 5 Instituto de Investigación Biomédica A Coruña (INIBIC), A Coruña, Spain; 6 University Hospitals Birmingham NHS Foundation Trust, Birmingham, UK; 7 Early Cancer Institute, University of Cambridge, Cambridge, UK; 8 Department of Surgery and Cancer, Imperial College London, London, UK; 9 Institute of Immunology and Immunotherapy, College of Medical and Dental Science, University of Birmingham, Birmingham, UK; 10 School of Cancer Sciences, Faculty of Medicine, University of Southampton, Southampton, UK; 11 Department of Cancer and Genomic Sciences, College of Medicine and Health, University of Birmingham, Birmingham, UK; 12 University Hospital Southampton NHS Foundation Trust, Southampton, UK; 13 George Eliot Hospital NHS Trust, Nuneaton, UK; 14 Lymphoid Malignancies Branch, Center for Cancer Research, National Cancer Institute, Bethesda, MD USA; 15 School of Cancer Sciences, University of Glasgow, Glasgow, UK; 16 Cancer Research UK Scotland Institute, Glasgow, UK; 17 Cambridge University Hospitals NHS Foundation Trust, Cambridge, UK; 18 Department of Histopathology, Addenbrookes Hospital, Cambridge, UK; 19 Department of Computer Science, University of Oxford, Oxford, UK; 20 Salford Royal NHS Foundation Trust, Salford, UK; 21 Faculty of Medical and Human Sciences, University of Manchester, Manchester, UK; 22 Wigan and Leigh NHS Foundation Trust, Wigan, UK; 23 GI Science Centre, University of Manchester, Manchester, UK; 24 Royal Surrey County Hospital NHS Foundation Trust, Guildford, UK; 25 Edinburgh Royal Infirmary, Edinburgh, UK; 26 Edinburgh University, Edinburgh, UK; 27 Heart of England NHS Foundation Trust, Birmingham, UK; 28 Guy’s and St Thomas’s NHS Foundation Trust, London, UK; 29 Karolinska Institute, Stockholm, Sweden; 30 King’s College London, London, UK; 31 Plymouth Hospitals NHS Trust, Plymouth, UK; 32 Norfolk and Norwich University Hospital NHS Foundation Trust, Norwich, UK; 33 Nottingham University Hospitals NHS Trust, Nottingham, UK; 34 University College London, London, UK; 35 Wythenshawe Hospital, Manchester, UK; 36 University Hospitals Coventry and Warwickshire NHS Trust, Coventry, UK; 37 Queen’s Medical Centre, University of Nottingham, Nottingham, UK; 38 Centre for Cancer Research and Cell Biology, Queen’s University Belfast, Belfast, UK; 39 Tayside Cancer Centre, Ninewells Hospital and Medical School, Dundee, Scotland; 40 Portsmouth Hospitals NHS Trust, Portsmouth, UK; 41 Velindre University NHS Trust, Cardiff, UK; 42 Present Address: AstraZeneca, The Discovery Centre (DISC), Cambridge, UK; 43 Present Address: GlaxoSmithKline (GSK), Stevenage, UK

**Keywords:** Cancer models, Cancer genomics, High-throughput screening

## Abstract

Cancer cell lines remain foundational for research and drug discovery, yet they incompletely capture tumour diversity, lack linked patient context, and have undergone adaptation to culture. Tumour organoids are three-dimensional cultures derived from patient tissue that offer a powerful complement to cell lines^1^. Here we derived and characterized 256 clinically annotated tumour organoids directly from colorectal, oesophageal, ovarian, pancreatic and gastric cancers as renewable, genetically stable models. Extensive characterization of each model and matched patient tumour samples included whole-genome and transcriptome sequencing, and genome-wide CRISPR–Cas9 screens across 162 organoids mapped gene dependencies. Integrative analyses revealed genomic and clinical markers of dependency across common and rare subtypes, identified organoid-specific essential genes, and revealed targetable vulnerabilities following tumour evolution in paired pre- and post-treatment samples. In colorectal cancer, functional and pharmacological interrogation of the EGFR–RAS–MAPK axis uncovered differential effects of *KRAS* variant alleles. This open, publicly available resource provides a systematic map of gene dependencies in patient-derived organoids, expanding the model diversity and mechanistic insight needed to advance precision oncology.

## Abstract (web summary)

A clinically annotated multi-omic organoid resource with matched tumour samples from 256 patients across 5 cancers maps gene dependencies, reveals subtype-specific vulnerabilities and uncovers treatment-driven changes, expanding mechanistic insight and models for precision oncology.

## Main

The multi-omics characterization of hundreds of 2D cancer cell lines and their perturbation in pharmacological and genetic screens^2–9^ has been used to build reference maps of cancer dependencies to inform precision cancer medicines. However, the widely available set of around 1,000 human cancer cell lines^9^ has limitations, including biased representation of certain cancer subtypes or absence of others, no patient-matched germline genetic data for accurately calling somatic variation, and a lack of linked patient characteristics or clinical data. Furthermore, most cell lines adapt to in vitro culture in ways that are not well understood, and reference patient-matched tumour samples are unavailable for comparisons, collectively hampering their use for pre-clinical research and discovery. Patient-derived xenograft models capture elements of the tumour microenvironment, but require specialized facilities and substantial resources, limiting broader use.

Tumour organoids are 3D cultures derived from patient tissue grown with niche factors in an extracellular matrix. They can be derived with higher success rates, capture inter-tumour heterogeneity and better reflect tumour histological, genetic and functional features than cell lines (reviewed in ref. ^1^). However, scientific, technical and practical constraints have limited their widespread adoption^10^. Existing biobanks are relatively small (the two largest academic collections include 115 and 96 models), limiting representation of tumour diversity and so do not capture tumour heterogeneity^11,12^. Many lack comprehensive genomic annotation, with only limited or partial molecular characterization, restricting links to clinical data and systematic evaluation of their fidelity to parent tumours. Some organoids are non-renewable or short-term cultures not suitable for biobanking^13,14^, and others have restrictive ethical approvals that limit access. Finally, it is unclear whether a large, heterogeneous biobank of tumour organoids, given their greater complexity in culture compared to cell lines, can support systematic mapping of cancer vulnerabilities.

Here we report results from an extensive enterprise in the UK, in partnership with the Human Cancer Model Initiative (HCMI), to derive a highly annotated renewable tumour organoid biobank as an open community resource, and demonstrate its utility for mapping cancer dependencies to inform precision cancer medicine.

## Renewable patient organoid biobank

**Fig. 1:**
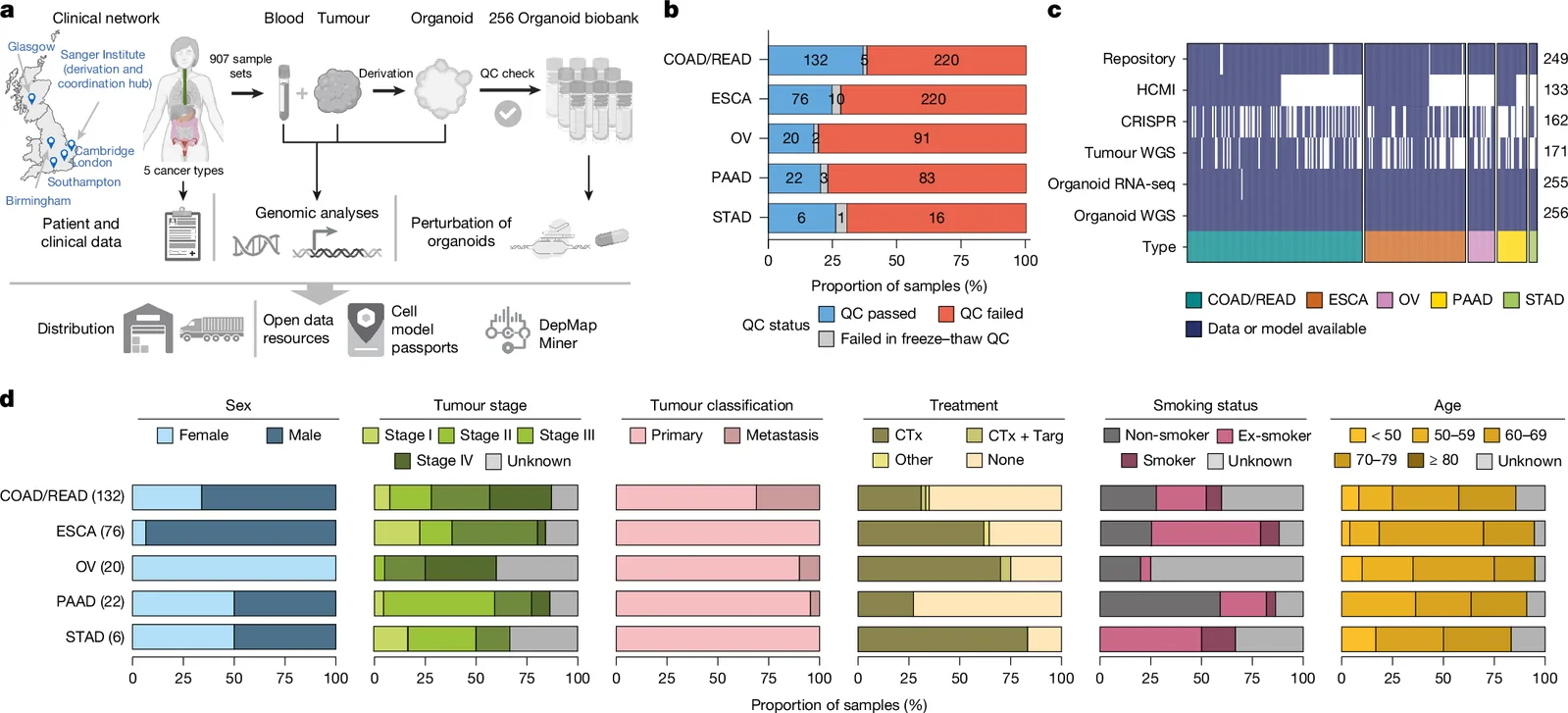
Establishment of organoid biobank and patient-linked clinical data. **a**, Overview of biobanking, genomic analysis and dependency mapping. Illustration created in BioRender; Herranz-Ors, C. https://BioRender.com/kovv6dd (2026). **b**, Derivation success rates per cancer type. **c**, Availability of WGS for organoids and matched patient tumours, RNA-seq and CRISPR screens in established organoids, organoids included as part of HCMI, and organoids currently deposited with repositories. **d**, Example clinical data from patients whose cancer samples were successfully established as organoids. Tumour stage is shown using the pathological tumour-node-metastasis (TNM) system for COAD/READ, ESCA, PAAD and STAD organoids, and the International Federation of Gynecology and Obstetrics (FIGO) system for OV organoids. For treatment type, ‘Other’ refers to hormonal therapy, targeted therapy alone or a combination of chemotherapy and immunotherapy; CTx, chemotherapy; QC, quality control; Targ, targeted therapy.

To create an organoid biobank, we established a network of five clinical sites in Birmingham, Cambridge, Glasgow, London and Southampton to access patient clinical data and obtain tumour material through surgical resections or biopsies, together with a germline reference normal sample (Fig. 1a and Methods, ‘Ethical approval and sample collection’). We obtained consent to enable broad sharing of models.

Fresh tumour samples were sent to the Wellcome Sanger Institute (May 2016 to November 2023) for organoid derivation in a single centralized facility using standardized operating procedures to minimize variability. A small number of derivations used flash-frozen tissue owing to shutdowns during the COVID-19 pandemic. A parallel sample from the same tumour specimen was acquired for whole-genome sequencing (WGS), enabling later analysis of model fidelity. We focused on five cancer types of unmet clinical need for which organoid derivation protocols were established, in decreasing order of sample numbers received: colorectal adenocarcinomas (COAD/READ), oesophageal adenocarcinomas (ESCA), ovarian carcinomas (OV), pancreatic adenocarcinomas (PAAD) and stomach adenocarcinomas (STAD). We applied stringent criteria for derivation success, requiring expansion to more than 25 million cells to create a bank of 25 cryovials, including the ability to cryopreserve and re-culture organoids (freeze–thaw quality control), and confirmation that their single nucleotide polymorphisms (SNPs) matched the original tumour. An organoid sample was prepared for molecular characterization during banking and freeze–thaw quality control.

Overall, organoid cultures were successfully established for 256 out of 907 samples from 878 unique donors (Supplementary Table 1), yielding an overall efficiency rate of 28% (Fig. 1b). The number of organoids in our biobank exceeds the number of commonly available cell lines for COAD/READ (132 compared with 90 in the Cancer Dependency Map^9^) and includes the cancer types ESCA^15^ and OV^16^, for which there are few existing representative models. Rare tubulovillous and colon tubular adenomas, and ovarian clear cell and mixed cell carcinoma subtypes are also represented in our biobank (Supplementary Table 2).

**Extended Data Fig. 1:**
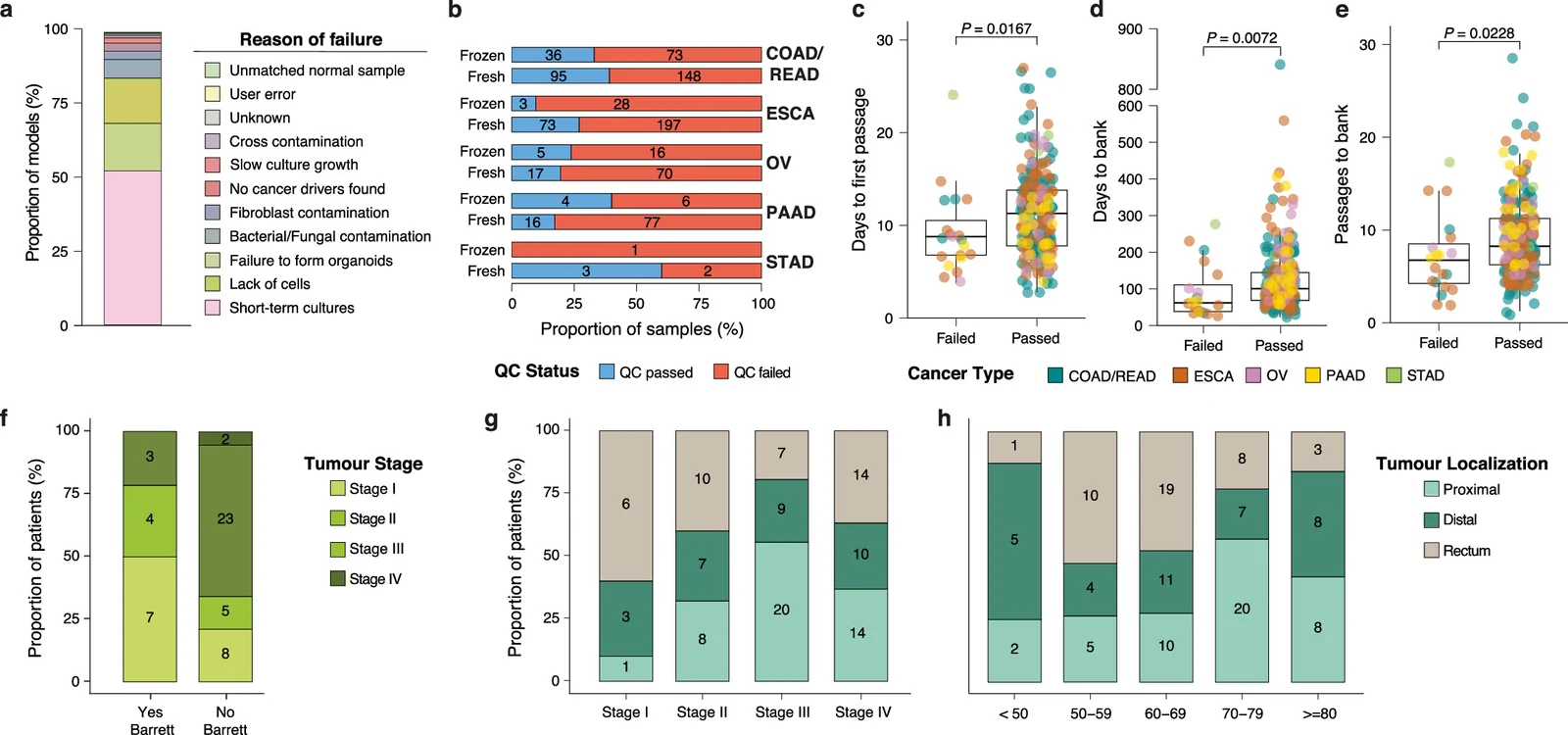
Organoid derivation metrics and clinical data. **a**. Reasons for organoids not being successfully derived (total n = 651). See Supplementary Data Table 1 for a more detailed explanation. **b**. Success rates of organoid derivation by cancer type, stratified by tissue preservation method. Samples with undocumented preservation status were excluded from the analysis. The numbers in the bars represent the number of organoids attempted to be derived. **c, d** and **e**. Comparison of three key lab metrics during the derivation process: days to the first passage (average 9.45 vs 11.57), days needed to bank (average 6.90 vs 8.79) and passages needed to bank (average 86.85 vs 120.34), between quality control (QC) failed (n = 20) and QC passed models (n = 256) (only including banked organoids). For panels c-e, two-sided Wilcoxon rank-sum test was used, with pairwise comparisons and FDR correction. Box plots display the median, first and third quartiles (boxes), and whiskers are 1.5x the interquartile range from the first and third quartiles. **f**. Association between stage and presence of Barrett’s oesophagus on oesophageal adenocarcinomas (ESCA). **g**. Association between tumour localisation and stage of colorectal adenocarcinomas (COAD/READ). **h**. Association between tumour localisation and age of patients with COAD/READs. Only organoids with completed data for the parameters compared in each analysis were used for the visualisation and the statistical test. For panels f-h, a two-sided Fisher’s exact test was applied with the p values shown in the text. OV: ovarian carcinoma; PAAD: pancreatic adenocarcinoma; STAD: stomach adenocarcinoma; Proximal: from the cecum to the proximal two-thirds of the transverse colon; Distal: from the descending colon to the rectum.

Derivation at a single facility enabled assessment of parameters associated with success (Extended Data Fig. 1 and Supplementary Table 1). The most common reasons for model failure were the development of non-renewable short-term cultures and insufficient tumour cellularity (labelled ‘lack of cells’; Extended Data Fig. 1a). When we account for these factors, by including short-term cultures and excluding samples with low cellularity, our success rate was 65%, comparable to previous studies. Success rates were similar between fresh and frozen tissue (Extended Data Fig. 1b). Once banked, 93% of cultures passed a freeze–thaw quality control step (Fig. 1b), supporting the effectiveness of our protocols to generate renewable cultures. Early passaging and cryopreservation (days to passage: *P* = 0.0167; days to bank: *P* = 0.0072; passages to bank: *P* = 0.0228; Wilcoxon rank-sum test) were unexpectedly associated with banking failure, potentially owing to the dominant outgrowth of non-tumour cells (Extended Data Fig. 1c–e).

**Extended Data Fig. 2:**
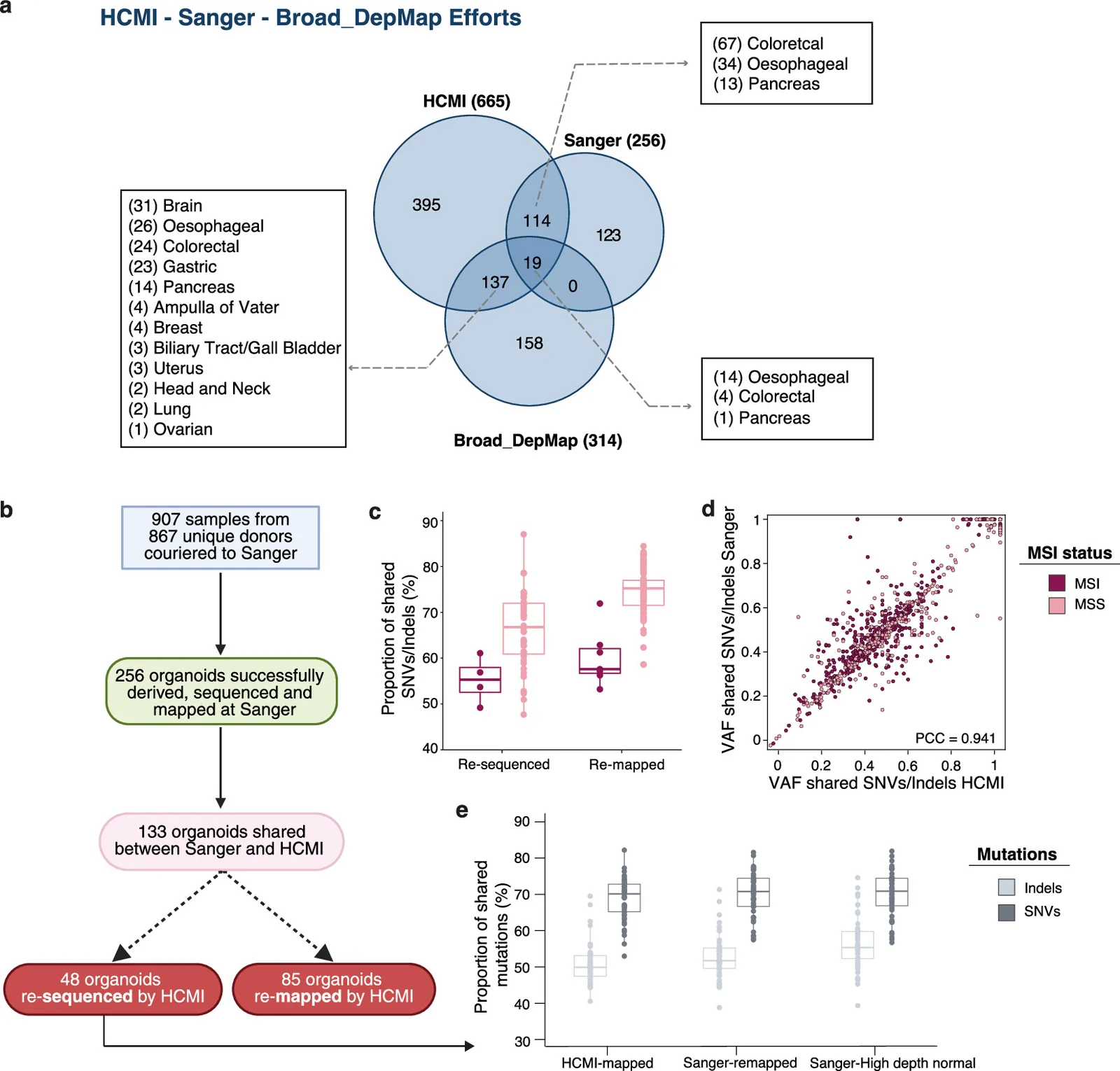
HCMI-Sanger-Broad_DepMap overlap and comparison of organoid genomic data between HCMI and Sanger. **a**. Venn diagram showing the overlap between the Human Cancer Models Initiative (HCMI), Broad DepMap next-generation models, and this study. **b**. Flowchart illustrating whole genome sequencing (WGS) data across a subset of organoids shared by Sanger to HCMI. Shared samples were either re-mapped by HCMI from the same BAM files, or the same DNA was re-sequenced by HCMI. **c**. The proportion of single nucleotide variants (SNVs) and insertions/deletions (Indels) shared between organoids analysed by Sanger and HCMI (re-sequenced, n = 48; re-mapped, n = 85). **d**. Comparison of variant allele frequencies (VAFs) of shared SNVs and Indels in cancer driver genes between Sanger and HCMI, with the Pearson correlation coefficient (PCC) indicated. **e**. The proportion of SNVs and Indels shared between organoids sequenced at Sanger or re-sequenced HCMI (n = 48), and analysed using either the HCMI or Sanger pipelines, with high-depth blood samples from Sanger used as controls. Box plots display the median, first and third quartiles (boxes), and whiskers are 1.5x the interquartile range from the first and third quartiles.

A subset of 133 models was derived as part of the HCMI (Fig. 1c and Extended Data Fig. 2a), and these samples were independently genetically analysed in an accompanying HCMI paper^17^ (Extended Data Fig. 2b). We observed high concordance of somatic variants from these independent analyses (Extended Data Fig. 2c,d), with some discrepancy for detection of insertion–deletion mutations (indels), largely attributable to differences in sequencing coverage (Extended Data Fig. 2e; average coverage: Sanger, 38×; HCMI, 19×). These findings highlight the robustness and intercompatibility of results from both studies.

Thus we have developed one of the most extensive biobanks of long-term renewable patient-derived 3D organoids across five cancer types. Organoids are being distributed through non-profit and commercial repositories to enable open sustainable community access (Fig. 1c and Methods, ‘Availability of Organoid Biobank’). An inventory of all derived models, their unique identifiers and molecular and functional characterization is provided (Supplementary Table 2).

## Clinical data ensure biobank diversity

The lack of patient and clinical data for most widely used cancer cell lines limits their utility. Here, we acquired patient and clinical annotations for each model (ranging from 25 to 77, depending on cancer type and patient; Supplementary Table 2) (Fig. 1d). COAD/READ and ESCA organoid cultures were mostly from male patients, consistent with incidence rates^18,19^. Our biobank includes organoids from patients with early (stage I and II; *n* = 83) and advanced (stage III and IV; *n* = 131) pathological staging (Fig. 1d). The biobank was derived from primary tumours (*n* = 212) and metastatic lesions (*n* = 44) (Fig. 1d), providing a valuable resource to study metastatic disease biology. Some patients had received prior treatment (*n* = 122), which was predominantly chemotherapy (*n* = 119), consistent with standard-of-care treatment, with 8 patients also receiving targeted immunotherapy, enabling the study of treatment resistance (Fig. 1d). Nearly 60% of patients with ESCA were smokers or ex-smokers, a known risk factor^20^ (Fig. 1d), and patients undergoing surveillance for Barrett’s oesophagus had earlier stage ESCA (Extended Data Fig. 1f, *P* = 0.0375, chi-square test). A total of 17 organoids were derived from patients less than 50 years of age (Fig. 1d), including 11 with COAD/READ. Early-onset colorectal cancer is a leading cause of cancer death in young adults, with increasing incidence rates, underscoring the value of these models for studying this emerging clinical challenge^21^. Organoids from patients with advanced stage COAD/READ were more frequent on the proximal site (Extended Data Fig. 1g, *P* = 0.1761, Fisher’s exact test), as were tumours present in older patients (over 70 years old), consistent with clinical presentation^22^ (Extended Data Fig. 1h, *P* = 0.0165, Fisher’s exact test).

**Extended Data Fig. 3:**
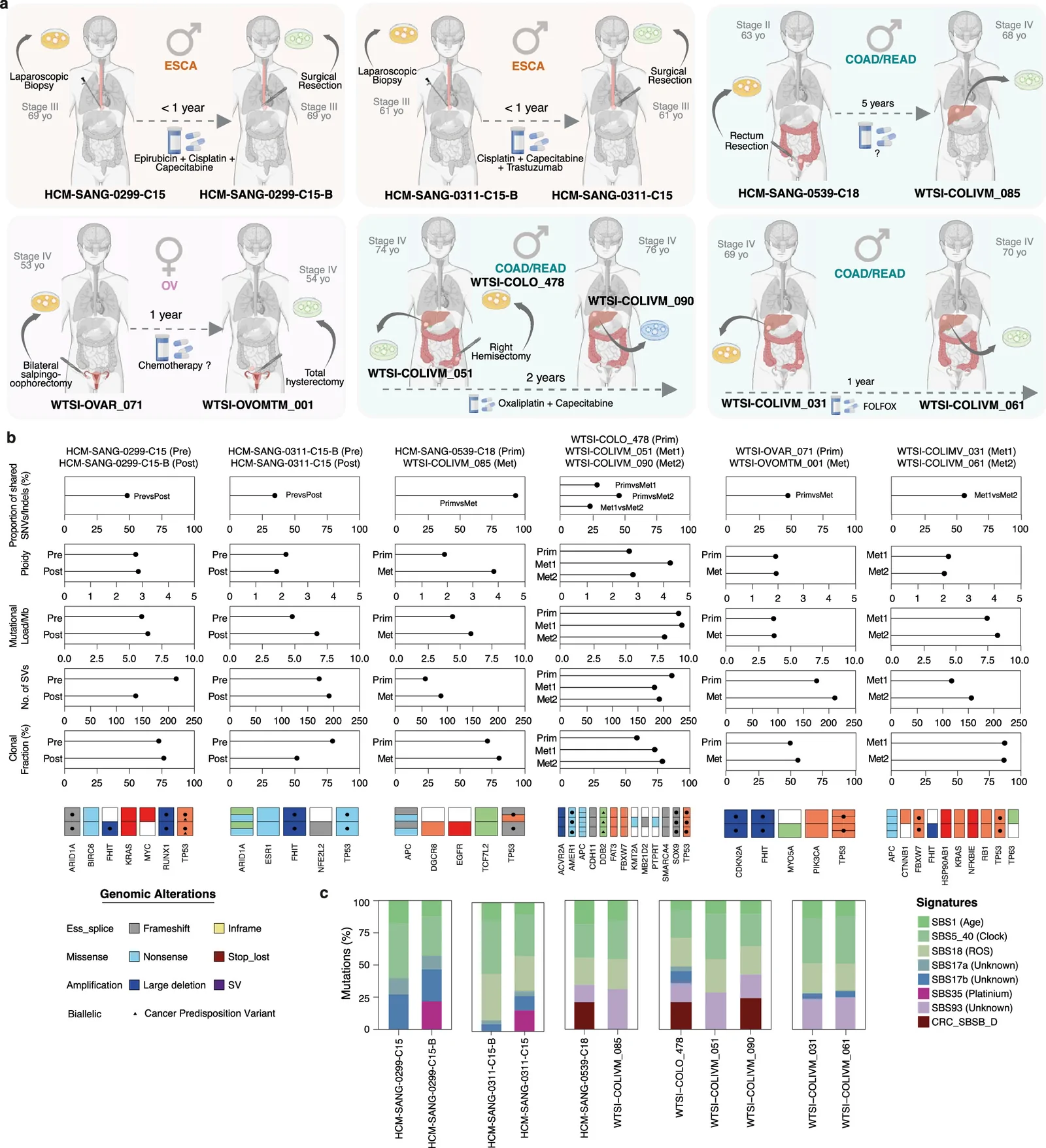
Pairs of organoids derived from the same patient. **a**. Schematic representation of organoid pairs derived from tissues obtained from the same patient. Information displayed includes cancer type, organoid ID, patient age at biopsy, sample collection procedure, patient sex, tumour stage, time interval between sample collections, and any treatments received between biopsies (if applicable). “?” in panels c and d means that the treatment used is unknown. Background color signifies tumour type. COAD/READ: colorectal adenocarcinoma (turquoise background); ESCA: oesophageal adenocarcinoma (light brown background); OV: ovarian carcinoma (pink background). Illustration created in BioRender; Herranz-Ors, C. https://BioRender.com/hl5osob (2026). **b**. The proportion of shared SNVs, ploidy levels, mutational load per megabase (Mb), number of structural variants (SVs), and clonal fraction for each organoid in the pairs. Comparisons of mutations and large somatic copy number alterations (SCNAs) in driver genes are also shown, with biallelic alterations indicated by dots and cancer predisposition variants marked by triangles over the respective gene alterations. Pre: pre-treatment; Post: post-treatment; Prim: primary tumour; Met (1-2): metastasis. On the y-axes, organoids are ordered as panel a. **c**. The proportion of mutations corresponding to each mutational signature in organoids derived from the same patient. Signature CRC_SBSB_D was absent from WTSI-COLIVM_051, potentially due to miscalling of SBS93 or clonal selection at the metastatic site.

The biobank includes 13 patient-matched organoids from 6 patients, including 4 pre- and post-treatment paired samples, 3 organoids from one patient’s primary tumour and 2 sequential liver metastases, and 2 organoids from one patient’s matched liver metastases collected 1 year apart (Extended Data Fig. 3a).

Overall, the clinical information associated with each organoid—including disease stage, risk factors and prior treatment—enhance the relevance of the biobank, enable rational model selection and support integration with external clinical datasets for deeper interpretation.

## Organoids preserve tumour genomics

To further inform the usefulness of our biobank, we performed a comprehensive genomic characterization of organoids and their patient-matched tumour sample using WGS, as well as RNA sequencing (RNA-seq) of the organoid (Fig. 1c). Single nucleotide variants (SNVs), indels, structural variants (SVs), somatic copy number alterations (SCNAs), mutational signatures, chromothripsis and whole-genome duplication (WGD) events were identified. WGS is available for all 256 organoids (Supplementary Table 2), together with the corresponding normal tissue sample as a germline reference to enable accurate calling of somatic variation, overcoming a major limitation of most 2D cell lines. For 171 organoids, sufficient tumour tissue was available for sequencing (Fig. 1c and Supplementary Table 2), enabling a direct comparison of organoid to tumour.

**Fig. 2:**
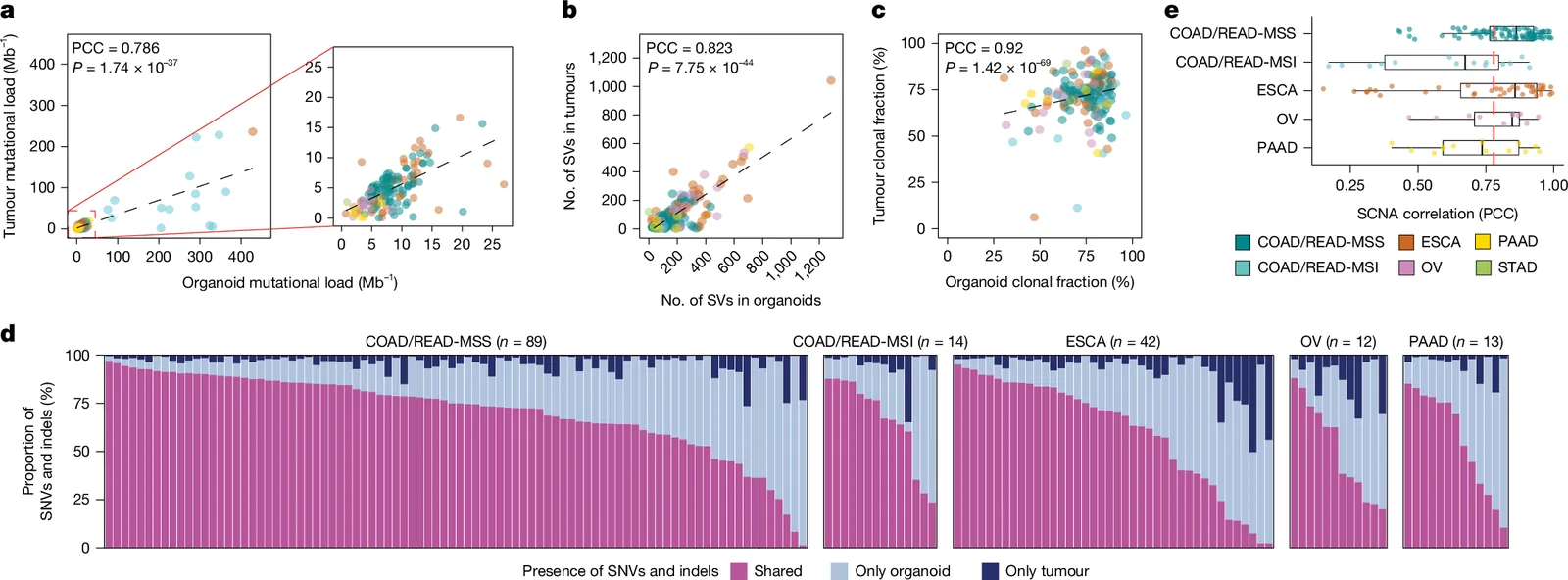
Genomic features are conserved between organoid and patient-matched tumour. **a**, Pearson correlation between mutational load per megabase in organoids and tumours. Right magnified view of the indicated region of the main graph. **b**, Pearson correlation between number of SVs in organoids and tumours. **c**, Pearson correlation between clonal fraction in organoids and tumours. **d**, Proportion of SNVs and indels that are shared between organoids and tumours, or exclusive to each. **e**, Box plots showing PCC of the SCNA comparisons at the whole-genome level between organoid and tumour pairs. Box plots display the median (centre line), first and third quartiles (box edges) and whiskers extend to 1.5× the interquartile range from the first and third quartiles. The red dashed line indicates the median of all the data points. Dots are coloured by cancer type. All available organoid–tumour pairs (*n* = 171) were sequenced once.

**Extended Data Fig. 4:**
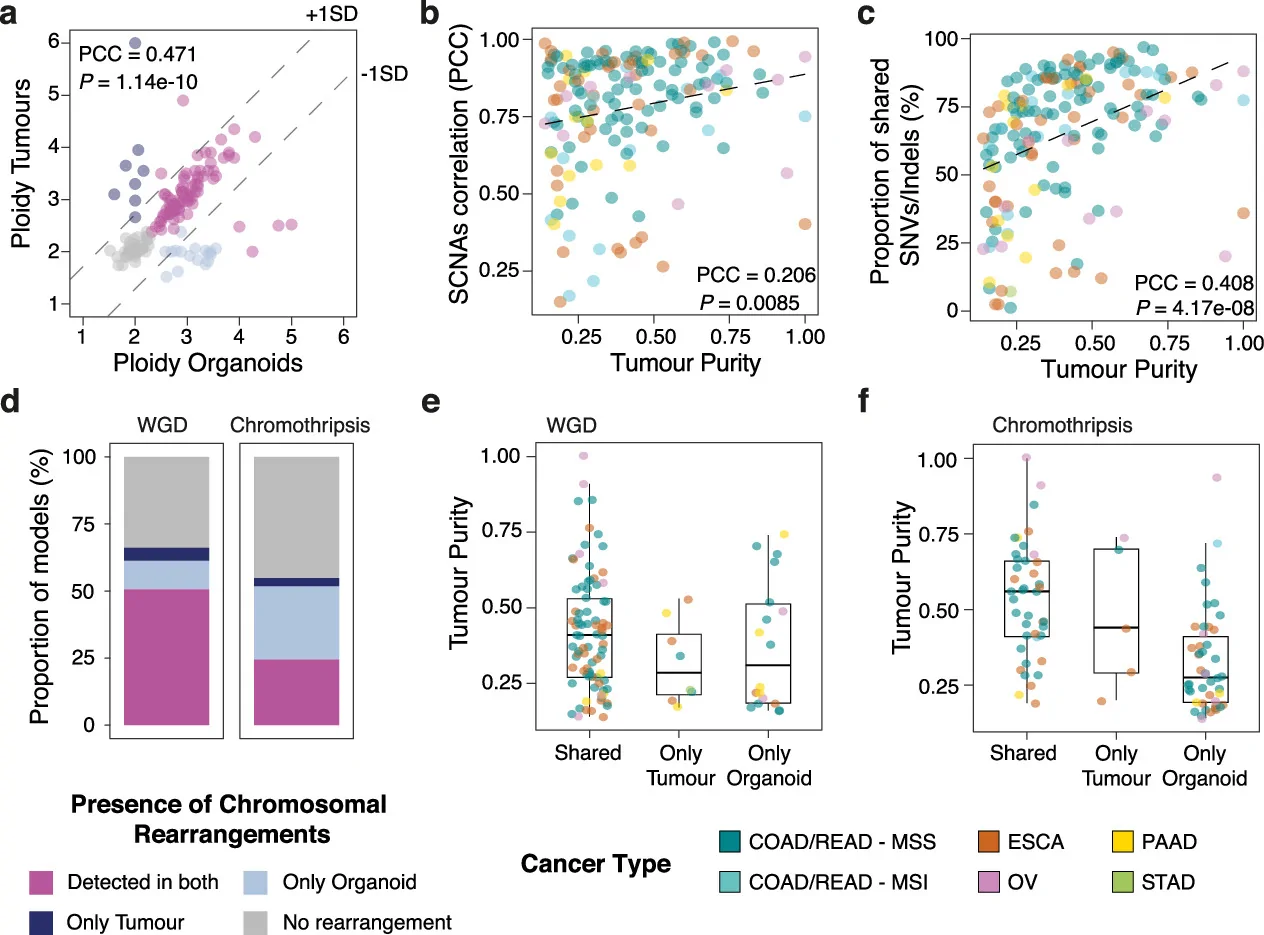
Correlation between tumour purity and concordance between organoids and tumours. Pearson correlations between **a**. Ploidy levels in organoids (x-axis) and tumours (y-axis), with dots coloured to indicate the presence of whole genome duplication (WGD) based on panel d. Dashed lines indicate data within 1 standard deviation (SD); **b**. Tumour purity (x-axis) and SCNAs (y-axis); and **c**. Tumour purity (x-axis) and proportion of shared SNVs and Indels between organoids and matched-tumours (y-axis). **d**. Proportion of models with available tumour whole genome sequencing (WGS) data, categorised by the presence of WGD or chromothripsis in both organoid and tumour, only in organoids, only in tumours, or lacking such rearrangements. **e**. The association between WGD presence and tumour purity. **f**. The association between chromothripsis and tumour purity. Box plots display the median, first and third quartiles (boxes), and whiskers are 1.5x the interquartile range from the first and third quartiles. All available organoid–tumor pairs (n = 171) were sequenced once. COAD/READ: colorectal adenocarcinoma; ESCA: oesophageal adenocarcinoma; OV: ovarian carcinoma; PAAD: pancreatic adenocarcinoma; STAD: stomach adenocarcinoma; MSS: microsatellite stability; MSI: microsatellite instability.

To assess model fidelity, we compared organoid cultures with their patient-matched tumour samples across multiple genomic features (Supplementary Table 3). Given the substantial genomic and clinical differences between COAD/READ samples with microsatellite instability (MSI) or microsatellite stability (MSS), we analysed these separately. The correlation for mutational load was high (Pearson correlation coefficient (PCC) = 0.786; Fig. 2a), as was the correlation for SVs (PCC = 0.823; Fig. 2b). The clonal fractions, which denote the proportion of mutations within a cancer cell fraction (CCF) above 0.75, were well correlated but higher in organoids (PCC = 0.92; Fig. 2c), indicating that most mutations are clonal and that intratumour heterogeneity is conserved. Concordance was also strong at the SNV and indel level, with 76 pairs (44%) sharing at least 75% of somatic mutations (Fig. 2d). Genome-wide SCNA profiles also showed high correlation (median PCC = 0.78), with 142 pairs (83%) achieving a PCC of at least 0.5. Correlations were lower in COAD/READ with MSI, a subtype that was not driven by SCNAs (Fig. 2e). For ploidy comparisons, 139 pairs (81%) exhibited ploidy differences within one standard deviation (s.d. = 0.697; Extended Data Fig. 4a). High SCNA correlation or an increased proportion of shared SNVs and indels between organoids and matched tumours was positively associated with higher tumour purity (Extended Data Fig. 4b,c), suggesting that concordance could be underestimated when tumour purity is low. Chromothripsis and WGD were more frequently detected in organoids than in tumours, and concordance between matched samples increased with tumour purity (Extended Data Fig. 4d–f). Finally, we compared patient-matched organoids and found them to be largely comparable, with some minor differences in SNVs and high conservation of cancer driver events (Extended Data Fig. 3b).

Together, these findings confirm that our organoids faithfully recapitulate a wide range of genomic features from the tumour from which they were derived, supporting their relevance for disease modelling. The higher tumour cellularity of organoids and potentially reduced heterogeneity due to clonal pruning during culturing lead to greater sensitivity to detect genomic alterations in organoid cultures than in tumours.

## Organoids mirror driver landscapes

**Fig. 3:**
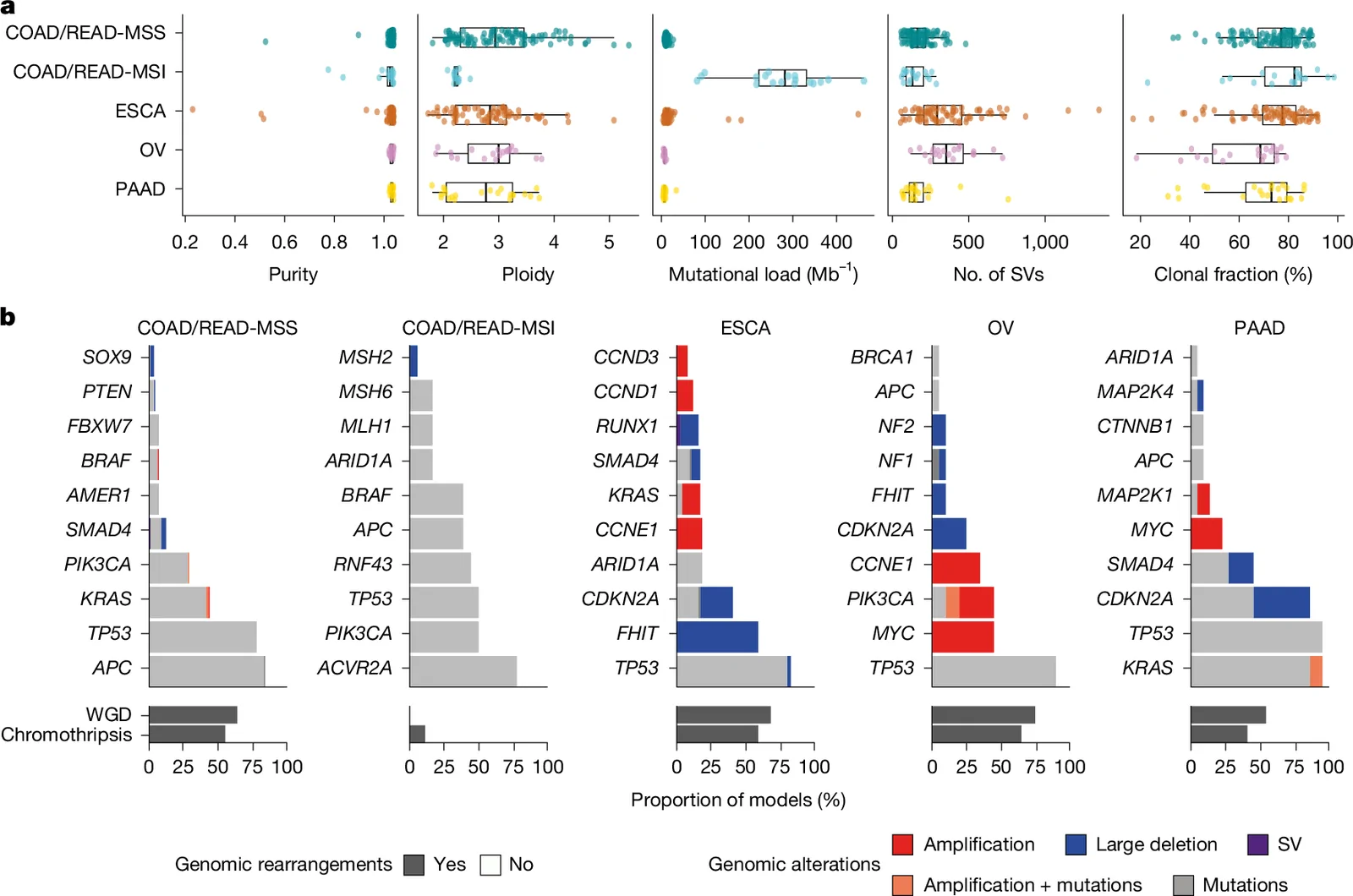
Genomic landscape and cancer-specific drivers in the organoid biobank. **a**, The purities, ploidies, mutational loads, number of SVs and clonal fraction of organoids for each cancer type. Box plots display the median (centre line), first and third quartiles (box edges) and whiskers extend to 1.5× the interquartile range from the first and third quartiles. **b**. Proportions of models with alterations in the ten most frequent or curated informative genes, along with the proportions of models displaying chromothripsis or WGD across cancer types. Only biallelic inactivations are shown for tumour suppressor genes and *TP53*. All available organoids (*n* = 256) were sequenced once.

Having confirmed the fidelity of organoid cultures to their original tumour, we next examined in detail these genomic features of the organoids by tumour type (excluding STAD organoids owing to low numbers; Supplementary Table 3). Almost all organoids had more than 0.95 tumour purity, consistent with a highly enriched tumour cell culture (Fig. 3a). Most COAD/READ-MSI models were diploid as expected^23^, whereas other cancer types had greater ploidy variation^24^ (median = 2.5; Fig. 3a). MSI models had a high mutational load (Fig. 3a). ESCA and OV organoids had the highest number of SVs (Fig. 3a). Clonal fractions were overall high across the models, especially for COAD/READ-MSI organoids, indicating a significant degree of clonality. However, organoids with clonal fractions below 50% may exhibit intra-organoid heterogeneity (Fig. 3a).

**Extended Data Fig. 5:**
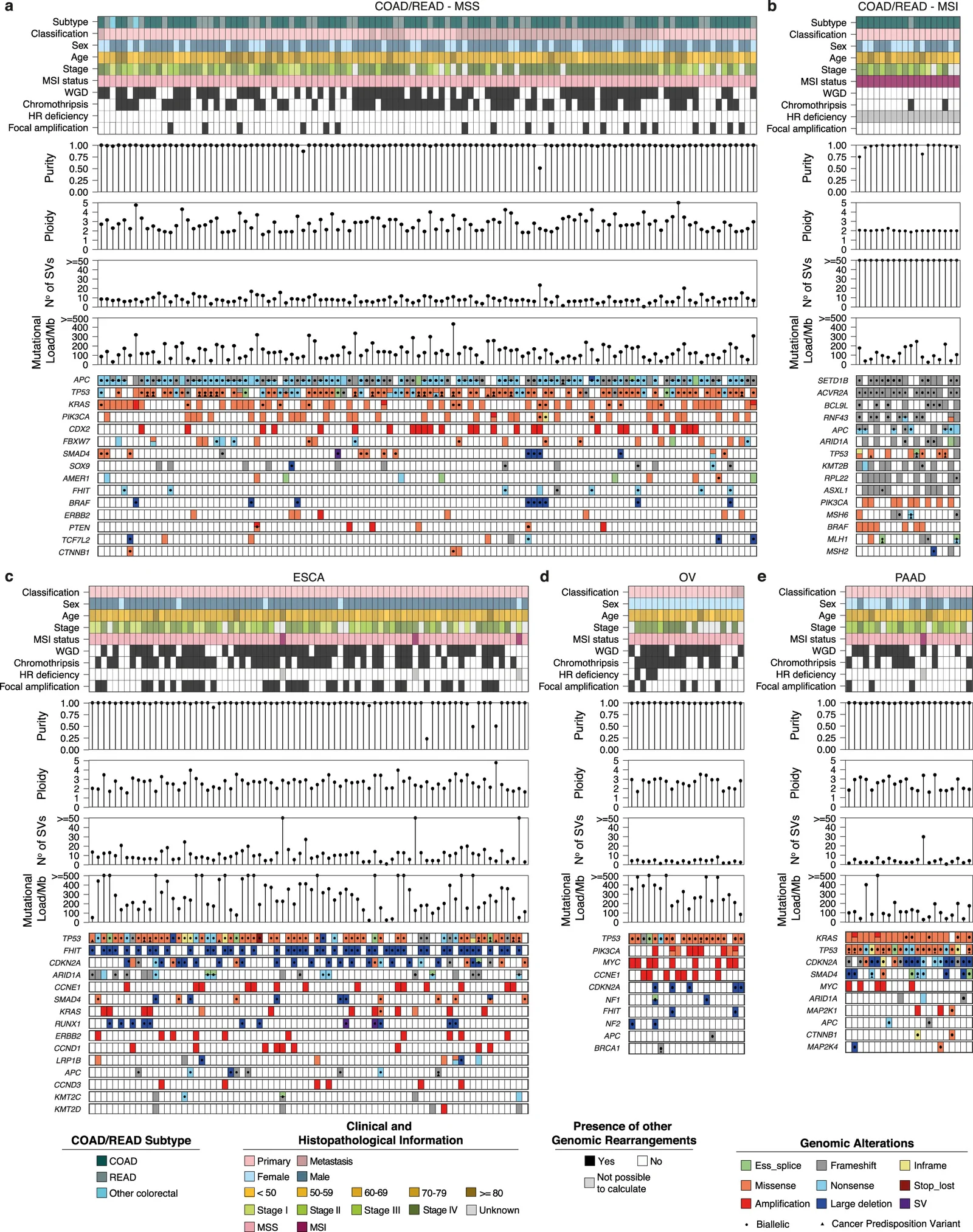
Comprehensive genomic and clinical landscape of organoids in the biobank. **a-e**. Clinical and genomic information for organoids by cancer type. Displayed information includes clinical data, histopathological features, whole genome duplication (WGD), presence of chromothripsis, homologous recombination (HR) deficiency and focal amplifications (1st section), tumour purity (2nd section), ploidy (3rd section), number of structural variants (SVs) (4th section), mutational load per megabase (Mb) (5th section), and mutations and somatic copy number alterations (SCNAs) in driver genes (6th section). Biallelic alterations are indicated by dots, and cancer predisposition variants are marked by triangles over the genomic alterations of the respective genes. COAD: colon adenocarcinoma; READ: rectum adenocarcinoma; ESCA: oesophageal adenocarcinoma; OV: ovarian carcinoma; PAAD: pancreatic adenocarcinoma; MSS: microsatellite stability; MSI: microsatellite instability.

**Extended Data Fig. 6:**
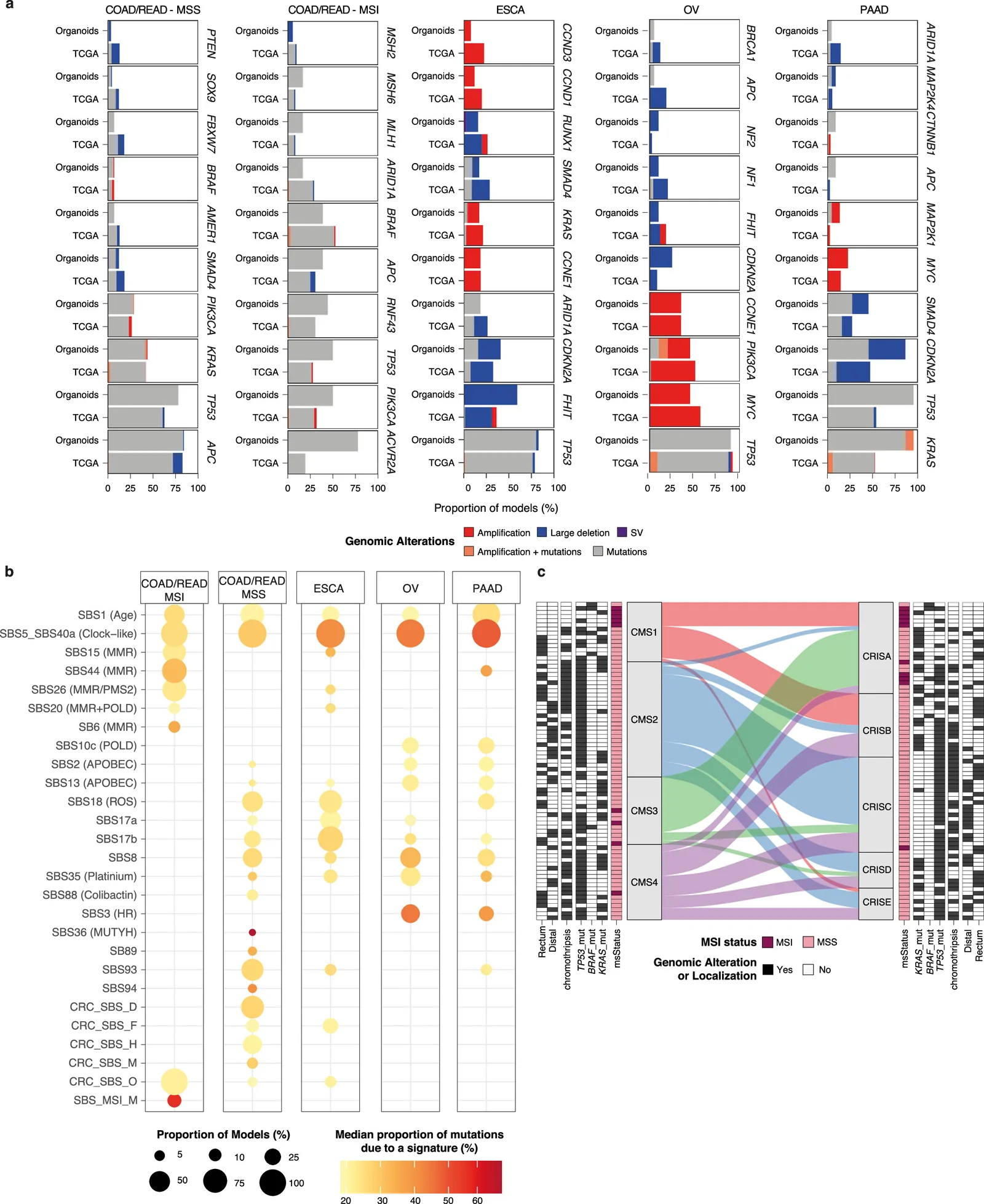
Genomic and transcriptomic summaries. **a**. Proportion of organoids and The Cancer Genome Atlas Program (TCGA) samples with alterations in the same genes shown in Fig. 3b. There are some discrepancies between PAAD organoids and TCGA samples for *KRAS*, *TP53* and *CDKN2A* genes. The frequencies observed in organoids align more closely with results from the Pan-Cancer Analysis of Whole Genomes (PCAWG) study^34^. COAD/READ: colorectal adenocarcinoma; ESCA: oesophageal adenocarcinoma; OV: ovarian carcinoma; PAAD: pancreatic adenocarcinoma; MSS: microsatellite stability; MSI: microsatellite instability; SV: structural variant. **b**. The distribution of single base substitution (SBS) mutational signatures across cancer types. The dot size represents the proportion of models of each cancer type exhibiting a given mutational signature, and the dot colour indicates the median number of mutations for each signature relative to the total mutations per sample within each cancer type. Only signatures constituting at least 10% of the mutations in a sample were included, and samples poorly reconstructed by signature assignment were excluded. **c**. Consensus molecular subtypes (CMS) and colorectal cancer intrinsic subtypes (CRIS), and their inter relatedness. Only COAD/READ models with a subtype assigned were used (n = 79, unknown subtype removed). The size of the nodes reflects the number of models belonging to a particular subtype. MSI status, *KRAS*, *BRAF*, *TP53* mutation, presence of chromothripsis and tumour localisation were shown for each organoid along with a CMS and CRIS subtype assignation. Fisher’s exact test was applied as a part of a co-occurrence/mutual-exclusivity analysis and the p values were shown in the text.

Lineage-specific cancer driver events reported in public cancer datasets^25^ were recapitulated in the organoid biobank at comparable frequencies (Fig. 3b and Extended Data Figs. 5 and 6a). Complex rearrangements and SCNAs were observed with tumour-type-specific patterns consistent with patient tumours (Fig. 3b). COAD/READ-MSI models had almost no complex rearrangements, whereas OV organoids had the highest frequency of WGD and chromothripsis^26^.

A key feature of our large biobank is representation of inter-patient heterogeneity, including rare but clinically important genotypes that are underrepresented or absent in cancer cell line collections^9^ (Extended Data Fig. 5). Our biobank also includes rare models with complex genotypes, such as five COAD/READ-MSS models that all carry *APC*, *TP53*, *KRAS* and *SMAD4* mutations or one with *APC*, *TP53*, *KRAS* and *PIK3CA* mutations together with a *PTEN* deletion, which will enable studies investigating acquisition of mutations during multi-step carcinogenesis^27,28^. Our biobank includes 94 *KRAS*-altered organoids, including specific variant alleles that varied in frequency by tumour type, consistent with patient tumours^29^, thereby enabling the study of this clinically important molecular cohort (Extended Data Fig. 5).

We used our WGS data to uncover the landscape of mutational signatures available in the Catalog of Somatic Mutations in Cancer (COSMIC) database^30^ in organoids, providing insights into disease aetiology and patient clinical history (Extended Data Fig. 6b and Supplementary Table 3). For example, all MSI models had signatures related to DNA mismatch repair deficiency (SBS15, SBS44, SBS26, SBS20, SBS6 and/or the recently described SBS_MSI_M). SBS88, caused by the bacteria-produced mutagen colibactin^31,32^, was detected in 10 COAD/READ-MSS organoids, including those from three early-onset cases, representing 27% of samples from patients with COAD/READ who were diagnosed before the age of 50. Notably, 9 out of 10 SBS88-positive organoids originated from distal (*n* = 4) or rectal (*n* = 5) tumours^33^. We identified six organoids with SBS3, a signature linked to homologous recombination deficiency (HRD), some of which overlapped with models detected as HRD by CHORD, which integrates multiple types of somatic mutations to detect HRD^34^ (Extended Data Fig. 5). We detected SBS35, associated with previous platinum treatment, in organoids from donors treated with this chemotherapeutic agent (Extended Data Fig. 3c). The high mutational load of cancer cell lines, coupled with the absence of matched normal samples for germline filtering, complicates accurate detection of mutational signatures^35^. By contrast, organoid cultures offer a promising model for studying signature biology, capturing aspects of disease aetiology including genetic factors and clinically relevant exposures.

RNA-seq of the organoids was performed, enabling us to classify the COAD/READ models into two of the most validated and clinically relevant transcriptional subtypes: consensus molecular subtypes^36^ (CMS 1–4) and colorectal cancer intrinsic subtypes (CRIS A–E)^37^ (Supplementary Table 3). CMS and CRIS classifications showed strong concordance in colorectal cancer organoids and were associated with distinct genomic and anatomical features, with CRIS providing more precise correlations with specific molecular alterations (Extended Data Fig. 6c).

**Extended Data Fig. 7:**
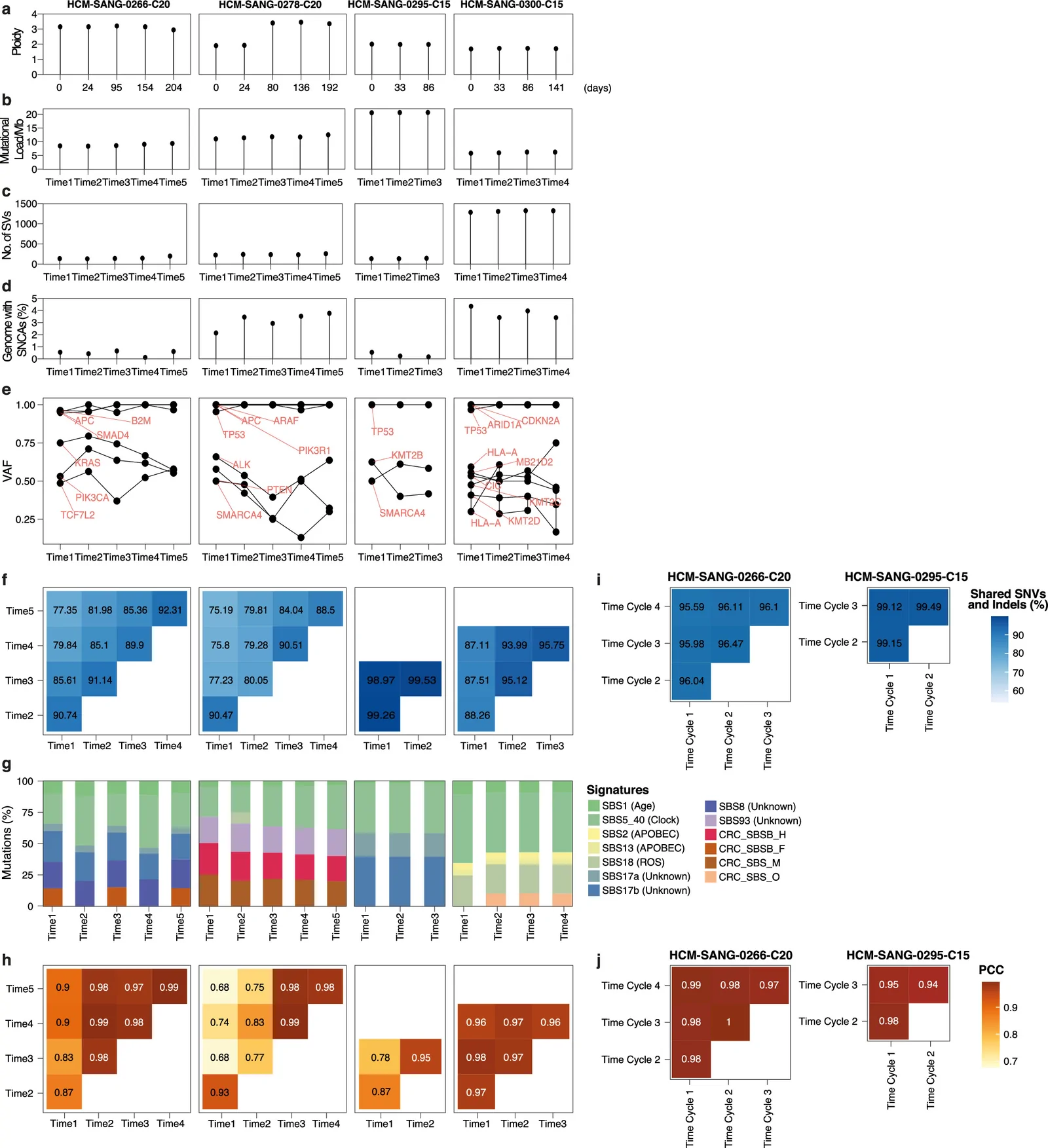
Organoid genomic and transcriptomic features are stable following prolonged culturing or multiple freeze-thaw cycles. Evolution of **a**. ploidy, **b**. number of mutations per megabase (Mb), **c**. number of structural variants (SVs), and **d**. percentage of the genome with somatic copy number alterations (SCNAs) along time-points for four organoids. **e**. Variant allele frequency (VAF) of cancer driver mutations. **f**. Proportion of shared single nucleotide variants (SNVs) and insertions/deletions (Indels) across all possible pairwise comparisons. **g**. Proportion of mutations that represent each mutational signature in each time-point. For HCM-SANG-0266-C20 at times 2 and 5, CRC_SBSB_F is likely mis-called as SBS5_40, a known ‘sponge’ signature. Evidence of CRC_SBS_O is observed at later time points for HCM-SANG-0300-C15. **h**. Pearson correlation coefficients (PCC) across all possible pairwise comparisons using RNA-seq data for all genes. See Supplementary Methods - ‘Longitudinal and freeze/thaw-cycles samples’ for the duration of each time point. **i**. Proportion of shared SNVs and Indels across all possible pairwise comparisons along freeze-thaw cycles for two organoids. **j**. Pearson correlation coefficients (PCC) across all possible pairwise comparisons using RNA-seq data for all genes. See Supplementary Methods - ‘Longitudinal and freeze/thaw-cycles samples’ for the duration of freeze-thaw cycles.

Whole-genome and transcriptomic profiling showed that organoids maintained stable ploidy, mutational landscapes and transcriptional profiles over up to six months of culture and multiple freeze–thaw cycles (Extended Data Fig. 7 and Supplementary Information). Long-term culture caused slightly greater—but still modest—genetic drift than repeated freeze–thaw cycles. These results demonstrate that organoids largely preserve genomic integrity over time^38,39^, supporting their use as robust and renewable tumour models.

These comprehensive genomic and transcriptomic analyses underscore the utility of organoids in reflecting inter-tumour heterogeneity, lineage-specific driver mutations, mutational patterns and transcriptional programs associated with disease and that can be linked to clinical parameters. This study is one of the most comprehensive genomic analyses of organoids performed to date, both in terms of number of models and depth of characterization. To facilitate accessibility of these data, we have made them available through the Cell Model Passports database^40^ (https://cellmodelpassports.sanger.ac.uk/).

## Mapping cancer dependencies in organoids

Dependency catalogues from pharmacological and genetic screens, including the Cancer Dependency Map, have advanced target discovery and patient stratification. Yet existing cell models do not capture the full tumour diversity (for example, in ESCA), and inter-tumour heterogeneity limits genotype–dependency associations. High mutational burden and lack of matched germline data further complicate linking dependencies to genomic features. Systematic dependency mapping in organoids could address these gaps. However, CRISPR screens in 3D cultures remain limited^41–44^, as the increased complexity of screening in 3D cultures across tissue histologies is constrained in scale. To overcome this, we established a scalable platform to map dependencies across our organoid biobank.

**Extended Data Fig. 8:**
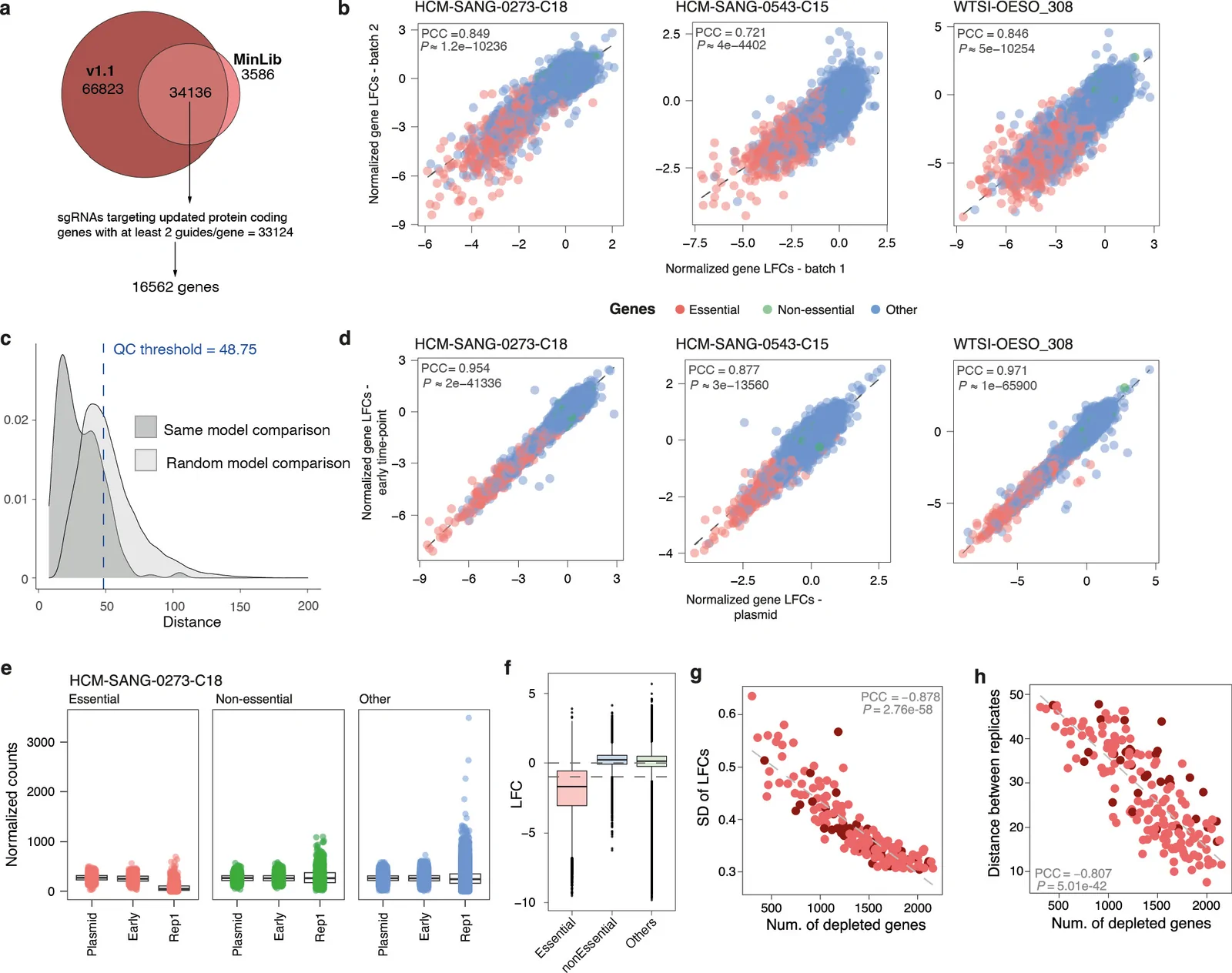
CRISPR-Cas9 screening quality control metrics. **a**. Overlap of single guide RNAs (sgRNAs) in the Human CRISPR-Cas9 Libraries Yusa v.1.1 (v1.1) and the Minimal Genome-Wide (MinLibCas9). Only genes with at least two shared sgRNAs were included in the analysis. **b**. Pearson correlation between two independent batches of Cas9-expressing organoids. Normalised gene log fold changes (LFCs) values are shown for screens performed in three independent organoid cultures. **c**. Quality control (QC) threshold based on distribution of distances between sgRNA fold changes of replicates from the same organoid (dark grey) and random pairs (light grey), considering the best correlated 800 sgRNAs. **d**. Pearson correlation between screens performed in three organoids using gene LFCs normalised to the sgRNA library plasmid (x-axis) versus a five days post transduction (early timepoint) control (y-axis). Genes are grouped by whether they target a known essential, non-essential or other gene. **e**. Comparison of normalised read counts in the plasmid or early time point control, or at the end of screening (Rep1 shown; total technical replicates, n = 3). Data are representative of three organoids. **f**. The distribution of CRISPRcleanR normalised and corrected LFCs in essential, non-essential, and other genes. Data from all organoids (*n* = 162) are shown. Light grey dashed lines indicate −1 and 0 LFCs. **g** and **h**. Pearson correlation between the number of genes considered depleted by BAGEL2 (x-axis), and the standard deviation (SD) of LFCs or the distance between replicates (y-axis). Dot colour indicates the library tested in each model. Pearson correlations are shown where relevant. All box plots display the median, first and third quartiles (boxes), and whiskers are 1.5x the interquartile range from the first and third quartiles.

Organoids were screened using a 5% basement membrane extract-2 (BME-2) suspension culture, which simplifies handling while preserving genetic and phenotypic stability^38^. Screening was conducted using the minimal genome-wide CRISPR–Cas9 library^41^ (MinLibCas9), alongside the larger Human CRISPR Library Yusa v.1.1 (refs. ^3,9^) (hereafter, Yusa v1.1) for a subset of models (Extended Data Fig. 8a). MinLibCas9 required fewer cells than Yusa v1.1 and, combined with suspension culture, increased efficiency of dependency mapping in organoids to enable systematic screening.

**Fig. 4:**
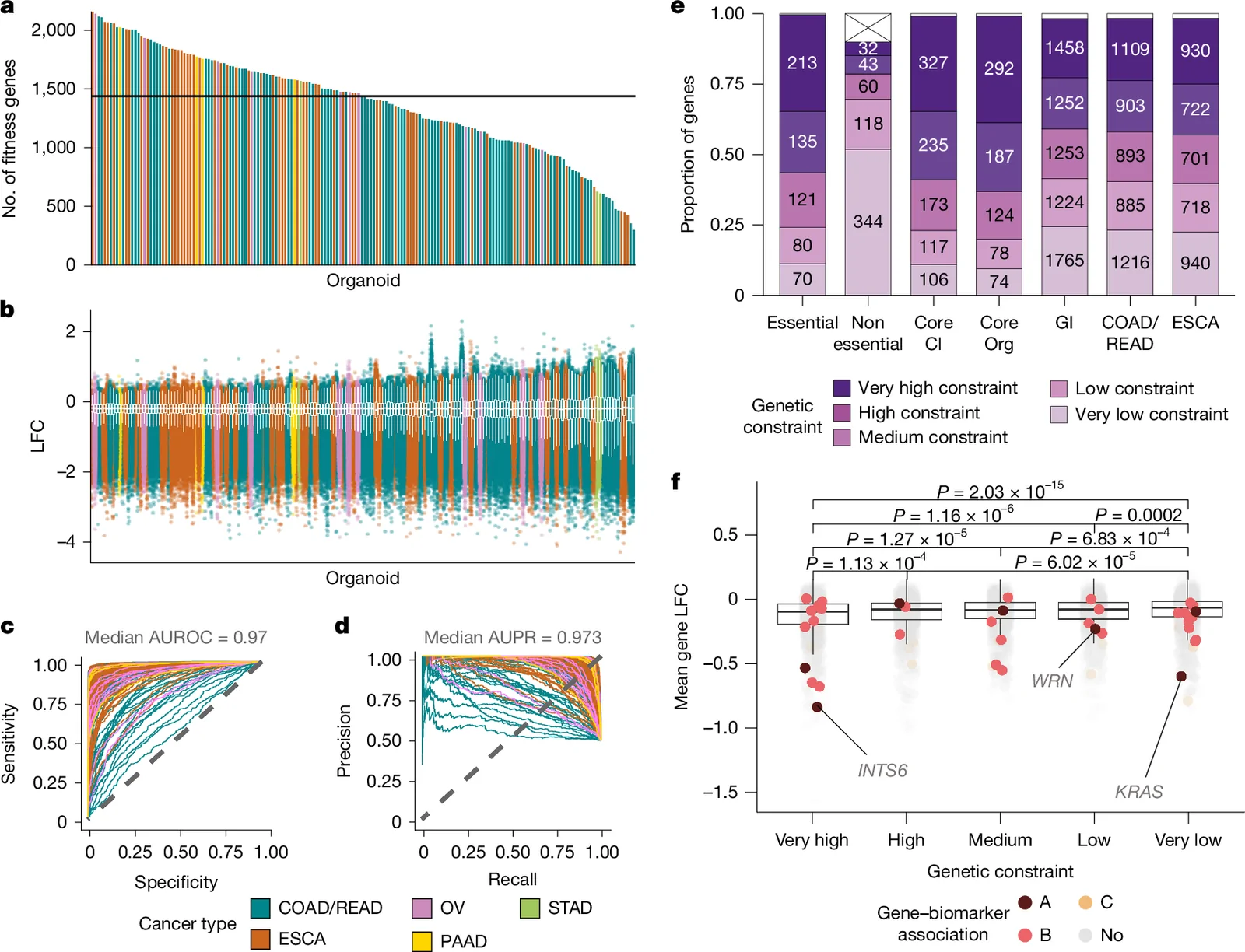
Multi-omic analysis for context-specific organoid dependencies. **a**,**b**, Number of fitness genes identified (**a**) and gene knockout scaled LFC values (**b**) for 162 organoids coloured by cancer type. **c**,**d**, AUROC (**c**) and AUPR (**d**) obtained after classifying predefined essential and non-essential genes using CRISPRcleanR normalized and corrected LFCs. **e**, Genetic constraint annotation for gene sets, including reference essential and non-essential genes, core fitness genes identified in cell lines (Core Cl), core fitness genes identified in organoids (Core Org), and genes analysed in biomarker analyses restricted to colorectal (COAD/READ, *n* = 85), oesophageal (ESCA, *n* = 59) or all gastrointestinal organoids (GI; *n* = 151). **f**, Mean LFC values of differentially dependent genes for all gastrointestinal organoids, grouped by genetic constraint buckets. Dot colour indicates the significance of gene–biomarker associations (class A is more significant than class B, which is more significant than class C); ‘No’ indicates that no biomarker is associated. A two-sided Wilcoxon rank-sum test was used, with pairwise comparisons and FDR correction. Box plots display the median (centre line), first and third quartiles (box edges) and whiskers extend to 1.5× the interquartile range from the first and third quartiles. All CRISPR screens were performed with a minimum of *n* = 2 technical replicates.

We completed quality-control-passed CRISPR–Cas9 screens on 162 unique organoid cultures from 5 tumour types (85 COAD/READ (exceeding the 63 cell lines screened in DepMap), 59 ESCA, 11 OV, 4 PAAD and 3 STAD; overall 85% success rate), using both MinLibCas9 and Yusa v1.1 libraries for 16 models (Extended Data Fig. 8 for screen quality control and Supplementary Table 4). Using the BAGEL2 method^45^, we identified a median of 1,440 fitness genes in each organoid (range 297–2,160; Fig. 4a and Supplementary Table 5), fewer than reported in cell lines (median = 2,507 for 930 cell lines^9^). The number of fitness genes was inversely proportional to the log fold change (LFC) standard deviation and other quality control metrics (Fig. 4b, Extended Data Fig. 8g,h and Supplementary Tables 4 and 6), indicating that greater variability in gene depletion negatively affects fitness gene identification. We applied rigorous quality control criteria to screens, including replicate concordance, clear discrimination of reference essential versus non-essential genes (area under receiver operating characteristic curve (AUROC) = 0.97 and area under precision-recall curve (AUPR) = 0.973) and good effect size (Fig. 4c,d and Supplementary Table 4), with results comparable to cell lines^3,9^ (AUROC = 0.92 and AUPR = 0.9). Our results demonstrate the feasibility of constructing organoid dependency maps, with the potential to uncover new biology.

## Organoid core fitness genes

**Extended Data Fig. 9:**
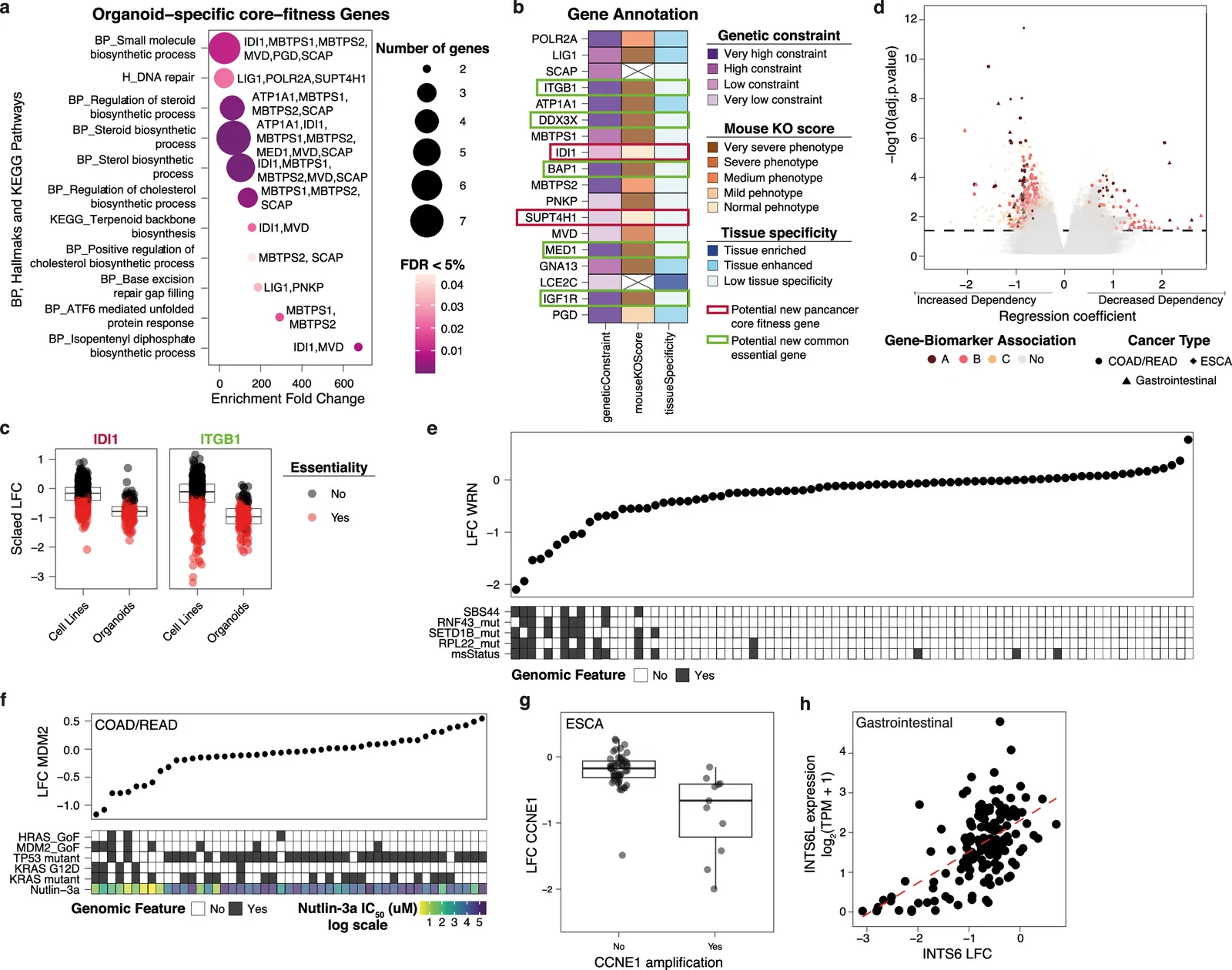
Organoid-specific core fitness genes and gene-biomarker associations. **a**. Pathways enriched in gene set enrichment analysis (GSEA) of 97 organoid-specific core fitness genes. Essential, non-essential and genes untested in organoids owing to single guide RNA (sgRNA) selection were excluded, and compared with pan-cancer core fitness genes from cell lines. Dot size indicates the number of genes per pathway (gene names displayed), while colour represents the FDR-adjusted p-value after Fisher’s exact test. Only pathways with FDR < 10% are shown. BP: biological processes; KEGG: KEGG pathways; H: hallmarks. **b**. Annotation of organoid-specific core essential genes based on genetic constraint, phenotypic effect in mouse knock-outs and tissue specificity of gene expression. Light green indicates highly constrained with severe knockout phenotypes (new core fitness genes); red indicates low constraint with normal knockout phenotypes (cancer organoid selective core fitness genes). **c**. Scaled log fold change (LFC) values of example genes per category in cell lines (n = 930) and in organoids (n = 154). **d**. Volcano plot of statistically significant gene-biomarker associations. Dot colour indicates association significance (A > B > C), while shape corresponds to cancer type. Dashed lines indicate p-value cutoffs. **e**. Log fold change (LFC) profile of WRN with corresponding biomarker associations in colorectal adenocarcinoma (COAD/READ) organoid (n = 85). **f**. Same as panel e for MDM2. Only organoids with Nutlin-3a log IC_50_ values are shown (n = 49). **g**. Differences in CCNE1 LFCs based on the presence or absence of *CCNE1* amplification in oesophageal adenocarcinoma (ESCA) organoids (n = 59). **h**. Correlation between INTS6 LFCs and *INTS6L* expression in all gastrointestinal organoids (n = 151). P-values and effect sizes from the linear regression model are provided in the text. All box plots display the median, first and third quartiles (boxes), and whiskers are 1.5x the interquartile range from the first and third quartiles.

We applied the Adaptive Daisy Model (ADaM) to define core fitness genes in organoids, and compared these with those in cell lines^9^. This identified 654 shared core fitness genes and 97 that were unique to organoids (Supplementary Table 4). Gene set enrichment analysis of organoid-specific core fitness genes highlighted strong associations with steroid biosynthesis (most significant: Biological Process—steroid biosynthetic process, *P* value = 1.38 × 10^−6^; most enriched: Biological Process—isopentenyl diphosphate biosynthetic process, enrichment fold change = 667.5, Fisher’s exact test), potentially linked with a role for cholesterol in regulating intestinal stem cells and tumourgenesis^46^ (Extended Data Fig. 9a and Supplementary Table 4).

Genetic constraint, derived from population-scale human genome sequencing, reflects the degree to which a gene is intolerant to heterozygous variation and provides an orthogonal method to identify disease-linked or functionally important (essential) genes^47,48^. Core fitness genes experimentally derived from CRISPR–Cas9 screens in cell lines^9^ and organoids (this study) were enriched for genetically constrained genes (high categories), as were reference lists of essential genes (Fig. 4e). By additionally excluding genes commonly essential in cell lines (area under the curve (AUC) method), we refined our list to include 18 of 97 organoid-specific core fitness genes and annotated them with mouse knockout phenotypes and GTEx tissue-specific expression (Extended Data Fig. 9b). Genes with low constraint and normal mouse knockout phenotype, such as isopentenyl diphosphate delta isomerase 1 (*IDI1*), required for isoprenoid synthesis and involved in cholesterol production, may reflect cancer organoid selective fitness dependencies. Conversely, genes under high genetic constraint and with severe knockout phenotypes represent candidate core fitness genes identified in organoids—for example, *ITGB1* (Extended Data Fig. 9b,c). Together, this integrative approach combining experimental data from organoids with genetic constraint from human populations identifies core fitness genes.

## Genomic and clinical dependencies

The number of gastrointestinal tract (COAD/READ, ESCA, PAAD and STAD, *n* = 151) and specifically COAD/READ (*n* = 85) and ESCA (*n* = 59) organoids screened with CRISPR, and their genomic deep characterization, enabled analyses to interpret differential gene dependencies. To focus on context-specific effects, we excluded core fitness genes, curated essential and non-essential genes, non-expressed genes, and genes whose depletion was restricted to or absent from a single organoid. For the remaining genes, we performed differential dependency analysis comparing LFC values between organoids where a gene was essential or non-essential based on BAGEL2 scores (minimum of two organoids per group). This approach identified 7,086, 5,103 and 4,082 differentially dependent genes in gastrointestinal, COAD/READ and ESCA organoids, respectively (Fisher’s exact test, adjusted *P* value < 0.05; Supplementary Table 7).

Annotation by genetic constraint showed a distribution across cancer types, in contrast to essential genes (enriched for high constraint) and non-essential genes (enriched for low constraint) (Fig. 4e). However, within these differential dependencies, genes with higher genetic constraint tended towards stronger fitness effects (lower mean LFC), as illustrated in gastrointestinal organoids (Fig. 4f). These observations reveal a broad landscape of differential gene dependencies in organoids and constrained genes tend to show greater dependency penetrance across organoids.

To gain insights into the underlying mechanisms of these differential dependencies, we used linear regression models to link gene fitness effects with our curated genomic, patient and clinical features. From this analysis, we identified 1,841 significant gene–biomarker associations (based on false discovery rate (FDR)-adjusted *P* values and effect size; Extended Data Fig. 9d and Supplementary Table 8), distributed across categories of genetic constraint (Fig. 4f, labelled A, B or C depending on strength of statistical association). In COAD/READ, we observed dependency on *WRN* associated with a *RNF43* mutation (effect size = −0.747, adjusted *P* value = 0.01), *RPL22* or *SETD1B* mutations (effect size = −0.641, adjusted *P* value = 0.027) and mutational signature SBS44 (effect size = −0.823, adjusted *P* value = 0.0009), consistent with MSI status due to deficient DNA mismatch repair (Extended Data Fig. 9e) and showcasing the value of integrating multi-omics biomarkers. Notably, four MSI organoids were not *WRN*-dependent, and lacked expanded TA dinucleotide repeats associated with deficient DNA mismatch repair and underlying *WRN* dependency and inhibitor sensitivity^49^, showing the utility of a large biobank to sub-stratify responses.

We also identified MDM2 dependency for *TP53*-wild-type (effect size = 0.463, adjusted *P* value = 0.001) and unexpectedly for *KRAS*^*G12D*^ COAD/READ organoids (effect size = −0.576, adjusted *P* value = 0.01), of which 4 out of 5 were *TP53*-wild-type. *HRAS* gain-of-function events (effect size = −0.829, adjusted *P* value = 0.0019) partially mirrored *MDM2* gain-of-function events (effect size = −0.566, adjusted *P* value = 0.0039), and all of them correlating with sensitivity to nutlin-3a (an MDM2 inhibitor, PCC = 0.49) (Extended Data Fig. 9f). For ESCA, *CCNE1* amplification, which occurs in 7% of patients^50^, was associated with dependency on CCNE1 (effect size = −0.645, adjusted *P* value = 3.65 × 10^−5^) (Extended Data Fig. 9g). Across gastrointestinal organoids, *INTS6L* expression correlates with *INTS6* dependency (effect size = 2.229, adjusted *P* value = 5.28 × 10^−15^) (Extended Data Fig. 9h), as previously described in cell lines^51^. This rich landscape of dependency–biomarker associations illustrates the value of integrating genomic, transcriptomic and clinical features, and highlights novel hypotheses for functional investigation. Some biomarker-linked differential dependencies map to genes under low genetic constraint (Fig. 4f), which may render them suitable candidate therapeutic targets.

## EGFR dependency varies by *KRAS* allele

**Fig. 5:**
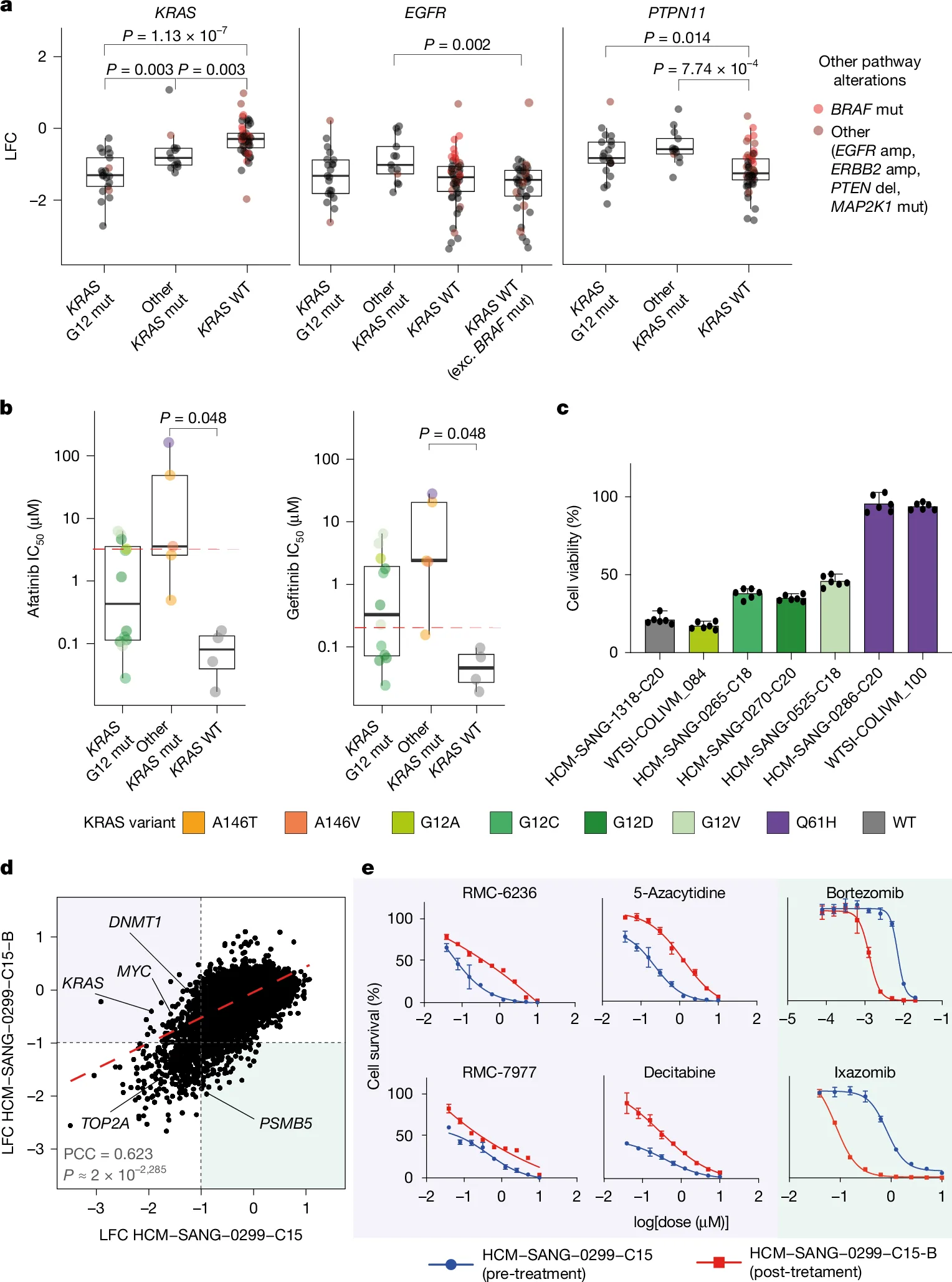
EGFR–RAS–MAPK pathway and pre- and post-treatment specific dependencies. **a**, CRISPR–Cas9 gene knockout dependency values (LFCs) for *KRAS*, *EGFR* and *PTPN11* for COAD/READ (*n* = 85) organoids. WT, wild type. **b**, IC_50_ values for afatinib and gefitinib in COAD/READ organoids (*n* = 21). The dashed red line indicates the maximum drug concentration tested. A two-sided Wilcoxon rank-sum test was used with pairwise comparisons and FDR correction to compare *KRAS* G12 mutants, other *KRAS* mutants and *KRAS* wild-type organoids in **a**,**b**. **c**, Cell viability after removal of EGF from the culture medium. Data are mean ± s.d. of *n* = 6 technical replicates. Experiments were performed in biological duplicates. **d**, Differential CRISPR–Cas9 gene knockout dependency values from two ESCA organoids derived from the same individual: one pre-chemotherapy organoid and another derived from tumour tissue after relapse (pair 1 in Extended Data Fig. 3a). Gene effects are colour-coded based on whether a dependency was observed in the pre- (mauve) or post-treatment (green) organoid. **e**, Differential sensitivity of the same two ESCA organoids as in **d** to two pan-RAS inhibitors (RMC-6236 and RMC-7977, mauve background), two DNMT1 inhibitors (5-azacytidine and decitabine, mauve background), and two PSMB5 inhibitors (bortezomib and ixazomib, green background). Data are mean ± s.d. of three technical replicates. Experiments were performed in biological duplicates. Box plots display the median (centre line), first and third quartiles (box edges) and whiskers extend to 1.5× the interquartile range from the first and third quartiles.

The EGFR–RAS–MAPK signalling pathway is frequently dysregulated in COAD/READ and is targeted by US Food and Drug Administration (FDA)-approved inhibitors of EGFR and KRAS, with an expanding pipeline of RAS agents in development that differ in variant allele specificity, isoform selectivity and mechanism of action^52^. Yet responses remain variable, highlighting the need to define the biological contexts in which specific KRAS inhibitors will confer the greatest therapeutic benefit. Our biobank provides a platform to tackle these questions. In our cohort of 85 COAD/READ organoids with CRISPR data, dependency on this pathway was heterogeneous. *KRAS*-mutant organoids had significantly greater dependency on KRAS than *KRAS* wild-type organoids, whereas *KRAS* wild-type models had greater dependency on EGFR and PTPN11, influenced by alterations in other pathway components such as BRAF (Fig. 5a). Notably, KRAS dependency varied by allele, with G12X variant organoids (in which X denotes any amino acid substition) having the greatest dependency (Fig. 5a). Compared with other *KRAS*-mutant organoids, G12X models also showed increased EGFR and PTPN11 dependency.

**Extended Data Fig. 10:**
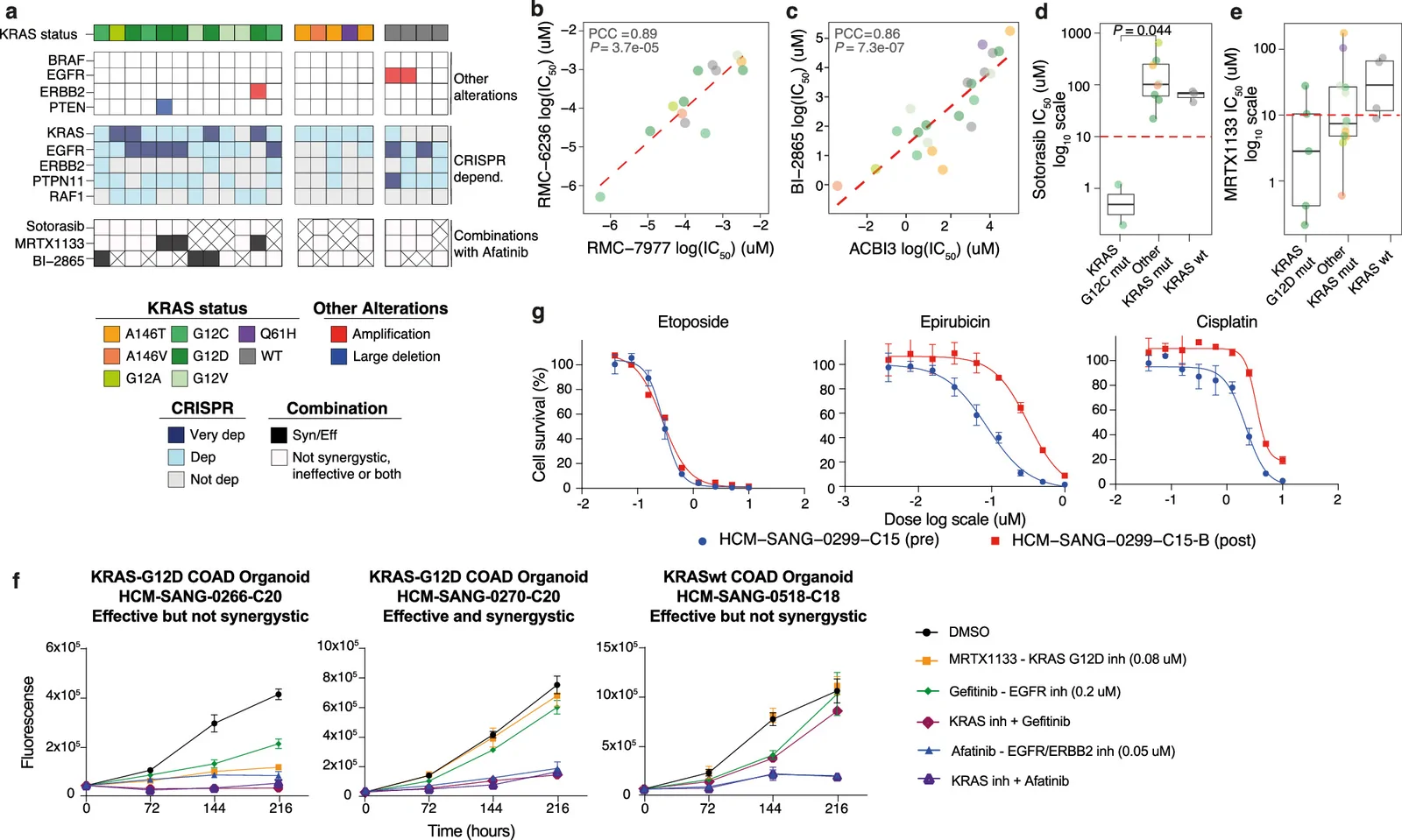
CRISPR-Cas9 and drug sensitivity data. **a**. Heatmap summarising genomic alterations, CRISPR dependencies, and synergy/effect to inhibitors targeting the EGFR–RAS–MAPK pathway of COAD/READ organoids (n = 21). **b**. Correlation between the IC_50_ values of two pan-RAS(ON) inhibitors—RMC-7977 and RMC-6236—in a subset of colorectal adenocarcinoma (COAD/READ) organoids (n = 13). **c**. Correlation between the log(IC_50_) values of a KRAS degrader (ACBI3) and a pan-KRAS(OFF) inhibitor (BI-2865) in a subset of COAD/READ organoids (n = 21). Pearson correlation coefficients (PCCs) and *p*-values were calculated for both b and c. **d**. Differences in IC_50_ values plotted in log_10_ scale for Sotorasib (n = 13), and **e**. MRTX1133 (n = 21) in COAD/READ organoids. Dashed line indicates maximum drug concentration tested. A two-sided Wilcoxon rank-sum test was used, with pairwise comparisons and FDR correction, to compare *KRAS* mutants with the specific variant targeted by the inhibitor, other *KRAS* mutants, and *KRAS* wild-type organoids. Box plots display the median, first and third quartiles (boxes), and whiskers are 1.5x the interquartile range from the first and third quartiles. **f**. Differential sensitivity of two *KRAS* G12D and one *KRAS* wild-type organoid to the KRAS allele-specific inhibitor MRTX-1133 or to EGFR/ERBB2 inhibitors (gefitinib and afatinib), either alone or in combination. Data were collected at days 3, 6, and 9 post-treatment (x-axis), with fluorescence intensity on the y-axis used as a proxy for cell survival. **g**. Differential sensitivity of the same two oesophageal adenocarcinoma (ESCA) organoids as in Fig. 5e to a TOP2A inhibitor (etoposide control) and the treatment received by the patient (epirubicin and cisplatin). Error bars in panels f and g represent the mean ± standard deviation of three technical replicates; experiments were performed in biological duplicates.

To further investigate EGFR–RAS–MAPK pathway dependencies, we profiled drug sensitivity in a subset of *KRAS*-mutant (*n* = 17) and *KRAS* wild-type (*n* = 4) colorectal organoids, selected to represent diverse *KRAS* alleles and dependency profiles (Extended Data Fig. 10a and Supplementary Table 9). Given the diversity of the cohort, we tested pan-RAS, KRAS-specific and variant-specific inhibitors, as well as an EGFR inhibitor and a dual EGFR–ERBB2 inhibitor. All organoids were highly sensitive to the pan-RAS(ON) inhibitors RMC-6236 (mean half-maximal inhibitory concentration (IC_50_) = 0.042 μM) and RMC-7977 (mean IC_50_ = 0.023 μM) (PCC = 0.89 for organoid IC_50_ values; Extended Data Fig. 10b). By contrast, the activities of the pan-KRAS(OFF) inhibitor BI-2865 and the KRAS degrader ACBI3 were similar, but showed overall lower potency (mean IC_50_ ≥ 10 μM for both; PCC = 0.86 for organoid IC_50_ values; Extended Data Fig. 10c). The G12C allele-specific inhibitor sotorasib selectively targeted *KRAS*^*G12C*^ organoids (*P* value = 0.044; Extended Data Fig. 10d), whereas MRTX1133 (G12D allele-selective) inhibited G12D but also affected non-G12D organoids (Extended Data Fig. 10e). Overall, we observe variable sensitivity to RAS inhibitors with different modes of action.

Organoids with high CRISPR-derived EGFR dependency were amongst the most sensitive to the EGFR inhibitors afatinib and gefitinib. Notably, *KRAS* wild-type and *KRAS*^*G12X*^ organoids had greater sensitivity compared to *KRAS* non-G12 variants (Fig. 5b). Upon combined EGFR and KRAS inhibition, only a subset of *KRAS*^*G12X*^-mutant organoids exhibited pronounced loss of viability (combined maximal effect (*E*_max_) > 85%) and synergy (Bliss > 0.25), including when using the pan-KRAS(OFF) inhibitor BI-2865 (Syn/Eff in Extended Data Fig. 10a,f), in part owing to potent monotherapy activities. Finally, a *KRAS*^*Q61H*^ variant organoid was unresponsive to EGFR inhibition by either CRISPR or small molecules (Fig. 5b). We further confirmed this by showing that EGF withdrawal from the culture medium did not reduce viability in Q61H organoids, unlike in G12X or wild-type organoids (Fig. 5c). Collectively, the biobank and dependency map yield clinically relevant functional insights into the EGFR–KRAS–MAPK pathway, revealing substantial heterogeneity in responses to RAS and EGFR inhibition and showing that EGFR dependency varies across organoids with distinct *KRAS* variant alleles^53^.

## Dependencies in patient-matched organoids

Derivation and interrogation of patient-matched samples pre- and post-treatment can inform on tumour evolution, mechanisms of resistance and approaches to target treatment-refractory cells. Patients with ESCA have a poor prognosis and few treatment options, particularly in advanced disease. We therefore compared the CRISPR–Cas9 dependency profiles for paired ESCA organoids from a 69-year-old man with stage III disease derived from a laparoscopic biopsy (HCM-SANG-0299-C15) before chemotherapy (epirubicin plus capecitabine plus cisplatin) and following surgical resection at the time of relapse (HCM-SANG-0299-C15-B). Both organoids retained a *KRAS* amplification, whereas a *MYC* amplification in the pre-treatment was absent in the post-treatment organoid (Extended Data Fig. 3b), implying clonal evolution under the selective pressure of chemotherapy. Although the overall dependency profiles of the matched organoids were similar, MYC dependency was reduced in the post-treatment organoid, consistent with loss of *MYC* amplification (Fig. 5d).

Curation of differential dependencies post-treatment identified several druggable targets, including a reduced dependency on KRAS and DNMT1 and increased dependency on the proteasome core subunit PSMB5 (Fig. 5d), which we validated experimentally. The post-treatment organoid had reduced sensitivity to two pan-RAS inhibitors (RMC-6236 and RMC-7977) and two DNMT1 inhibitors (5-azacytidine and decitabine), and increased sensitivity to two proteosome inhibitors (bortezomib and ixazomib), consistent with predictions from CRISPR experiments (Fig. 5e). There was no difference in *TOP2A* dependency (Fig. 5d) or sensitivity to the TOP2A inhibitor etoposide in the matched samples (Extended Data Fig. 10g), indicating that differential effects are likely to be target-specific. The organoid derived from the pre-treatment sample also exhibited increased sensitivity to epirubicin and cisplatin versus the post-treatment sample, aligning with clinical relapse (Extended Data Fig. 10g). Our biobank captured the functional impact of treatment driven tumour evolution, selecting for cancer driver events and conferring differential dependencies that can be unmasked through CRISPR screening and that are candidates for drug repositioning.

## Discussion

Reductionist models are central to cancer biology and drug discovery. However, cancer heterogeneity strongly influences prognosis and treatment response, creating a need for larger, more representative model collections to advance precision medicine. Our comprehensive, highly annotated biobank complements 2D cell lines and is designed to catalyse discovery. To maximize access, models are deposited in public repositories and datasets are available via the Cell Model Passport^40^ and DepMap Miner (https://dataminer.depmap.sanger.ac.uk/) portals.

This study is distinctive for the number of highly annotated organoids and the open availability of detailed genomic, patient and clinical data for each organoid^10^. Future biobanking would benefit from deeper tumour annotation, including histology, single-cell and spatial transcriptomics, and matched stromal and immune cultures^54^. We used tissue-specific, standardized conditions to generate a molecularly diverse biobank and enable cross-model comparisons. Tailored culture conditions may enrich specific molecular subtypes and alter gene dependencies^55^, and xenotransplantation provides an in vivo context to assess microenvironmental effects.

Genome-wide CRISPR screens in tumour organoids are rare^42,56^, yet we map dependencies across a diverse biobank and achieved cell line-level quality despite the added complexity of 3D culture. Distinguishing cancer-selective dependencies from core fitness genes may inform therapeutic index, although matched normal cells are rarely available. To strengthen interpretation, we integrated population-scale genetic constraint data^48^, providing orthogonal support for core fitness genes and indirect evidence of target tolerability.

Our organoid dependency map identified novel and clinically relevant vulnerabilities. We found allele-specific differences in KRAS and EGFR dependencies, with *KRAS*^*G12X*^ variants showing greater reliance on KRAS and upstream EGFR signalling. This supports a model^53,57^ in which G12X tumours remain responsive to upstream receptor activation, unlike signalling-independent Q61X variants, with therapeutic implications for targeting *KRAS*^*G12X*^ cancers, and has been proposed to underlie the enrichment of *KRAS*^*Q61X*^ mutations as secondary resistance events to EGFR inhibitors in colorectal cancer. Additionally, by generating and dependency mapping patient-matched organoids before and after treatment, we traced tumour evolution under therapeutic pressure and uncovered new hypotheses for intervention in treatment-refractory ESCA. In summary, we provide a preview of a cancer dependency map across a diverse cohort of highly annotated organoid cultures, with potential to enhance and extend existing efforts as a tool for discovery.

Large-scale sequencing efforts^58,59^ defined tumour mutational landscapes and transformed understanding of cancer biology. A next frontier in precision oncology is to move beyond descriptive genomics towards multi-scale, integrative functional maps that enable mechanistic modelling of disease, including virtual cell frameworks^60,61^. This will require systematic modelling and perturbation across representative tumour cohorts integrated with patient data. Our organoid biobank and dependency map mark a major step towards building functionally informed maps of human cancer.

## Methods

### Ethical approval and sample collection

All necessary ethical approvals to conduct this work and to ensure that the derived organoids could be used for academic and commercial purposes were obtained from each participating clinical site for the collection of patient samples as well as for the Wellcome Sanger Institute for organoid derivation (IRAS ID:203519; REC 16/LO/1110). Further details are provided in Supplementary Information.

### Availability of Organoid Biobank

Organoids have ethical approval for use in academic and commercial purposes. Models are being distributed by American Type Tissue Culture (ATCC) as part of the HCMI collection (https://www.atcc.org/hcmi) and EMD Millipore. Repository details and model availability are provided in Supplementary Table 2.

### Organoid derivation

Detailed protocols for organoid derivation, cryopreservation and routine culturing are published on protocols.io and include embedded demonstration videos and workflow diagrams^62–64^. Tumour samples were washed 3 times with PBS, minced, and either cryopreserved after centrifugation (800*g*, 2 min)^63^ or enzymatically digested for 1–2 h at 37 °C. The suspension was filtered (100 μm), centrifuged and washed to remove debris and digestion buffer^62^. Isolated cells were embedded in approximately 15 μl droplets of extracellular matrix (80:20 basement membrane extract (BME):medium; 6.4–9.6 mg ml^−1^ protein; Cultrex BME Type 2 Select 3532-001-02) and plated in pre-warmed 6-well plates following established protocols^64^. After polymerization (15–20 min, 37 °C), 2 ml of organoid medium prepared using established recipes^39,65–67^ was added, supplemented with antibiotics and 1 μl ml^−1^ ROCK inhibitor (Y-27632, Staratech Scientific S1049-SEL-5mg).

After expansion to ≥25 million cells, 25 cryovials were banked and pellets collected for sequencing. Post-thaw viability was confirmed by re-culturing for four passages with freeze–thaw quality control assessment.

### Organoid culture

Organoids were maintained either in 80% BME-2 droplets or in 5% BME-2 suspension culture^38^. In the 80% BME-2 culture, organoids were cultured as described above. For the 5% suspension method, cancer organoids were suspended in a medium/extracellular matrix (ECM) dilution. For example, combining 10 ml of medium with 500 μl of BME-2 achieved the desired concentration, and organoids were then cultured in ultra-low-adherent flasks or plates.

For passaging, the medium, cells and ECM were collected and centrifuged at 800*g* for 2 min. After discarding the supernatant, organoids were dissociated using TrypLE (Gibco 12604013), an enzymatic reagent. The suspension was incubated for 10–60 min, allowing dissociation into small clumps. The cells were then pelleted and replated as described^64^.

### Model identifiers

All models presented in this study have a sanger_ID starting with ‘WTSI’, corresponding to the Wellcome Trust Sanger Institute. Additionally, if a model is shared with the HCMI, it also has an HCMI ID starting with ‘HCM’. When both IDs are available, the HCMI nomenclature is used as the sample_ID. The equivalences are shown in Supplementary Table 2.

### Whole-genome sequencing and analysis

#### Sequencing and alignment

DNA extracted from snap-frozen tumour tissues, snap-frozen organoid cell pellets, blood or formalin-fixed paraffin-embedded normal tissues samples were prepared for WGS. Whole genome paired-end sequencing reads (150 bp) were generated using the Illumina HiSeq X Ten platform with an average coverage of 38×, comparable to coverage used for PCAWG. Reads were aligned using the BWA-MEM (v0.7.17) tool^68^. PCR duplicates, unmapped and non-uniquely mapped reads were filtered out before downstream analysis.

#### SNV and indel calling

Somatic SNVs and short indels were identified using cgpCaVEMan^69^ and cgpPindel^70^, respectively. Germline variants and artefacts were filtered using matched normal samples and a panel of normals, with further post-processing using cgpCaVEManPostProcessing (https://github.com/cancerit). Variant allele frequencies (VAFs) were estimated using vafCorrect (v2.4.0)^71^, and variants with VAF > 0.05 were retained. Further details in Supplementary Information.

#### Structural variant and copy number calling

Copy number and allele-specific information were derived using AMBER (v3.5)^72^ and COBALT (v1.11)^72^. Somatic and germline SNVs and indels were identified using SAGE (v2.8) and SnpEff (v5.0)^73^. Somatic SVs were called using GRIDSS2 (v2.12.0)^74^, annotated with RepeatMasker (v4.1.2)^75^ and kraken2 (v2.1.2)^76^, and filtered with GRIPSS (v1.9)^77^. Subsequently, this information was integrated to calculate the microsatellite status, tumour purity, ploidy, WGD and SCNAs using PURPLE (v2.54)^72^. SCNAs were converted into discrete copy number states. SAGE, GRIPSS, AMBER, COBALT and PURPLE were developed by the Hartwig Medical Foundation (HMF) (https://github.com/hartwigmedical/hmftools). Further details in Supplementary Information.

#### Cancer driver event annotation

We compiled a list of 783 cancer driver genes, derived as the union of two complementary gene sets from the IntOGen^78^ and COSMIC^79^ databases, as previously reported^9^. Each gene was annotated by its mechanism of action as either activating (Act, for oncogenes), loss-of-function (LoF, for tumour suppressor genes), ambiguous (with evidence of both mechanisms), or fusion. The complete list of cancer driver genes is in https://cellmodelpassports.sanger.ac.uk/downloads.

We collated individual putative cancer driver mutations (for example, frameshift, nonsense, stop-lost, exonic splicing silencer, missense or in-frame mutations) among SNVs and indels within these cancer driver genes from four data sources: IntOGen (including Cancer Genome Interpreter and BoostDM)^78^, MSKCC^80^, and cancer predisposition variants, that were identified by overlap with a reference set of pathogenic germline variants with matching effect^81^.

#### Loss of heterozygosity

We considered a tumour suppressor gene or an ambiguous gene to have loss of heterozygosity if the DNA copy number (CN) of the minor allele was <0.5 and the difference between the round ploidy and the round total CN was >0 (there was no amplification in the non-mutated allele) or if the VAF of a loss-of-function mutation was >0.85.

#### Biallelic alterations

Biallelic alterations included all cases with loss of heterozygosity, as well as homozygous deletions, SV disruptions or instances where each allele was affected by a different loss-of-function mutation.

#### Multiplicity, CCF and clonal mutations

SNVs were intersected with segment SCNAs using the GRanges and findOverlaps functions from the GenomicRanges R package (v1.56.1) to obtain information on the major allele, minor allele, and total tumour CN for each SNV, along with the VAF and tumour purity. The multiplicity (the number of chromosomal copies harbouring a given mutation) and CCF (the proportion of cancer cells carrying a specific mutation within a tumour sample) were then calculated according to the formulas in Steele et al.^82^ and Dentro et al.^83^. Clonal mutations, defined as genetic alterations present in all cancer cells within a tumour, were identified as those with a CCF > 0.75.

#### CN correlation between paired organoids and tumours

SCNA segment data was divided into 100 kb bins across the genome, and the CN for each segment was calculated as the mean CN of all positions within the 100,000 bp window. Positions listed in the ENCODE blacklist^84^ (https://github.com/Boyle-Lab/Blacklist) were excluded from this analysis. Pearson correlations were then computed using the mean CN for each segment between paired organoid and tumour samples.

#### Focal amplifications

Focal amplifications were defined as the presence of at least two genomic segments, each exceeding 100 kb in size, with a log_2_(CN/ploidy) value > 6.

#### Signature analysis

Mutational signatures were extracted using SigProfilerExtractor (v1.2.2)^85^ for the tumour and organoid samples from each tumour type separately. COAD/READ-MSI samples were analysed separately from COAD/READ-MSS samples. STAD samples were not analysed due to insufficient sample size. The resulting matrix file was used as an input for SigprofilerAssignment (v0.2.5)^30^ to assign known COSMIC v3.4 signatures within single base substitution (SBS). De novo signatures were further refitted using the COMICv3.4 and an additional set of novel CRC and MSI specific signatures identified and validated in Mutographs project^86^.

For data visualization, COSMIC mutational signatures representing less than 10% of mutations within each sample were categorized as ‘Others’ and excluded from calculations of both the proportion of models with that mutational signature and the proportion of mutations representing each signature by cancer type. SBS5 and SBS40a were collapsed into a single category, based on the hypothesis that these signatures arise from a combination of correlated mutational processes^87,88^.

#### Homologous recombination deficiency

Homologous recombination deficiency was assessed using CHORD (v2.0.3)^34^.

#### Complex genomic rearrangements

Chromothripsis and other complex genomic rearrangements were detected using ShatterSeek (v1.1)^26^, as previously described^89^.

### RNA-seq and analysis

Paired-end transcriptome reads (75 bp) were quality filtered and mapped to GRCh38 (ensemble build 98) using STAR (v2.5.0c)^90^ with a standard set of parameters (https://github.com/cancerit/cgpRna). Resulting bam files were processed to get the per gene read count and transcripts per million (TPM) data using RSEM (v1.3.3)^91^. TPM values were used for the downstream analysis.

#### CMS/CRIS subtypes

Consensus molecular subtypes (CMS) and colorectal cancer intrinsic subtypes (CRIS) subtypes were inferred for COAD/READ organoids with CMSCaller^36,92^ using RSEM expected count data.

### CRISPR–Cas9 screening

Detailed protocols including process diagrams and example data for generating Cas9-expressing organoid cultures and CRISPR–Cas9 library transduction in organoid are published on protocols.io^93,94^. Stable Cas9-expressing organoids were generated using lentiCas9-Blast (Addgene 52962) with polybrene (8 μg ml^−1^). Following overnight incubation, medium was replaced with complete medium containing Y-27632 (2.5 μM), and blasticidin (Invivogen, ant-bl-1, 10 mg ml^−1^) selection was applied 120 h after transduction. Cas9 activity was measured using a fluorescent reporter assay (Addgene 67982 and 67981)^95^. Only organoid lines with activity of 75% or more were selected for single guide RNA (sgRNA) library transduction.

Two genome-wide CRISPR–Cas9 sgRNA libraries were used. The Human CRISPR Library Yusa v.1.1 (ref. ^3^), containing 100,086 sgRNAs that target 18,009 genes (with 5–10 sgRNAs per gene) and 1,004 non-targeting sgRNAs; and the Minimal Genome-Wide Human CRISPR–Cas9 Library (MinLibCas9, Addgene 164896)^41^, which includes a selected sgRNA primarily drawn from Yusa v1.1 comprising 37,522 sgRNAs that target 18,761 genes (with 2 optimal sgRNAs per gene) and 200 non-targeting sgRNAs (Extended Data Fig. 8a). Lentiviral volume required for multiplicity of infection of 0.3 was determined by titration, and transduction efficiency assessed by BFP flow cytometry.

A total of 12.5 × 10^8^ (Yusa v1.1) or 3.3 × 10^7^ (MinLibCas9) cells were transduced in triplicate using the same batch of Cas9-transduced organoids. No significant batch effects were observed across independent batches of Cas9-expressing organoid lines (Extended Data Fig. 8b). Cells were infected with the lentiviral-packaged whole-genome sgRNA library (for 100× coverage), in medium containing polybrene (8 μg ml^−1^), and Y-27632 (2.5 μM). Following overnight incubation, cells were plated in fresh medium in a 5% suspension. Transduction efficiency (target of 30%) was confirmed on day 6. Screens were maintained under puromycin selection (Invivogen, ant-pr-1, 10 mg ml^−1^) for a further 16 days (21 days in screen in total). A final selection efficiency of at least 60% was required for the screen to pass. At the end of the screen approximately 2.5 × 10^7^ cells were collected, pelleted, and stored at −80 °C for downstream processing.

### CRISPR screen data processing

CRISPR screens performed using the Yusa v1.1 (ref. ^3^) and MinLibCas9 (ref. ^41^) libraries were harmonized by restricting analyses to shared sgRNAs, enabling integrated downstream processing. Quality control procedures, adapted from established cell line screening pipelines^3^, were applied to assess replicate concordance, sgRNA representation and classifier performance in distinguishing essential from non-essential genes. Low-quality replicates and organoids failing predefined quality control criteria were excluded prior to further analysis. Read counts were normalized and corrected for copy-number effects using CRISPRcleanR (v3.0.1)^96^, followed by batch correction across libraries^97^. Gene-level fitness effects were estimated using BAGEL (v2)^45^ with curated reference gene sets, and LFC values were scaled relative to essential and non-essential controls to facilitate cross-organoid comparability. Full details of sgRNA selection, quality control thresholds, normalization, batch correction, statistical procedures and final selection of models are provided in the Supplementary Information.

### CRISPR screen processed data analysis

#### Analysis of organoid core fitness genes

We applied ADaM implemented in the CoRe R package (v1.0.0) (https://github.com/DepMap-Analytics/CoRe)^98^ using the same curated reference essential gene set as previously mentioned^3^ to calculate false-positive rates. This analysis identified 751 core essential genes (Supplementary table 4). Although we used all organoids together as input, only three cancer types were included (PAAD and STAD organoids were excluded), so the resulting core fitness genes do not represent a pan-cancer set.

We compared core fitness genes in organoids with those identified in cell lines^9^ and observed an overlap of 654 genes, with 97 genes identified as organoid-specific (Extended Data Fig. 9). We performed a Fisher’s exact test on these organoid-specific core fitness genes to perform pathway enrichment analysis, using Gene Ontology Biological Processes, KEGG pathways^99^ and Hallmarks^100^ obtained from the msigdbr R package (v7.5.1)^101^. For this analysis, PanCancer common essential genes identified using the AUC method were excluded^98^. For visualization, when multiple pathways shared the same set of organoid-specific core fitness genes, only the pathway with the most significant *P* value was displayed.

#### Differential dependency analysis in all gastrointestinal, COAD/READ and ESCA organoids

We conducted an analysis to identify genes with the greatest dependency variability across organoids within each cancer type and to highlight context-specific dependencies. The analysis proceeded as follows:

1. Gene filtering based on consistent depletion/non-depletion. Using binary dependency matrices generated by BAGEL2 for each cancer type, we excluded genes that were only considered depleted in a single organoid and not depleted in only one organoid.
2. Exclusion of core fitness and control genes. Genes previously classified as pan-cancer core fitness genes in cell line datasets, the 751 organoid-specific core fitness genes identified in this study, as well as control sets of essential and non-essential genes, were removed to ensure a focus on genes with differential dependency profiles.
3. Expression thresholding. Genes with a mean expression level of log_2_(TPM + 1) < 0.1 in each cancer type (considered ‘not expressed’) were also excluded from the analysis.

Following these filtering steps, for each remaining gene, we calculated the difference in LFCs (delta LFC) between organoids where the gene was depleted and those where it was not and tested the statistical significance with a Fisher’s exact test. Only genes with a FDR-adjusted *P* value < 0.05 were considered differentially dependent (7,086 for gastrointestinal, 5,103 for COAD/READ and 4,082 for ESCA; Supplementary Table 7).

### Biomarker analysis

To identify molecular and clinical features associated with context-specific gene dependencies, we performed a systematic biomarker analysis across gastrointestinal, COAD/READ and ESCA organoids. Candidate dependencies were selected from the differential dependency analysis based on recurrence criteria and evaluated against a curated set of genomic, transcriptomic and clinical features, including driver mutations and specific variants, copy number alterations, structural events, mutational signatures, gene expression, pathway activity scores, and composite loss- and gain-of-function events. Associations between gene fitness effects and features were tested using linear regression models incorporating relevant technical and biological covariates, with significance assessed using likelihood-ratio tests and multiple testing correction. Significant associations were prioritized based on adjusted *P* value and effect size and classified into tiers reflecting strength of association. Full details of feature selection, model specification, statistical thresholds and classification criteria are provided in the Supplementary Information.

### High-throughput drug screens

For high-throughput screening, organoids were dissociated into single cells, counted and seeded in 5% BME-2 suspension cultures at model-specific optimized densities. After 96 h to allow organoid re-formation, assay plates were prepared with a 50% BME-2:organoid medium base layer, and organoids were transferred into 384-well plates using Multidrop Combi (Thermo Scientific) dispensers. Twenty-four hours later, compounds were dispensed using an Echo555 (Labcyte), and cells were treated for 72 h. Viability was measured using CellTiter-Glo 2.0 (Promega).

Two independent high-throughput screening projects were conducted. The first project used a full 7 × 7 concentration matrix (49 measurements) for each drug combination, and the second used a reduced 25 measurement concentration matrix encompassing the same concentration range. Compounds were tested in biological duplicates in the first project and single replicates in the second project, with higher technical replication for agents included in multiple combinations like afatinib (also tested as monotherapies).

Raw viability data were analysed independently for each project. Data were normalized per plate using negative (untreated, DMSO) and positive (MG-132, staurosporine, blank) controls. Dose-response curves were fitted using a non-linear mixed-effects model to estimate IC_50_ and AUC values using the gdscIC50 R package (v1.7.3)^102^. For combination treatments, the maximum combination effect (combo_MaxE) was defined as the second-highest measured inhibition. Bliss excess was calculated as the difference between observed combination inhibition and the predicted Bliss additivity of the corresponding monotherapies. The results presented are the mean values across both projects.

### Validation drug sensitivity testing

For validation drug sensitivity testing, organoids were dissociated into single cells, resuspended in organoid medium, and seeded into 96-well plates over a 50% BME-2:organoid medium base layer (2,000–5,000 cells per well, depending on the model). For monotherapy treatments, nine drug concentrations spanning a 256-fold range were added four days after plating, in technical triplicates. For combination treatments, five concentrations of KRAS inhibitors (sotorasib or MRTX1133) across a 256-fold range were tested with two fixed concentrations of EGFR and ERBB2 inhibitors (afatinib and gefitinib), also in triplicate. Viability was quantified using CellTiter-Glo 2.0 at 72 h post-treatment for monotherapies and at 0, 3, 6 and 9 days for combinations. Dose-response curves were fitted using GraphPad Prism, with three technical and two biological replicates per condition.

### EGF depletion from the medium

Organoids were dissociated into single cells, resuspended in organoid culture medium either supplemented with or deprived of EGF, and seeded into 96-well plates over a 50:50 medium-to-ECM layer. The bottom ECM-containing layer was prepared with the same EGF condition as the overlaid medium. Cell viability was quantified using CellTiter-Glo 2.0 seven days after seeding.

### Reporting summary

Further information on research design is available in the Nature Portfolio Reporting Summary linked to this article.

## Online content

Any methods, additional references, Nature Portfolio reporting summaries, source data, extended data, supplementary information, acknowledgements, peer review information; details of author contributions and competing interests; and statements of data and code availability are available at https://doi.org/10.1038/s41586-026-10830-y.

## Supplementary information

Supplementary Information Supplementary results, Methods and references and a guide for Supplementary Tables 1–10.

Reporting Summary

Supplementary Tables Supplementary Tables 1–10

Peer Review File

## Data Availability

The raw WGS, targeted gene sequencing (TGS) and RNA-seq data from organoids used in this study, along with matched tumour tissues and normal samples, have been deposited in the European genome-phenome archive (EGA): WGS, EGAD00001015469; TGS, EGAD0000101546; RNA-seq, EGAD00001015470. Main results of the analysis are presented in the Supplementary Tables. In addition, genomic, transcriptomic and CRISPR processed data are available in the Cell Model Passports^40^ and DepMap miner database. Additional datasets required to reproduce figures and analyses presented in this Article are available on Figshare (https://doi.org/10.6084/m9.figshare.28339340 (ref. ^103^)). Public datasets used in this study include resources from IntOGen^78^, the COSMIC Cancer Gene Census^79^, the Cancer Genome Interpreter and BoostDM^78^, MSKCC Cancer Hotspots^80^, the Cancer Predisposition Variant dataset^81^, the ENCODE blacklist^84^ and COSMIC mutational signatures v3.4^30^. Additional curated data from large-scale CRISPR screens and cancer genomics studies were obtained from Pacini et al.^9^. An overview of all tables and datasets used in this study, including their location, source and associated analyses, is provided in Supplementary Table 10.

## Code availability

The code used for CRISPR data analysis and reproduction of the five main figures in this study is accessible via https://github.com/Garnett-Lab/SangerOrganoidBiobank. Genomic and transcriptomic data processing was performed using established bioinformatics pipelines and publicly available software tools. Details of the software packages, versions and parameters used are provided in the Methods and Supplementary Information.
