# Supplementary material for: A tumour-derived organoid biobank maps cancer gene dependencies
Source: Nature. 2026 Aug 5;657(8132):765–74. doi: 10.1038/s41586-026-10830-y (PMC13581617; doi:10.1038/s41586-026-10830-y)
Supplement: Supplementary file 1 [file 41586_2026_10830_MOESM1_ESM.pdf]

nature portfolio

https://doi.org/10.1038/s41586-026-10830-y

Supplementary information

A tumour-derived organoid biobank maps cancer gene dependencies

In the format provided by the authors and unedited

Supplementary Results

CMS and CRIS subtypes

RNA sequencing (RNA-seq) of the organoids was performed, enabling us to classify the COAD/READ models into two of the most validated and clinically relevant transcriptional subtypes: consensus molecular subtypes[36] (CMS 1-4) and colorectal cancer intrinsic subtypes (CRIS A-E)[37] (Supplementary Table 3 and Extended Data Fig.6c). Most CMS2 organoids were also classified as CRISC (p value < 0.0001, Fisher's exact test), and showed significant enrichment in *TP53* mutations (CMS2 p value = 0.0011 and CRISC p value = 0.0168, Fisher's exact test) and wild-type *BRAF* or *KRAS* status (CMS2 p value = 0.0063 and CRISC p value < 0.0001, Fisher's exact test). Interestingly, these organoids predominantly originated from distal colon or rectal tumours (CMS2 p value < 0.0001 and CRISC p value < 0.0152, Fisher's exact test). CRISC models had a higher frequency of chromothripsis (p value = 0.0005, Fisher's exact test). Furthermore, nearly all MSI organoids were classified as CRISA (p value < 0.0001, Fisher's exact test), with lower rates of chromothripsis and WGD (chromothripsis p value = 0.0002 and WGD p value < 0.0001, Fisher's exact test). MSS samples within the CRISA group showed an enrichment of *BRAF* or *KRAS* mutations (p value < 0.0001, Fisher's exact test) and were classified as CMS3 (p value < 0.0001, Fisher's exact test). These results suggest that while both CMS and CRIS classifications are valuable for understanding COAD/READ organoid biology, CRIS offered greater precision in correlating genomic features, such as mutation profiles and chromosomal alterations.

Organoid genomic stability over time

To establish a renewable resource, we assessed genomic stability of organoids in long-term culture and through multiple freeze-thaw cycles. Four models (two COAD/READ and two ESCA) were profiled by WGS and RNA-seq at banking and at 1–2-month intervals for up to 6 months (Time 1–5; maximum 204 days; see Supplementary Methods). Overall ploidy was stable, except for an increase in HCM-SANG-0278-C20 from Time 3, reflecting selection of a pre-existing WGD (Extended Data Fig.7a). Mutational load, SVs, and SCNAs also remained stable, with no new driver mutations and somatic mutation concordance >75% across all timepoint comparisons (Extended Data Fig.7b-f). Mutational signatures were remarkably consistent over time with minor variations (Extended Data Fig.7g) and transcriptional profiles were concordant (PCC > 0.75; Extended Data Fig.7h).

Two models (HCM-SANG-0266-C20 and HCM-SANG-0295-C15) were further analysed across 3 or 4 freeze–thaw cycles, showing high SNV/Indel concordance and minimal transcriptomic variation (Extended Data Fig.7i-j). Long term culture caused slightly greater—

but still modest—genetic drift than repeated freeze–thaw cycles. Consistent with prior reports[38,39], organoids largely preserve genomic and transcriptional integrity during extended culturing and storage, confirming their robustness as genetically stable renewable tumour models.

Supplementary Methods

Ethical approval and sample collection

Patient tissue samples used in this study were received from multiple NHS clinical sites:

- University Hospital Birmingham via the Human Biomaterial Resource Centre (HBRC). The participant recruitment process, information sheets and consent forms have previously been given a favourable opinion by North West – Haydock Research Ethics Committee (REC), REC reference number 15/NW/0079, sub-approval project code 17-287.
- University of Cambridge and Cancer Research UK (Cambridge Institute). Clinical data and tissue samples for the patients were collected on the prospective cohort study Cambridge Translational Cancer Research Ovarian Study 04 (CTCR-OV04), with IRAS project ID 4853, and which was approved by the Institutional Ethics Committee (REC reference number 08 /H0306/61).
- Addenbrookes Hospital, Cambridge, via the OCCAMS project. The OCCAMS participant recruitment process, information sheets and consent forms have previously been given a favourable opinion by Cambridge South REC, REC reference number 10/H0305/1.1.
- Application to access patient tissue and organoids was authorised by the NHS Greater Glasgow and Clyde Biorepository under their NHS REC approval with ethical approval granted in biorepository application 22/WS/0020.
- Samples at Imperial College London were collected under the authority of Imperial College Healthcare Tissue and Biobank (HTA licence 12275, REC reference number 22/WA/0214, IRAS reference 312147, project number N19001-5A). All patients gave written informed consent.
- University of Southampton Biobank (HTA licence no. 12009) through the Pancreatic Cancer: Molecular and Genetic Mechanisms project. The participant recruitment process, information sheets and consent forms for this project have previously been given a favourable opinion, REC reference number: 10/H0502/72. The Molecular Pathology of Colorectal Cancer project, REC reference number: 07/H0504/125, the Evolution of abdomino-pelvic cancers under immune selection study, REC reference number: 21/EE/0110 and the Upper Gastro-Intestinal Tumour Ecology study IRAS 233065 Southampton General Hospital provided samples through the OCCAMS project using the same REC approved documentation used by Addenbrooke's Hospital. In addition, where patients undergoing surgery have consented for use of their surplus samples in research, these may also be received from Southampton Biobank.

Collection of clinical data

Study data were collected and managed using REDCap[104] (Research Electronic Data Capture)[1] electronic data capture tools hosted at the Wellcome Sanger Institute. REDCap is a secure web-based software platform designed to support data capture for research studies. The RedCap application was used to securely capture pseudo-anonymised clinical data for each sample, including patient information, tumour and molecular pathology and treatment.

Authentication of models

To prevent cross-contamination or misidentification, all organoid models and their corresponding normal tissue samples were authenticated using a panel of 95 single nucleotide polymorphisms (SNPs) analysed with the Fluidigm 96.96 Dynamic Array IFC system. Organoids were required to match their corresponding normal tissue sample to confirm their authenticity and not match any other donor sample. A match is defined by a **SNP Score** ≥**75%**, calculated as:

SNP Score = (% of matching SNPs) × (% of SNPs called in both samples).

Whole-genome sequencing and analysis

Single nucleotide variant and insertion/deletion calling

Somatic single nucleotide variants (SNVs) were identified using cgpCaVEMan[69] and values for mutant copy number, wild type copy number and normal contamination parameters were supplied from ascatNgs[105] analysis. Short insertions and deletions (Indels) were identified using cgpPindel[70]. Matched blood or FFPE samples were used for filtering sample-specific germline variants. Additional germline variants and technology-specific artefacts were removed by filtering against a panel of 100 unrelated normal samples (ftp://ftp.sanger.ac.uk/pub/cancer/dockstore/human/GRCh38_hla_decoy_ebv/SNV_INDEL_ref_GRCh38_hla_decoy_ebv-fragment.tar.gz). Additional sequencing-specific (TGS or WGS) post-processing filters were applied using in-house tool cgpCaVEManPostProcessing (https://github.com/cancerit). Variants sites flagged as ‘PASS’ were considered for further downstream analysis. Unbiased analysis of mutant and wild-type reads found at the loci of the SNVs and Indels were assessed within a group of related samples using vafCorrect (v2.4.0)[71], which assigns a variant allele frequency (VAF) to each sample within each variant. We applied VAF > 0.05 filter for subsequent analysis.

Structural variant and copy number calling

For each tumour-normal or organoid-normal sample pair, B-allele frequency of heterozygous SNP sites was generated using AMBER (v3.5)[72] and read-depth ratios were determined using COBALT (v1.11)[72]. Somatic and germline SNVs and Indels were identified using SAGE (v2.8), with recommended parameters for 30x tumour sequencing depth, and their effect annotated using SnpEff (v5.0)[73]. Somatic structural variants (SVs) were called using GRIDSS2 (v2.12.0)[74], annotated with RepeatMasker (v4.1.2)[75] and kraken2 (v2.1.2)[76], and filtered with GRIPSS (v1.9)[77]. Subsequently, B-allele frequencies, read-depth ratios, somatic SNV allele frequencies, and somatic SV breakpoints were integrated to calculate the microsatellite status, tumour purity, ploidy, whole genome duplication (WGD) and somatic copy number alterations (SCNAs) (gene and segment level) of each tumour and organoid independently using PURPLE (v2.54)[72]. SAGE, GRIPSS, AMBER, COBALT and PURPLE were developed by the Hartwig Medical Foundation (HMF) and code, tools and extensive documentation are freely available on github: https://github.com/hartwigmedical/hmftools.

SCNA data from the prediction tool was converted into integer copy number (CN) categories for further analysis, similar to the approach in the *CNVRanger* package, using the following formula:

$$\text{Integer} = \text{round}(2^{\log2(CN/ploidy)} * 2)$$

with the resulting categories defined as:

- **0**: Homozygous deletion (Deletion)
- **1**: Heterozygous deletion (Loss)
- **2**: Normal diploid state (Neutral)
- **3**: One-copy gain (Soft gain)
- **4**: Amplification (Amplification)

For further analysis using SCNA, only amplifications in oncogenes and ambiguous genes, and deletions in TSGs and ambiguous genes, were considered as cancer driver events. For SVs, only homozygous disruptions were detected in this dataset.

Longitudinal and freeze/thaw-cycles samples

Sequencing

For the longitudinal samples, organoids were maintained in culture following the banking of 25 cryovials. Cell pellets were collected for sequencing, with the specific timing determined by the sample characteristics and the designated time-point:

- HCM-SANG-0226-C20: banking date was 2017-03-09 (Time 1); subsequent collections were 24, 95, 154, and 204 days post-banking (Times 2–5)
- HCM-SANG-0278-C20: banking date was 2017-11-13 (Time 1); subsequent collections were 24, 80, 136, and 192 days post-banking (Times 2–5)
- HCM-SANG-0295-C15: banking date was 2016-10-19 (Time 1); subsequent collections were 33 and 86 days post-banking (Times 2–3)
- HCM-SANG-0300-C15: banking date was 2017-06-28 (Time 1); subsequent collections were 33, 86, and 141 days post-banking (Times 2–4)

For the freeze/thaw cycles (time points 1–4), organoids were thawed, maintained in culture for 3–4 passages, and cell pellets were collected for sequencing before the organoids were refrozen. Passaging was performed every 5–7 days.

Timing of WGD in a longitudinal set of organoids

To time the WGD observed in HCM-SANG-0278-C20, we classified highly confident post-WGD mutations as those occurring at timepoints 3, 4, and 5, located in single copies within amplified regions of the genome that display LOH (i.e., minor allele CN = 0, multiplicity < major allele CN, and multiplicity < 2). A total of 1,419 mutations had a CCF close to 1, with the majority also appearing at timepoints 1 (942 mutations, 66.38%) and 2 (1,025 mutations, 72.23%) at a lower CCF (~0.5).

Targeted gene sequencing

Targeted gene sequencing (TGS) was performed on select tumour tissues and organoids using the same sequencing platform, with an average coverage of 800x. This was used to benchmark the sensitivity of WGS. A subset of 225 cancer driver genes was analysed on this data.

A comparison of SNVs and Indels identified using WGS or TGS demonstrated a strong concordance between the two methodologies, even without applying the VAF > 0.05 filter. VAFs exhibited a PCC greater than 0.9 for both organoids and tumours. Notably, of the 2003 SNVs/Indels detected across the subset of 225 cancer driver genes using TGS, 1979 (99%) were also identified by WGS. This supports the sensitivity of our WGS approach to robustly call SNVs.

Comparison between HCMI and Sanger organoids genomic data

Variant call format (VCF) files processed through the Sanger and HCMI consensus pipelines were annotated using a curated set of known cancer driver genes. The intersection of variants

in organoid sample pairs was identified using the intersectBedcommand from BEDTools (v2.18)[106]. We also processed samples sequenced at HCMI using the Sanger pipeline and generated consensus sets of variant calls using high depth (>30x) matched normal samples sequenced at Sanger. The intersection of only *PASSed* variants in organoid sample pairs was performed using bcftools[107] isec command with defaults parameters, resulting variant counts were used for jaccard index calculation.

Comparison between organoid and TCGA genomic alterations

For TCGA samples, binary mutation matrices and discretized copy number data (-1, 0, 1), curated in Pacini et al. (2024)[9], were used for this analysis. The analysis included only COAD/READ samples with MSI status available and samples with COAD and READ codes were merged.

CRISPR-screen data processing

Selection of sgRNAs

Organoids were screened with either Yusa v1.1[3] or the smaller derivative library MinLibCas9[41]. Sixteen models were screened with both libraries. To enable data integration, we exclusively included sgRNAs shared between the two libraries in the data processing, QC assessment and analysis. Applying this filter resulted in a final dataset for each organoid which included 16,562 genes targeted by at least 2 sgRNAs (Extended Data Fig.8a).

QC assessment and filtering

To assess screening quality, we adapted pipelines[3] developed for CRISPR-Cas9 screens performed in cell lines. For this analysis, we selected 800 sgRNAs targeting 400 genes with the highest average pairwise Pearson correlation (>0.508), using fold-change counts normalised by the plasmid across all screened organoids. Normalisation using an early time-point control (day 5) closely mirrored the distribution observed in the plasmid, confirming consistency in sgRNA representation (Extended Data Fig.8d-e). Using gene fold-change counts for the selected genes, we calculated all possible pairwise Euclidean distances among technical replicates, both within each sample and across all samples. This enabled us to estimate a null distribution of replicate distances (Extended Data Fig.8c). We then established a QC threshold (48.75), for which the probability mass function of distances computed between replicates of the same organoid was at least twice that of the null probability mass function. At this stage, 48 screening replicates were discarded. Overall, 21 samples with fewer than two replicates remaining after this QC filtering were removed and 190 non-unique organoids were included in the next processing steps.

sgRNA counts processing and calling gene knockout fitness effects

The analysis set of 190 organoids was further processed using CRISPRcleanR[96] (https://github.com/francescojm/CRISPRcleanR). This adjusts for the effect of library size and read-count distributions[108] and corrects for gene copy number bias[109]. In addition, two commonly used metrics for evaluating binary classifier performance—area under the receiver operating characteristic (ROC) curve and area under the precision-recall (PR) curve—were computed. These metrics assess the classifier's ability to distinguish between broadly considered essential and non-essential genes[110] based on depletion log fold changes, measuring the trade-off between sensitivity and specificity for ROC, and precision and recall for PR. Twelve organoids were discarded due to AUROC and/or AUPR values that were 3 SD lower than the average, leaving 178 non-unique organoids for downstream analysis. The curves for the remaining organoids are shown in **Fig.4c-d**. Finally, the Cohen's D coefficient, which measures the distance between the distributions of positive and negative controls, in this case, essential and non-essential genes was calculated. All replicates had a value greater than 0.8.

ComBat (from sva R package - v3.52.0)[97] was used to correct for batch effects in CRISPRcleanR normalised and copy-number-corrected gene log fold changes for data generated with the two sgRNA libraries. The resulting values served as input for the BAGEL2 method[45] (https://github.com/hart-lab/bagel) to identify significantly depleted genes. Curated reference gene sets previously published[3] (with cancer driver genes removed) were used to define essential and non-essential genes.

Scaling of gene log fold changes (LFCs)

LFCs underwent scaling to improve comparability across organoids by using essential and non-essential genes as positive and negative controls, respectively. For each gene in an organoid, this scaling process involved subtracting the median LFC of non-essential genes from the gene's LFC. The result was then normalised by dividing it by the difference between the median LFC of essential genes and the median LFC of non-essential genes. This approach effectively adjusts gene LFCs based on a standard range defined by control gene sets.

Selection of organoids with data from both CRISPR libraries

To avoid including any duplicate samples in subsequent analyses, we applied three QC metrics to select the organoids with the highest-quality data from each pair screened with both libraries: (1) the number of gene depletions per model, (2) Cohen’s D coefficient, and (3) the true positive rate (TPR). For each model, we assigned a rank for each metric (with a rank of 1

indicating the poorest performance). These ranks were summed, and models with duplicate data that had the lowest total scores were excluded from downstream analysis (Supplementary Table 4).

Biomarker analysis

Selection of gene dependencies

From the genes identified in the differential dependency analysis, we included in the biomarker analysis those that were depleted in at least three organoids and not depleted in at least three organoids. The analysis was performed by cancer type only using COAD/READ and ESCA organoids, and in all gastrointestinal organoids together (COAD/READ, ESCA, PAAD, STAD).

Selection of biomarkers

The biomarkers were represented as either dummy binary variables or continuous values.

- **Clinical features:** binary variables for the cancer stage (I, IV, early [I+II], or advanced [III+IV]), age (younger or older than 50 years old), and sex. For COAD/READ, additional features included whether the organoids originated from metastasis, the localisation of the primary tumour (proximal, distal, or rectum) and the CMS/CRIS clinical subtypes. For ESCA, whether the patient had been diagnosed with Barrett's oesophagus was included.
- **Driver mutations:** SNVs or Indels. In tumour suppressor genes (TSG), only biallelic mutations were considered.
- **Specific variants:** for COAD/READ, variants in *BRAF* and *KRAS* genes were considered at the codon and specific position level. For *KRAS*, all variants in codons other than the G12 were grouped together as “Other KRAS”.
- **Copy number-related features:** we included amplifications (only in oncogenes and ambiguous genes) and large deletions (only in TSG and ambiguous genes) at both gene and chromosome arm levels. For chromosome arm-level analysis, we calculated the weighted average CN for each chromosome arm using segment data and classified them into CN categories. Additionally, we considered SVs, WGD, chromothripsis, focal amplifications and MSI status (only for COAD/READ organoids).
- **Expression:** several genes were excluded based on different criteria. For each cancer type, genes were excluded if: (1) they were not expressed in at least 5% of the organoids, (2) their mean expression level ($\log_2(TPM + 1)$) was < 0.1, (3) they did not have at least three organoids in two of the following three categories: low expressed ($\log_2(TPM + 1) < 1$), expressed ($1 \leq \log_2(TPM + 1) \leq 5$), or highly expressed ($\log_2(TPM + 1) > 5$), or (4) they had a z-score ≥ 2 after a z-test.

- **Mutational signatures:** represented as binary features, indicating whether organoids harboured mutations associated with a specific signature, but only if those mutations accounted for at least 5% of the total mutations.
- **LoF events:** the goal was to combine genomic and transcriptomic features that could have similar biological implications. We included TSG or ambiguous genes with expression levels of log2(TPM + 1) < 0.1, TSG with biallelic alterations, and ambiguous genes with biallelic alterations, but only if these alterations were large deletions or LoF mutations.
- **GoF events:** the goal was to combine genomic and transcriptomic features that could have similar biological implications. We included oncogenes or ambiguous genes with expression levels within the highest 10% of genes per organoid, oncogenes with amplifications or any type of mutation, and ambiguous genes with amplifications or GoF mutations.

For binary markers, we required the marker to be present in at least three organoids and absent in at least three organoids for each cancer type. Any binary feature that was identical to a covariate was excluded. Binary features with identical values were annotated and only one version was included in the analysis. Finally, we performed a chi-square test to identify and annotate other binary features that might be similar based on the results of the test (FDR < 5%).

Linear regression model

Associations between gene dependencies and genomic, transcriptomic, or clinical features were assessed using linear regression models. We tested 24502918, 10,435,425 and 6,920,933 feature-gene associations in gastrointestinal, COAD/READ and ESCA organoids, respectively. To control for potentially spurious associations, several technical and biological covariates were included in the model: ploidy, the CRISPR library used for screening, and MSI status (for COAD/READ organoids only). For each gene dependency-feature pair, we fit the following linear regression model:

$H_0 : y = \beta_0 * C + \varepsilon$

$H_1 : y = \beta_0 * C + \beta_1 * F + \varepsilon$

The hypotheses included y as the gene dependency, F as the genomic, transcriptomic, or clinical feature, C as the matrix of covariates and ε as the general noise term. For each association, we constructed both a null model (H0 – regression with only covariates) and an

alternative model (H0 – regression with both covariates and the feature of interest) to account for the impact of covariates. We used a likelihood-ratio test (LRT, lrtest function in R) to evaluate the significance of the difference between the two models, and p-values were adjusted per gene dependency and feature class (clinical, mutation/variant, CN, expression, signatures, GoF and LoF) using the Benjamini-Hochberg method.

In addition to the regression coefficient, R^2 and p value, and the adjusted p value of the LRT, we also calculated the effect size using Cohen's f^2 coefficient:

$$f^2 = R^2 / (1 - R^2)$$

The results table also included summary statistics for gene LFCs for each group (with and without the biomarker): the mean, median, minimum, maximum, and standard deviation, as well as the differences in mean and median between groups. For continuous features, such as expression levels, the groups were defined by the upper and lower 25% quantiles.

Significant associations

Only associations with an adjusted p-value < 0.05 with an effect size within the top absolute 10% were considered significant. Significant associations were further classified based on effect size and the LFC difference between the two groups in the linear regression model:

- **Class A**: Mean LFC difference > 0.75 and effect size within the top 5% of associations
- **Class B**: Mean LFC difference > 0.5
- **Class C**: All other significant associations

A table was generated listing all genes from the differential dependency analysis, indicating whether each gene had an associated significant biomarker (Supplementary Table 8). If multiple biomarker significance classes were associated with a gene, only the highest significance class was retained. To determine if biomarkers were specifically associated with the MSI status, a Fisher's exact test was performed to assess exclusivity within that population (FDR < 25%). For cases where more than one category shared the same biomarker significance class for a particular feature (e.g., *KRAS* mutation and *KRAS* GoF), the simplest category was retained (i.e., *KRAS* mutation).

Supplementary References

104. Harris, P. A. *et al.* The REDCap consortium: Building an international community of software platform partners. *J Biomed Inform* **95**, 103208 (2019).

105. Raine, K. M. *et al.* ascatNgs: Identifying Somatically Acquired CopyNumber Alterations from Whole Genome Sequencing Data. *Curr Protoc Bioinformatics* **56**, 15.9.1-15.9.17 (2016).

106. Quinlan, A. R. & Hall, I. M. BEDTools: a flexible suite of utilities for comparing genomic features. *Bioinformatics* **26**, 841–842 (2010).

107. Danecek, P. *et al.* Twelve years of SAMtools and BCFtools. *Gigascience* **10**, (2021).

108. Anders, S. & Huber, W. Differential expression analysis for sequence count data. *Genome Biol* **11**, R106 (2010).

109. Aguirre, A. J. *et al.* Genomic Copy Number Dictates a Gene-Independent Cell Response to CRISPR/Cas9 Targeting. *Cancer Discov* **6**, 914–929 (2016).

110. Hart, T. & Moffat, J. BAGEL: a computational framework for identifying essential genes from pooled library screens. *BMC Bioinformatics* **17**, 164 (2016).

Supplementary Table Captions:

Supplementary Table 1: Summary of derivation data and QC status for all organoid derivations attempted.

Supplementary Table 2: Overview of organoid meta-data, including their unique identifiers, genomic and functional datasets available for organoids and their corresponding tumours, along with patient clinical data.

Supplementary Table 3: Comprehensive data from WGS of organoids and tumours, including direct comparisons between organoid and tumour samples. Detailed annotations of genomic driver events, SBS96 mutational signatures, TPM values derived from RNA sequencing data, and CMS and CRIS classification for COAD/READ organoids.

Supplementary Table 4: CRISPR screening QC metrics and identification of organoid core fitness genes for models that passed the CRISPR screening quality criteria.

Supplementary Table 5: Binary matrix classifying significant fitness genes identified in each organoid model using the BAGEL method.

Supplementary Table 6: Normalised, copy-number-corrected, and batch effect-corrected gene level LFCs from CRISPR screens, following an adjustment based on essential and non-essential gene classifications.

Supplementary Table 7: Results of differential dependency analysis based on CRISPR LFCs for COAD/READ and ESCA organoids, highlighting genes with differential dependency patterns across models.

Supplementary Table 8: Statistical associations between genomic, transcriptomic, or clinical biomarkers and CRISPR LFCs in all gastrointestinal, COAD/READ and ESCA organoids, including adjusted p-values, effect sizes, and classification into biomarker significance classes (A, B, or C). In addition, genes from differential dependency analysis (Supplementary Table 7) with the most significant biomarker annotated, if any.

Supplementary Table 9: IC_{50} values and Bliss synergy scores for EGFR/ERBB2 and KRAS inhibitors, tested as monotherapies and in combination.

Supplementary Table 10: Summary of all tables and datasets used in this study. For each file, the table indicates its location (Supplementary Information or Figshare), a description of the data it contains, the data source, and the analyses, code, or figures in which it was used.
