# Supplementary material for: A tumour-derived organoid biobank maps cancer gene dependencies
Source: Nature. 2026 Aug 5;657(8132):765–74. doi: 10.1038/s41586-026-10830-y (PMC13581617; doi:10.1038/s41586-026-10830-y)
Supplement: Supplementary file 2 [file 41586_2026_10830_MOESM2_ESM.pdf]

nature portfolio

Corresponding author(s): Mathew Garnett

Last updated by author(s): 03/03/2026

Reporting Summary

Nature Portfolio wishes to improve the reproducibility of the work that we publish. This form provides structure for consistency and transparency in reporting. For further information on Nature Portfolio policies, see our Editorial Policies and the Editorial Policy Checklist.

Statistics

For all statistical analyses, confirm that the following items are present in the figure legend, table legend, main text, or Methods section.

n/a	Confirmed	
☐	☒	The exact sample size (*n*) for each experimental group/condition, given as a discrete number and unit of measurement
☒	☐	A statement on whether measurements were taken from distinct samples or whether the same sample was measured repeatedly
☐	☒	The statistical test(s) used AND whether they are one- or two-sided<br>*Only common tests should be described solely by name; describe more complex techniques in the Methods section.*
☐	☒	A description of all covariates tested
☐	☒	A description of any assumptions or corrections, such as tests of normality and adjustment for multiple comparisons
☐	☒	A full description of the statistical parameters including central tendency (e.g. means) or other basic estimates (e.g. regression coefficient) AND variation (e.g. standard deviation) or associated estimates of uncertainty (e.g. confidence intervals)
☐	☒	For null hypothesis testing, the test statistic (e.g. *F*, *t*, *r*) with confidence intervals, effect sizes, degrees of freedom and *P* value noted<br>*Give P values as exact values whenever suitable.*
☒	☐	For Bayesian analysis, information on the choice of priors and Markov chain Monte Carlo settings
☒	☐	For hierarchical and complex designs, identification of the appropriate level for tests and full reporting of outcomes
☐	☒	Estimates of effect sizes (e.g. Cohen's *d*, Pearson's *r*), indicating how they were calculated

Our web collection on statistics for biologists contains articles on many of the points above.

Software and code

Policy information about availability of computer code

Data collection

The RedCap application was used to securely capture pseudo-anonymised clinical data for each sample, including patient information, tumour and molecular pathology and treatment (https://projectredcap.org/)

Data analysis

All scripts used for the main analyses are deposited in the Garnett Lab GitHub repository (https://github.com/Garnett-Lab/SangerOrganoidBiobank).

List of programs and softwares:
- BWA-MEM: version 0.7.17 (https://sourceforge.net/projects/bio-bwa/)
- cgpCaVEMan: version 1.13.14/1.14.1/1.15.0/1.15.1 (https://github.com/cancerit/CaVEMan)
- ASCAT NGS: version 4.2.1/4.3.2/4.3.3/4.5.0 (https://github.com/cancerit/ascatNgs)
- cgpPindel: version 3.3.0/3.5.0 (https://github.com/cancerit/cgpPindel)
- vafCorrect: version 2.4.0 (https://github.com/cancerit/vafCorrect)
- AMBER: version 3.5 (https://github.com/hartwigmedical/hmftools)
- COBALT: version 1.11 (https://github.com/hartwigmedical/hmftools)
- SAGE: version 2.8 (https://github.com/hartwigmedical/hmftools)
- SnpEff: version 5.0 (https://github.com/pcingola/SnpEff)
- GRIDSS: version 2.12.0 (https://github.com/PapenfussLab/gridss)
- RepeatMasker: version 4.1.2 (https://www.repeatmasker.org/)
- kraken2: version 2.1.2 (https://genomebiology.biomedcentral.com/articles/10.1186/s13059-019-1891-0)
- GRIPSS: version 1.9 (https://github.com/hartwigmedical/hmftools)
- PURPLE: version 2.54 (https://github.com/hartwigmedical/hmftools)

• SigProfilerExtractor: version 1.2.0 (https://github.com/AlexandrovLab/SigProfilerExtractor)
• SigprofilerAssignment: version 0.2.0 (https://github.com/AlexandrovLab/SigProfilerAssignment)
• CHORD: version 2.0.3 (https://github.com/UMCUGenetics/CHORD)
• ShatterSeek: version 1.1 (https://github.com/parklab/ShatterSeek)
• STAR: version 2.5.0c (https://github.com/cancerit/cgpRna)
• RSEM: version 1.3.3 (https://bmcbioinformatics.biomedcentral.com/articles/10.1186/1471-2105-12-323)
• CMSCaller: version 0.99.2 (https://github.com/peterawe/CMScaller)
• BEDTools: version 2.18 (https://github.com/arq5x/bedtools2)
• BAGEL: version 2 (https://github.com/hart-lab/bagel)
• R: version 4.4.2
• GenomicRanges (v1.56.1), data.table (v1.15.4), tidyverse (v2.0.0), CRISPRcleanR (v3.0.1), sva (v3.52.0), digest (v0.6.36), scales (v1.3.0), lmtest (v0.9-40), dplyr (v1.1.4), CoRe (v1.0.0), msigdbr (v7.5.1), clusterProfiler (v4.12.2), gdscIC50 (v1.7.3)

For manuscripts utilizing custom algorithms or software that are central to the research but not yet described in published literature, software must be made available to editors and reviewers. We strongly encourage code deposition in a community repository (e.g. GitHub). See the Nature Portfolio guidelines for submitting code & software for further information.

Data

Policy information about availability of data

All manuscripts must include a data availability statement. This statement should provide the following information, where applicable:

- Accession codes, unique identifiers, or web links for publicly available datasets
- A description of any restrictions on data availability
- For clinical datasets or third party data, please ensure that the statement adheres to our policy

The raw WGS, TGS, RNA-seq and CRISPR data from organoids used in this study, along with matched tumour tissues and normal samples, have been deposited in the European genome-phenome archive (EGA, accession number: EGAD00001015469 - WGS, EGAD00001015468 - TGS, EGAD00001015470 - RNA). Processed genomic, transcriptomic and CRISPR data is available in Cell Model Passports (https://cellmodelpassports.sanger.ac.uk/). Additional datasets required to perform analyses presented in this manuscript are available on Figshare: https://doi.org/10.6084/m9.figshare.28339340.

Other data accessed:
• Intogen Gene annotation: https://www.intogen.org/download
• COSMIC Cancer Gene Census: https://cancer.sanger.ac.uk/cosmic/census?tier=1
• Cancer Genome Interpreter (CGI) catalog of validated oncogenic mutations: https://www.cancergenomeinterpreter.org/mutations
• Memorial Sloan Kettering Cancer Center (MSKCC) Hotspots version 2: https://www.cancerhotspots.org/swagger-ui.html
• BoostDM dataset: https://www.intogen.org/boostdm/downloads
• Cancer Predisposition Variant (CPV) dataset: S2A in https://www.sciencedirect.com/science/article/pii/S0092867418303635?via%3Dihub
• ENCODE blacklist: https://github.com/Boyle-Lab/Blacklist
• COSMIC Reference Mutational Signatures 3.4: https://cancer.sanger.ac.uk/signatures/

Data used from previous larger-scale cancer cell lines CRIPSR screenings or TCGA data manually curated:
Pacini, C. et al. A comprehensive clinically informed map of dependencies in cancer cells and framework for target prioritization. Cancer Cell 42, 301–316.e9 (2024).

Research involving human participants, their data, or biological material

Policy information about studies with human participants or human data. See also policy information about sex, gender (identity/presentation), and sexual orientation and race, ethnicity and racism.

Reporting on sex and gender	This study used the sex variable to describe the patients cohort where we got the samples from. The sex variable was collected from the RedCap electronic data capture tool based on patients medical records. Patient consent and ethical permits were obtained for the use of this data. The samples collected in this study were from both female (85/256 - 33.2%) and male (171/256 - 66.8%) sex individuals.
Reporting on race, ethnicity, or other socially relevant groupings	The ethnicity variable was collected from the RedCap electronic data capture tool based on patient records but we haven't performed any ancestry-related analysis.
Population characteristics	Detailed clinical data is provided in Supplementary Table 2.1. The cohort includes patients diagnosed with colorectal (132), oesophageal (76), ovarian (20), pancreatic (22), or stomach (6) cancer. Ethnicity data includes White British (222), White and Black Caribbean (2), White Irish (1), White Asian (1), Pakistani (1), Indian (1), and not stated or unknown (28). Patient ages range from 34 to 90 years. Samples were collected from both female (85) and male (171) individuals. Tumour samples were derived from either primary tumours (212) or metastatic lesions (43). Additional clinical data, including treatment history, comorbidities, surgical procedures, tumour localization, and patient lifestyle factors, were also collected.
Recruitment	Patients undergoing biopsy or surgical resection at multiple NHS clinical sites were consecutively recruited and invited to donate resected tumour tissue and matched blood samples, with a subset of samples collected from additional research biopsies with fully informed consent. The cohort includes samples from patients both with and without prior therapy, as well as samples from metastatic lesions. The clinical sites are listed in the section below and their expertise in these specific cancer types ensured the access to high quality samples and leveraged existing clinical knowledge.<br><br>Several potential considerations inherent to this study design should be taken into account. First, as recruitment was largely restricted to patients undergoing surgical resection or biopsy, the cohort may over-represent resectable tumours, although the inclusion of metastatic lesions and samples from patients with prior therapy partially mitigates this limitation. Second,

inclusion depended on the availability of spare tissue after clinical use, which may favour larger tumours or cancer types where resection yields more material. Third, recruitment was limited to NHS centres in the United Kingdom, which may not fully reflect the genomic and clinical diversity of cancer patients globally. Finally, not all samples successfully established as organoid lines, and certain tumour characteristics may be associated with derivation success. However, we have tested this hypothesis and did not find a relationship between any derivation metric and genomic/transcriptomic characteristics of the tumour.

Ethics oversight

Patient tissue samples used in this study were received from multiple NHS clinical sites:
● University Hospital Birmingham via the Human Biomaterial Resource Centre (HBRC). The participant recruitment process, information sheets and consent forms have previously been given a favourable opinion by North West – Haydock Research Ethics Committee (REC), REC reference number 15/NW/0079, sub-approval project code 17-287.
● University of Cambridge and Cancer Research UK (Cambridge Institute). Clinical data and tissue samples for the patients were collected on the prospective cohort study Cambridge Translational Cancer Research Ovarian Study 04 (CTCR-OV04), with IRAS project ID 4853, and which was approved by the Institutional Ethics Committee (REC reference number 08 / H0306/61).
● Addenbrookes Hospital, Cambridge, via the OCCAMS project. The OCCAMS participant recruitment process, information sheets and consent forms have previously been given a favourable opinion by Cambridge South REC, REC reference number 10/H0305/1.1.
● Application to access patient tissue and organoids was authorised by the NHS Greater Glasgow and Clyde Biorepository under their NHS REC approval with ethical approval granted in biorepository application 22/WS/0020.
● Samples at Imperial College London were collected under the authority of Imperial College Healthcare Tissue and Biobank (HTA licence 12275, REC reference number 22/WA/0214, IRAS reference 312147, project number N19001-5A). All patients gave written informed consent.
● University of Southampton Biobank (HTA licence no. 12009) through the Pancreatic Cancer: Molecular and Genetic Mechanisms project. The participant recruitment process, information sheets and consent forms for this project have previously been given a favourable opinion, REC reference number: 10/H0502/72. The Molecular Pathology of Colorectal Cancer project, REC reference number: 07/H0504/125, the Evolution of abdomino-pelvic cancers under immune selection study, REC reference number: 21/EE/0110 and the Upper Gastro-Intestinal Tumour Ecology study IRAS 233065 Southampton General Hospital provided samples through the OCCAMS project using the same REC approved documentation used by Addenbrooke's Hospital. In addition, where patients undergoing surgery have consented for use of their surplus samples in research, these may also be received from Southampton Biobank.

To conduct this work and to ensure the derived organoids could be used for academic and commercial purposes, all necessary ethical approvals were obtained from each participating clinical sites for the collection of patient samples as well as for the Wellcome Sanger Institute for organoid derivation (IRAS ID:203519; REC 16/LO/1110).

Note that full information on the approval of the study protocol must also be provided in the manuscript.

Field-specific reporting

Please select the one below that is the best fit for your research. If you are not sure, read the appropriate sections before making your selection.

☒ Life sciences ☐ Behavioural & social sciences ☐ Ecological, evolutionary & environmental sciences

For a reference copy of the document with all sections, see nature.com/documents/nr-reporting-summary-flat.pdf

Life sciences study design

All studies must disclose on these points even when the disclosure is negative.

Sample size

Organoid derivation was attempted for all available patient tumour samples collected during the study period (907 samples from 867 unique donors couriered to the Sanger Institute), and no statistical methods were used to predetermine sample size. The final sample size was therefore determined by the number of patient samples successfully established as organoid models following predefined quality control criteria, rather than by a priori power calculations.

Of the 907 samples processed, 256 independent patient-derived organoid lines passed QC and were included in the biobank. Whole-genome sequencing was performed for all 256 organoid models, as well as for 171 matched original tumour samples for which sufficient material remained after organoid derivation. RNA sequencing was performed for 255 models, and CRISPR screening for 164. Drug sensitivity assays were conducted in subsets of models selected based on specific experimental objectives, including genotype-defined cohorts and models with matched CRISPR screening data to enable integrative functional analyses.

Data exclusions

Of the 907 patient tumour samples processed, 651 failed to produce a viable organoid line and were therefore excluded from all analyses. Reasons for derivation failure included short-term cultures (the most frequent cause), lack of input cells, failure to form organoids, bacterial/fungal contamination, fibroblast contamination, absence of cancer driver mutations, slow culture growth, cross-contamination, user error, and unmatched normal samples, as detailed in Extended Data Figure 1 and Supplementary Table 1.

For CRISPR screens, a total of 203 screens were conducted across the organoid collection; of these, 180 screens in 164 unique organoid lines passed all QC criteria, including replicate correlation (Euclidean distance), and classification performance of essential and non-essential genes assessed by ROC and PR curves. The 23 screens that did not meet QC thresholds were excluded from the analysis, as detailed in the Methods section.

Stomach organoids were not described in detail, specially in the genomic characterization and CRIPSR screens, due to the small sample size.

Replication

By the nature of this biobank study, organoid derivation and genomic sequencing (whole-genome and transcriptomic) were performed once

Replication	per sample, as each model represents a unique patient-derived biological specimen. However, to confirm the genomic integrity and reproducibility of the biobanked models, all organoid lines underwent a freeze/thaw quality control process followed by targeted genome sequencing, confirming that models retained their genomic characteristics after cryopreservation.<br><br>For CRISPR screens, a minimum of three technical replicates were performed per screen. To ensure robustness of the results, three organoid lines were independently re-screened from the Cas9 transduction stage, and results were reproducible.<br><br>For high-throughput drug sensitivity screens, two independent projects were performed with partial overlap in compounds tested. Compounds were tested in biological duplicates in the first project and in single biological replicates in the second project. Agents included in multiple drug combinations, such as afatinib, had higher technical replication as they were also tested as monotherapies. For validation drug sensitivity assays, compounds were tested in biological duplicates with a minimum of three technical replicates per condition.
Randomization	Randomisation was not applicable to this study, as this is a biobank resource study rather than a controlled interventional experiment. Patient samples were collected prospectively from consenting donors across multiple clinical centres; however, all samples were processed, derived, sequenced, and analysed at a single centre (the Sanger Institute), eliminating inter-centre processing variability as a potential covariate.<br><br>The only potential covariate considered was sample preservation method (fresh vs. frozen). To assess whether this introduced any systematic bias, organoids were derived from paired fresh and frozen samples from two donors and the resulting genomic profiles were compared. No meaningful differences were observed, supporting the comparability of models derived from both sample types. Beyond this, no additional covariates were identified that required controlling for in the analyses
Blinding	Blinding was not applicable to this study. The primary outputs of this study are genomic characterisation and functional profiling of patient-derived organoid models, which are objective measurements (e.g., mutational profiles, gene expression, drug sensitivity values, CRISPR screen scores) generated through automated pipelines and computational analyses. These analyses are not subject to observer bias that blinding would mitigate. Sample identity was necessarily known during organoid derivation and processing to enable correct clinical annotation and quality control tracking. For functional assays such as drug sensitivity and CRISPR screens, results were processed using standardised, pre-established computational pipelines, further minimising the risk of bias.

Reporting for specific materials, systems and methods

We require information from authors about some types of materials, experimental systems and methods used in many studies. Here, indicate whether each material, system or method listed is relevant to your study. If you are not sure if a list item applies to your research, read the appropriate section before selecting a response.

Materials & experimental systems

n/a	Involved in the study
☒	☐ Antibodies
☒	☐ Eukaryotic cell lines
☒	☐ Palaeontology and archaeology
☒	☐ Animals and other organisms
☒	☐ Clinical data
☒	☐ Dual use research of concern
☒	☐ Plants

Methods

n/a	Involved in the study
☒	☐ ChIP-seq
☒	☐ Flow cytometry
☒	☐ MRI-based neuroimaging

Plants

Seed stocks	*Report on the source of all seed stocks or other plant material used. If applicable, state the seed stock centre and catalogue number. If plant specimens were collected from the field, describe the collection location, date and sampling procedures.*
Novel plant genotypes	*Describe the methods by which all novel plant genotypes were produced. This includes those generated by transgenic approaches, gene editing, chemical/radiation-based mutagenesis and hybridization. For transgenic lines, describe the transformation method, the number of independent lines analyzed and the generation upon which experiments were performed. For gene-edited lines, describe the editor used, the endogenous sequence targeted for editing, the targeting guide RNA sequence (if applicable) and how the editor was applied.*
Authentication	*Describe any authentication procedures for each seed stock used or novel genotype generated. Describe any experiments used to assess the effect of a mutation and, where applicable, how potential secondary effects (e.g. second site T-DNA insertions, mosiacism, off-target gene editing) were examined.*
