# Supplementary material for: A tumour-derived organoid biobank maps cancer gene dependencies
Source: Nature. 2026 Aug 5;657(8132):765–74. doi: 10.1038/s41586-026-10830-y (PMC13581617; doi:10.1038/s41586-026-10830-y)
Supplement: Supplementary file 3 [file 41586_2026_10830_MOESM4_ESM.pdf]

nature portfolio

Peer Review File

A tumour-derived organoid biobank maps cancer gene dependencies

Corresponding Author: Dr Mathew Garnett

Any redactions in this file are there to maintain patient confidentiality, the confidentiality of unpublished data, or to remove third-party material.

This file contains all reviewer reports in order by version, followed by all author rebuttals in order by version.

Version 0:

Reviewer comments:

Referee #1

(Remarks to the Author)
The authors Herranz-Ors et al. report the derivation and genomic and transcriptomic characterization of 256 tumor organoids from 5 different cancer types. Out of the established lines, the authors carried out genome-wide CRISPR screens in 98 lines with 2 sgRNA libraries. From the genetic screens, the authors found many previously known genetic decencies, as well as some new ones. Although this work provides limited new scientific findings, the resources will be very useful to the cancer research community (provided that it is deposited accessibly and that the annotation is improved). While the sequencing analyses were done thoroughly, there remains ambiguity in the results and intepretation.

Major
To enhance the user experience and the impact of this resource, the authors should provide a detailed instruction manual that references for each tumor type / each established model all the available datasets, the used analysis code, corresponding SOPs, regulatory information & restrictions (MTA, controlled access data procedures).
The deposited code lacks a readme file, please annotate how the manuscript figures can be produced from the deposited raw data. The figshare deposit needs a citable and permanent DOI.
Authors write in line 166 that they observed a “ high concordance of somatic variants”, which corresponds to lower than 70% shared SNVs for MSS samples. How much concordance would be observed across tumors within the same tumor type? Could the authors discuss the reasons for the observed low fractions of shared SNVs and Indels? (ext data fig 2d)

Line 170: the authors mention an undisclosed commercial entity that will sell the established lines from Q4 2025. Authors have to ensure that distribution availability is in place by the publication of the manuscript. I think that a non-profit repository that ensures open, equitable access to patient-donated, taxpayer-funded models, would maximize public benefit and transparency. This approach would align with academia's mission to promote unrestricted sharing of research resources for societal advancement, rather than commercial profit.

Metadata: line 178: at least 25 annotations: It’s not about quantity but quality: Based on Supp table 2, survival and outcome data are not available for this cohort. This limits the usability; the authors should assemble these data types and add them to the metadata section.

Do organoids that passed quality control (QC) differ in mutational profiles or driver mutations from those that failed QC? The number of days until the first passage varied substantially; could genetic differences underlie this variability?

The authors conducted CRISPR screens on 98 organoid lines, each subjected to three rounds of lentiviral transduction (Cas9 transduction, sgRNA testing, genome-wide library). It is unclear whether validations were performed using freshly prepared batches of organoids. Additionally, the manuscript lacks validation of hits using individual sgRNAs. The authors should clarify these points and ideally perform validations with distinct batches of organoids and specific sgRNAs.

The organoid pair HCM-SANG-0299-C15-A and -B, included in the screen, exhibited differences in the number of depleted genes. Could the authors provide insights into how treatment history or disease progression might influence genetic

dependencies?

The authors report increased genetic dependency on MDM2 in wild-type p53 and KrasG12D/Kras mutant COAD/READ organoids. However, the subsequent validation with Nutlin-3a treatment is incomplete and insufficiently convincing, given that many data points are missing. Only approximately half of the organoids identified in the screen as depleted for MDM2 dependency responded to Nutlin-3a. The authors should carry out Nutlin-3a treatment or MDM2 knockout experiments in the missing data points to strengthen the validation.

The statement that "KRAS wild-type organoids have increased dependency on EGFR, ERBB2, and PTPN11" currently lacks statistical analysis or experimental validation. The authors should provide appropriate statistical tests or additional experimental evidence supporting this claim.

Could the authors specify clearly the genetic background of the organoids labeled as "WT"? Are these organoids wild-type for KRAS but mutated in other components of the EGFR-MAPK pathway? Additionally, could the authors comment on why Gefitinib appears ineffective in WT cells, whereas Afatinib alone is effective in KrasG12D-mutant organoids, contrasting previous findings (e.g., PMID: 27845624)?

Minor:
Referring to “methods”, specify exact location

Numbers of successfully established STAD models seem to be discrepant: Figure 1b reports 6 models passing QC, Ext Data Fig 1b reports 3. Which one is correct?

Please improve the resolution of Fig 1a.

Based on the data in Supp Figure 2 it remains unclear to me, which models were derived from metastases and which were derived from primary tumors. This should be better annotated.

Why was WGS only conducted on 249 of the 256 organoid lines? Will the author add the remaining WGS data?

Drug sensitivity and cell viability assays utilized fluorescent readouts. Could the authors clarify how this method was adapted for organoid cultures? Specifically, were organoids dissociated before fluorescence measurement, or was another approach employed?

Referee #2

(Remarks to the Author)
I co-reviewed this manuscript with one of the reviewers who provided the listed reports.

Referee #3

(Remarks to the Author)
Garnett’s team describes an establishment of a patient-derived organoid biobank that encompasses colorectal, esophageal, ovarian, pancreatic and gastric cancer. The biobank is equipped with rich clinical, genomic and transcriptomic information, and genome-wide dropout CRISPR screening was conducted in a subset of organoid lines, leading to the discovery of some new fitness genes and drug targets. The authors state that all organoid lines will be available through a commercial vendor, and along with the accompanying papers, this work will provide a great resource for basic and preclinical research communities. While the effort spent to derive and analyze the organoids is very impressive, establishment and collection of organoids from major cancer types has been repeatedly reported previously, and insights from this work may not transcend our current knowledge from the compilation of prior studies on patient-derived organoid biobanks in the absence of some strong, new and compelling data that strongly drives forward the cancer research field. Below are my specific comments.

Major
1. The success rate of stable organoid culture appears to be exceeding low especially for colorectal cancer organoids compared to prior studies (van de Wetering et al., Cell 2015; Fujii et al., Cell Stem Cell 2016; Schütte et al., Nature Communications 2017). What do the authors think does this difference and the low take rake arise from? The low establishment rate can result in a potential bias of the biobank toward some “easy-to-establish” cancer types or subtypes. Were there any clinical or perhaps technical variables associated with establishment failure? For instance, with respect to technical experience, does the establishment rate differ between the first and second halves of the study period? Was the same establishment procedure used throughout the study period, or was it modified to improve establishment efficiency?

2. The authors performed CRISPR screening in a large panel of patient-derived cancer organoids, which is impressive. However, dependency genes they found may be lineage-specific, but not cancer-specific, making them not strong enough as drug target candidates. One of the majors strengths of organoid technology is the ability to stably expand normal epithelial tissues. As the goal of CRISPR screening in this study was to identify cancer vulnerabilities, the paper strongly merits from adding and analyzing CRISPR screening of normal organoids and perhaps discovery of cancer-specific genetic

dependency.

3. Also, according to Methods, the authors used 1.25 or 3.3 x 10^7 cells with MOI = 0.3 for genome-wide CRISPR screening. How were these cell numbers determined? I consider this scale is not large enough to achieve sufficient coverage and depth of the guides, and to maintain the library diversity in every model. Not all cells can form organoids or spheroids after single cell dissociation, and the colony-formation efficiency can significantly differ among the models. For instance, if you use 1.25 x 10^7 cells with an MOI of 0.3 and Yusa v.1.1 sgRNA library with an library size of 10^5, assuming a colony formation efficiency of 0.2 (which is a fair value for normal organoids but can be lower in cancer organoids), a rough calculation shows that you only get 7.5 clones per sgRNA. The authors appear to have calculated fold change values based on the read counts from plasmids and post-screen models, but were all the guide RNAs detected at early timepoints after infection?

4. Validation using in vivo experiments is completely lacking. Although the main focus of this article may be to show the fidelity of patient-derived organoids and applicability of CRISPR screening, the reviewer really thinks that some (but not all) sensitivity data should be validated using xenograft models of pertinent organoid lines.

Minor
1. In lines 201–202, the authors mention “Organoid derived from metastatic lesions were almost entirely derived from stage IV patients”, but this is confusing, as patients with distant metastasis automatically have a stage IV disease. What does the derivation of metastatic organoids from stage II and III patients in Extended Data Fig. 3b mean?

2. What are the definitions of the reasons of failure in Extended Data Fig. 1a? These should be stated in the legends or Methods. For example, what is the difference between slow culture growth, culture senescence and short-term culture? How do “normal tissue sample” and “no cancer drivers found” differ?

Referee #4

(Remarks to the Author)
This work reports the generation of 256 cancer organoid models, 123 of which are distinct from the HCMI models described in a companion paper. This paper represents a significant effort to establish a large biobank of organoid models for broad distribution within the scientific community. The study exemplifies the successful collaboration of multiple research teams necessary to develop such a valuable resource.

The techniques and approaches in the resource generation overall are appropriate. The inclusion of matched samples of multiple tumors/timepoints from the same individual will be extremely useful.

Major concerns
• There is an opportunity missed to determine whether the expense and low success rate of organoid modeling is needed, or whether traditional 2D cancer cell line models would show the same 1) success rate and 2) cancer dependencies. This group seems to be one of the only groups that are equipped to address this burning question, and unfortunately it was not addressed or even discussed at all. Based on this group’s experience, would they recommend further investment in organoid generation, or in 2D cell line generation?
• Although the authors demonstrate that these models survive a single freeze-thaw cycle and meet QC metrics, it is crucial to assess whether genomic and phenotypic stability is maintained over multiple freeze-thaw cycles (meaning can users expand and store these models for long-term use or not). Establishing such stability is essential to ensuring the widespread usability, replicability and reliability of these models for researchers.
• The extremely unique data that this resource is providing is the CRISPR dataset, but it is underexplored. It appears raw data is shared, but will the CRISPR datasets be added to DepMap or other CRISPR resources?
• A more systematic analysis of the organoid-specific vulnerabilities should be performed that goes beyond gene-set enrichment analysis. Are there different dependencies identified in this approach compared to cell lines, and are these different due to the new genetic architecture of these new models, or due to culture conditions?
• The same cell line or organoid models should be screened in 2D vs 3D to understand to what effect the culture conditions rather than the model properties influence the cancer dependency landscape.

Minor concerns:

● A reviewer note on p. 5 indicates the models will be distributed commercially – please update vendor/information prior to publication. Overall, this paper is an incredible resource of models. However, the impact of this work will be limited by the commercial distribution of these models which will be cost prohibitive for many investigators.
● It would be valuable to determine whether clinical factors were significantly associated with the inability to establish organoid models, such as patient age, sex, or tumor stage.
● P17 line 556-558 – the increased dependency of KRAS wt organoids on EGFR, PTPN11, and ERBB2 in Fig 5a is not readily apparent in the heatmap visualization and this statement should be strengthened with a statistical approach and an alternative data visualization
● In the methods, please cite specific protocols at protocols.io that were used. Many investigators may want to replicate the approaches used here, but the information seems to be missing from the Methods.
● The lollipop plots in Extended Data Fig 6 are confusing. Please number or color the sample IDs above and put the

numbering on y axis to make meaning more readily apparent.
● Extended Data Fig 8 needs lettering to break up the figure and provide clarity with the link to the figure legend descriptions.

Referee #5

(Remarks to the Author)
I co-reviewed this manuscript with one of the reviewers who provided the listed reports.

Version 2:

Reviewer comments:

Referee #1

(Remarks to the Author)
We appreciate the thorough revisions made to the manuscript and have no further questions.

Referee #2

(Remarks to the Author)
I co-reviewed this manuscript with one of the reviewers who provided the listed reports.

Referee #3

(Remarks to the Author)
The authors have addressed all raised concerns, and I have no further comments.

Referee #4

(Remarks to the Author)
Thank you to the authors for their responses and revisions. They have addressed all of our questions and concerns.

Referee #5

(Remarks to the Author)
I co-reviewed this manuscript with one of the reviewers who provided the listed reports.

Open Access This Peer Review File is licensed under a Creative Commons Attribution 4.0 International License, which permits use, sharing, adaptation, distribution and reproduction in any medium or format, as long as you give appropriate

credit to the original author(s) and the source, provide a link to the Creative Commons license, and indicate if changes were made.
In cases where reviewers are anonymous, credit should be given to 'Anonymous Referee' and the source.
The images or other third party material in this Peer Review File are included in the article’s Creative Commons license, unless indicated otherwise in a credit line to the material. If material is not included in the article’s Creative Commons license and your intended use is not permitted by statutory regulation or exceeds the permitted use, you will need to obtain permission directly from the copyright holder.
To view a copy of this license, visit https://creativecommons.org/licenses/by/4.0/

Mathew Garnett
Senior Group Leader
Wellcome Sanger Institute
T: +44 (0)1223 494 878
E: mg12@sanger.ac.uk

24th November, 2025

Response to Referees

Manuscript Title: A tumour-derived organoid biobank defines a functional map of gene dependencies

Manuscript number: 2025-02-02750B

We thank the referees for their thoughtful and constructive comments. We provide here a full point-by-point response, including appropriate new data, which we believe have addressed the concerns raised and as a consequence substantially improved our manuscript. Our responses are in blue font. Our response should be cross referenced with the accompanying shared statement drafted together with teams from the HCMI network and Broad Institute, which addresses cross-cutting themes relevant to all three manuscripts under review.

In addressing the referees' feedback on our manuscript, we have provided additional datasets that further reinforce the value of the biobank as a resource and deepen the biological insights offered in the manuscript. These include:

- Multiple lines of evidence demonstrating the re-usability and stability of organoid cultures, and the reproducibility of our CRISPR datasets.
- We have added genomic and transcriptomic datasets, making these complete for all models in the biobank.
- We have incorporated whole-genome CRISPR/Cas9 screens for 66 additional organoid models, bringing the total to 164 organoids, with expanded representation of oesophageal cancers and colorectal metastases—two cancer types with a poor prognosis for patients and a pressing need for improved in vitro models.
- We extended analysis of colorectal organoids harbouring distinct KRAS mutant alleles, including a comparative analysis to 6 different KRAS inhibitors, demonstrating remarkable heterogeneity of response, and KRAS-variant allele specific requirements for RAS and EGFR signalling, with direct relevance to the development of KRAS-targeted therapies.
- We have incorporated genetic constraint - the degree to which a gene is intolerant to heterozygous variation as determined from human population studies - to annotate organoid essential genes, providing insights into novel core fitness genes and the potential therapeutic window for dependencies.
- All raw and processed data associated with this study will be made publicly accessible through established repositories: raw sequencing data via EGA, processed sequencing and CRISPR data through Cell Model Passports and DepMap Miner, and all supplementary tables required to reproduce the analyses via Figshare. In addition, the complete analysis code and figure-generation scripts will be made available in the Garnett Lab GitHub repository, accompanied by a comprehensive README file to ensure reproducibility.

The Wellcome Sanger Institute is operated by Genome Research Limited, a charity registered in England with number 1021457 and a company registered in England with number 2742969, whose registered office is 215 Euston Road, London, NW1 2BE

- Lastly, we have updated the manuscript title to better reflect the biological insights from our study.

Together, these additions substantially enhance the robustness, depth, utility, and biological relevance of our manuscript.

Referees #1 and #2 (Remarks to the Author):

The authors Herranz-Ors et al. report the derivation and genomic and transcriptomic characterization of 256 tumor organoids from 5 different cancer types. Out of the established lines, the authors carried out genome-wide CRISPR screens in 98 lines with 2 sgRNA libraries. From the genetic screens, the authors found many previously known genetic decencies, as well as some new ones. Although this work provides limited new scientific findings, the resources will be very useful to the cancer research community (provided that it is deposited accessibly and that the annotation is improved). While the sequencing analyses were done thoroughly, there remains ambiguity in the results and interpretation.

We thank the referee for positively commenting on the resource value of our study.

Major comments:
1- To enhance the user experience and the impact of this resource, the authors should provide a detailed instruction manual that references for each tumor type / each established model all the available datasets, the used analysis code, corresponding SOPs, regulatory information & restrictions (MTA, controlled access data procedures).

We are passionate about the resource value of our biobank. We have included Supplementary Table 2, which includes key metadata for all organoids, including unique identifiers, availability of datasets, clinical data, and repository information. Additionally, a comprehensive overview of all models will be provided through the HCMI Explorer Suite - an interactive Shiny application developed by the HCMI.

The genomic (n=145), transcriptomic (n=140), and clinical data (n=256) for models reported in this study are currently freely available through the Cell Model Passports website (https://cellmodelpassports.sanger.ac.uk/), with the outstanding data being released prior to publication. All raw sequencing data are available through EGA (accession number: EGAD00001015469 - WGS, EGAD00001015468 - TGS, EGAD00001015470 - RNA). Organoid CRISPR screening data will be available via the website DepMap Miner https://dataminer.depmap.sanger.ac.uk/ prior to publication, which enables users to perform exploratory analysis on the data.

There are no restrictions on the use of the organoids or relevant regulatory information to disclose. SOPs for organoid derivation are described in the methods and detailed protocols are available through the protocols.io website with appropriate citations in the methods.

The Wellcome Sanger Institute is operated by Genome Research Limited, a charity registered in England with number 1021457 and a company registered in England with number 2742969, whose registered office is 215 Euston Road, London, NW1 2BE

2- The deposited code lacks a readme file, please annotate how the manuscript figures can be produced from the deposited raw data. The figshare deposit needs a citable and permanent DOI.

We have now deposited all scripts used for the main analyses in the Garnett Lab GitHub repository (https://github.com/Garnett-Lab/SangerOrganoidBiobank), which now includes a comprehensive README file detailing the workflow and software dependencies. In addition, we have provided an R Markdown document containing step-by-step instructions and all necessary data to reproduce the main figures. A private Figshare repository containing the raw and processed data, together with a table linking each dataset to the relevant analyses and figures, is available at: https://figshare.com/s/6534be83500d5716e2fc, and this will be made public with a permanent, citable DOI prior to publication.

3- Authors write in line 166 that they observed a "high concordance of somatic variants", which corresponds to lower than 70% shared SNVs for MSS samples. How much concordance would be observed across tumors within the same tumor type? Could the authors discuss the reasons for the observed low fractions of shared SNVs and Indels? (ext data fig 2d)

This question relates to the comparison of samples sequenced both at the Sanger Institute and by the HCMI. To provide greater context to the observed rate of shared SNVs and Indels, we analysed raw WGS data independently generated by HCMI and Sanger (n = 48 organoids) through a single pipeline to identify bias in processing steps that might contribute to differences in variant calling. In the revised manuscript, we have included Extended Data Fig. 2e panel showing (**Figure reproduced below**):

1. The proportion of shared variants does not increase substantially when a single pipeline is applied, even when using high-depth normal samples sequenced at Sanger as reference for HCMI data.
2. Most of the discrepancies arise from differences in Indel detection.

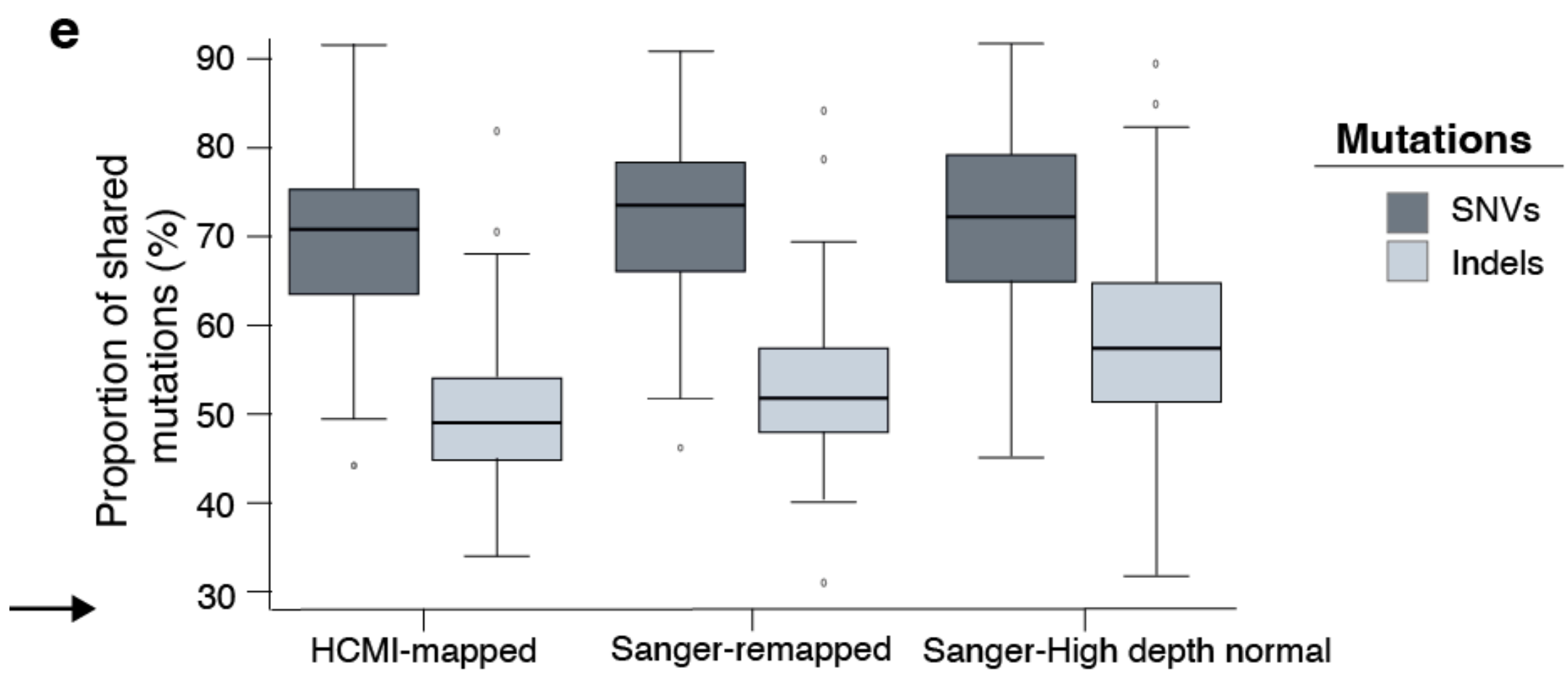

Figure: Comparison of Sanger and HCMI sequencing data. *Bar plot showing the proportion of SNVs and Indels shared between organoids sequenced at Sanger or HCMI, and analysed using either the HCMI or Sanger pipelines, or with high-depth blood samples from Sanger used as controls.*

Each box plot displays the median, first and third quartiles, with whiskers encompassing observations within 1.5 times the interquartile range.

The sequencing coverage at Sanger is higher versus HCMI (both in organoids and normal samples) (**see Figure below**). This likely explains the differences in Indel calling between the two studies.

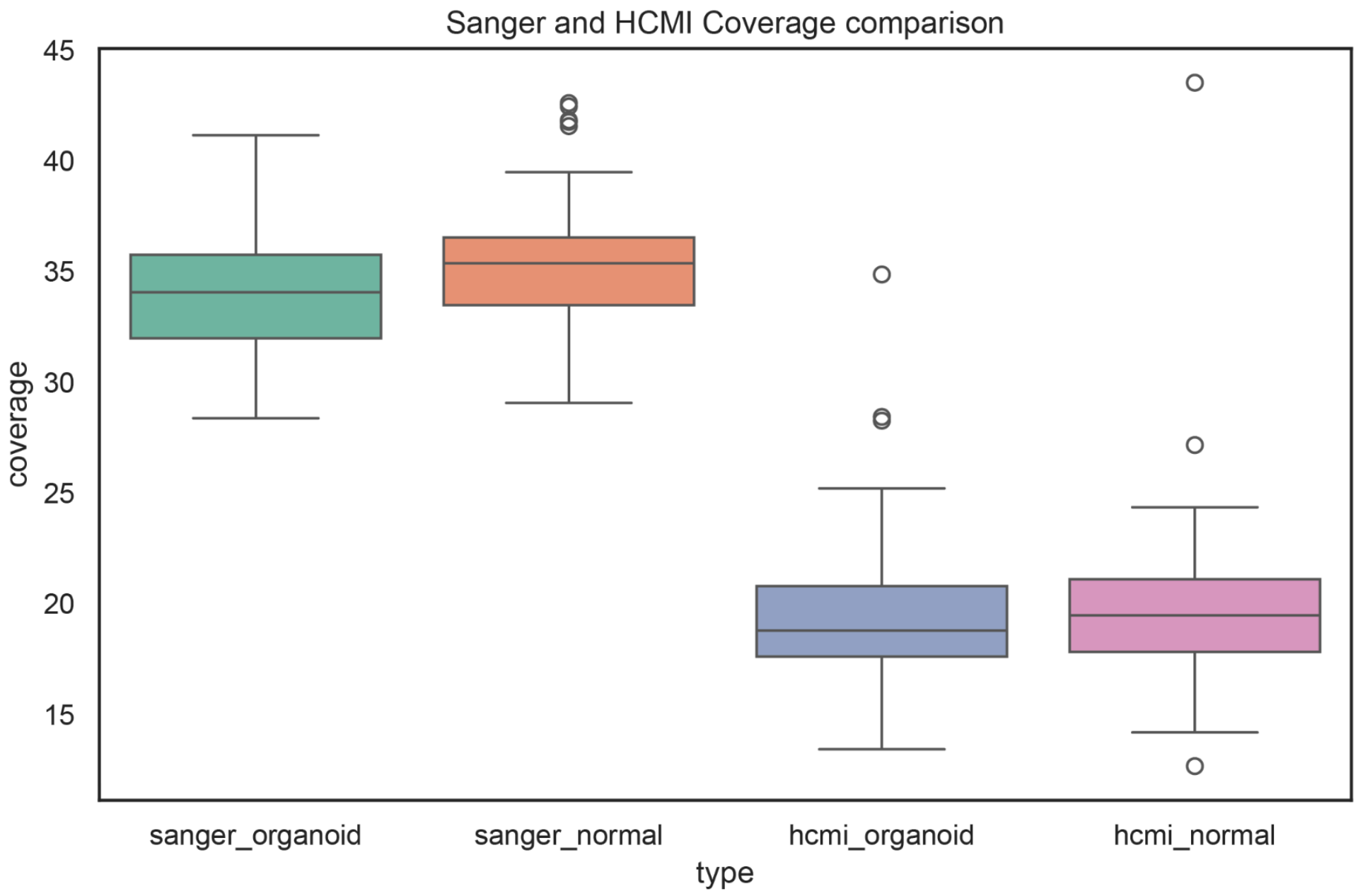

Figure: Sequence Coverage is Higher for Sanger than HCMI Data. *Box and whisker plots showing coverage for same organoids and normal samples sequenced by Sanger or HCMI (n = 48).*

In addition, using the same dataset, we calculated the proportion of shared SNVs and indels between matched organoid pairs and all possible pairwise combinations of unrelated organoid pairs within the same tumour type (**see Figure below**). These analyses showed minimal concordance (<1%) for unmatched samples, indicating that the ~70% overlap observed for matched organoid pairs represents a meaningful degree of fidelity.

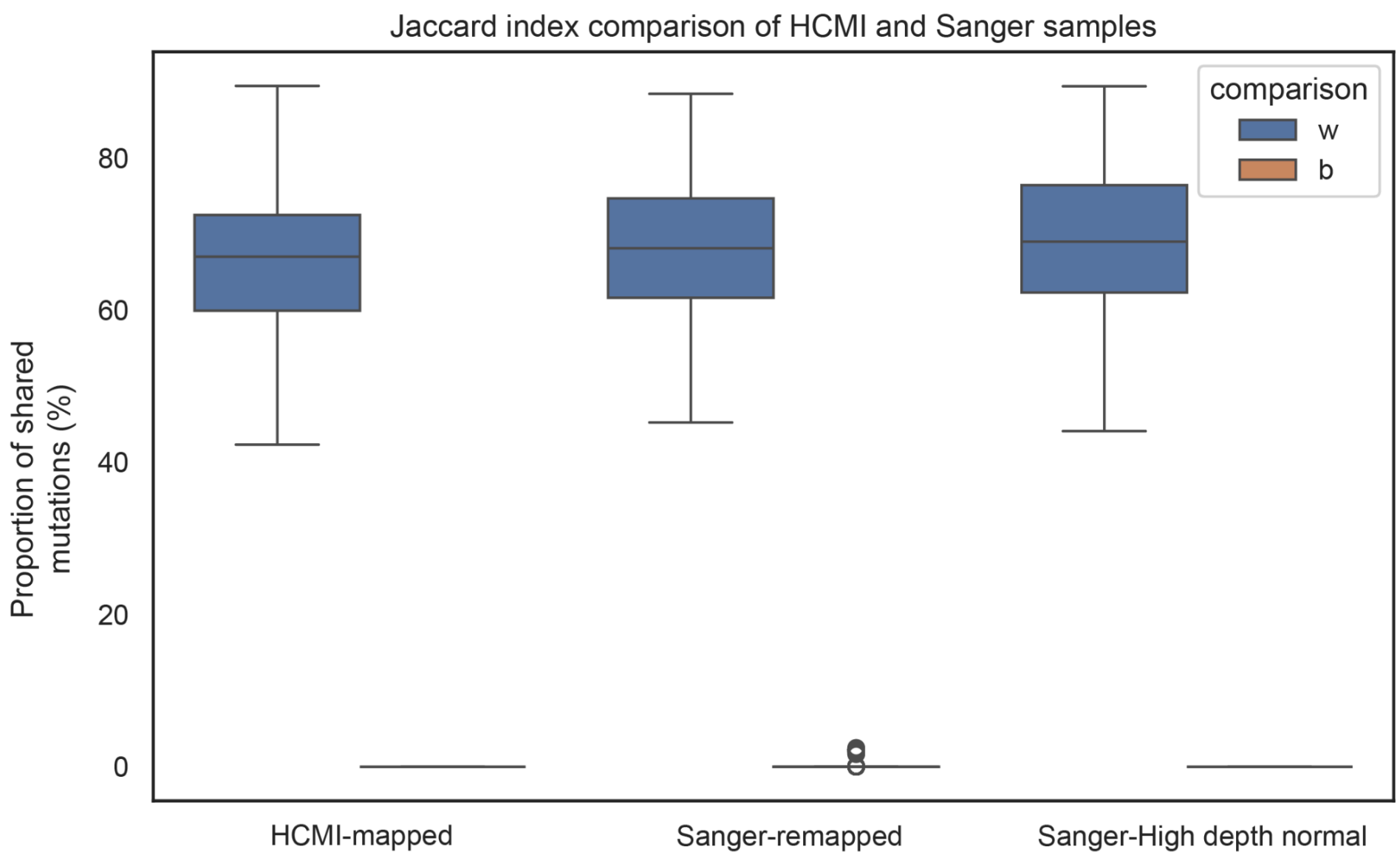

Figure: Comparison of SNV and Indel Overlap Between HCMI and Sanger Sequencing Data. Proportion of SNVs and Indels shared between matched-organoid pairs (w) and all possible pair-wise combinations of unrelated organoids (b).

4- Line 170: the authors mention an undisclosed commercial entity that will sell the established lines from Q4 2025. Authors have to ensure that distribution availability is in place by the publication of the manuscript. I think that a non-profit repository that ensures open, equitable access to patient-donated, taxpayer-funded models, would maximize public benefit and transparency. This approach would align with academia's mission to promote unrestricted sharing of research resources for societal advancement, rather than commercial profit.

As a Wellcome-funded initiative, which champions open and democratic science, a key objective is to make our organoid models widely accessible. To date, we have shared models across our Great Britain wide derivation network and with 15 academic laboratories, all at no cost.

To establish a long-term sustainable model for the biobank distribution, we have explored multiple strategies including non-profit biobanks (e.g. CancerTools.org), commercial vendors, and distribution directly from Sanger. The accessioning, maintenance, and long-term distribution of the biobank requires sustained financial investment and infrastructure that are not feasible at the Sanger Institute, or most other organisations. To address this, we have deposited 125 organoid models with the non-profit repository ATCC. Currently, 48 Sanger models are available for purchase (https://www.atcc.org/cell-products/collections-and-projects/human-cancer-models-initiative/hcmi-unmanufactured-models), with additional models scheduled for expansion guided by the community to prioritise model distribution (https://www.atcc.org/cell-products/collections-and-projects/human-cancer-models-initiative/hcmi-unmanufactured-models). In addition, the Life Science business of Merck KGaA (owners of brands such as Sigma-Aldrich and Millipore) recently acquired HUB Organoids and have licensed the Sanger organoid biobank for distribution. They will be distributing organoids to academic and commercial researchers with an anticipated launch date in Q1 2026. Both ATCC and Merck/HUB bring the financial resources, infrastructure, global distribution networks, and proven track record necessary to ensure consistent quality, wide access, and long-term sustainability of the biobank. We will issue a joint press release with Merck announcing this partnership upon public launch of the biobank.

The most up-to-date details on model availability are included in Supplementary Table 2. We believe our dual-repository strategy provides a long-term, robust and sustainable solution to maximise global access to the Sanger organoid biobank. During any interim periods when models are not yet available through repositories, we remain committed to supplying them directly to academic laboratories.

5- Metadata: line 178: at least 25 annotations: It's not about quantity but quality: Based on Supp table 2, survival and outcome data are not available for this cohort. This limits the usability; the authors should assemble these data types and add them to the metadata section.

We agree that clinical annotations of the biobank are a valuable aspect of our study. We provide detailed demographic (e.g. age, sex), patient-specific (e.g. smoking status, alcohol consumption), and clinical (e.g. tumour staging, location, prior treatment) data, including curated cancer-type specific clinical features of particular clinical importance. We respectively argue they offer significant value, and are not available for the majority of

existing cell models, representing a significant value proposition and valuable tool for the community.

At the outset of our study, we worked with our clinical co-authors to assess the feasibility and utility of systematically acquiring patient survival and outcome data. Our clinical collaborators advised that survival data, in the absence of a comprehensive longitudinal clinical history, would have limited utility, as it does not account for co-morbidities, treatment adherence, lifestyle factors or multiple lines of treatment. Tracking the clinical history of patients—across successive treatments, over several years, and at multiple clinical sites—was deemed infeasible due to the complexity and substantial long-term resources required to sustain this effort.

6- Do organoids that passed quality control (QC) differ in mutational profiles or driver mutations from those that failed QC? The number of days until the first passage varied substantially; could genetic differences underlie this variability?

To address this question, we compared the frequency of genomic events in our organoid biobank with those observed in primary tumour samples from TCGA, and found similar patterns. This provides indirect evidence for minimal bias in the derivation of QC-passed organoids (Extended Data Fig. 9, Supplementary Table 4).

Although we did not routinely sequence QC-failed organoids due to cost, we have generated sequencing data for a subset. Ten organoids failed QC due to low tumour purity, which hindered reliable detection of cancer driver genes in the WGS data (Extended Data Fig. 1a; labeled “no cancer drivers found” in Supplementary Table 1). For six of these models, we generated targeted sequencing data with high coverage (~800X), both at the time of banking and after freeze–thaw QC. In two cases, no cancer driver mutations were detected. In a third case, the FAT1 gene harboured a mutation with variant allele frequency (VAF) < 0.05. Two additional samples carried KDM6A mutations with VAFs slightly above 0.05 at banking, which fell below this threshold after freeze–thaw QC. One final sample carried mutations in two genes: KMT2C (VAF < 0.05) and GNAS, which had a VAF of 0.4 at banking that dropped to 0.08 post freeze–thaw.

We have also investigated whether specific clinical or genomic features were associated with organoid derivation parameters, including the number of days to first passage. Using the clinical and genomic features curated for the CRISPR biomarker analysis, we applied the same statistical framework to test for associations with metrics including tumour fragment size, passages until banking, days until banking, and days until freeze–thaw QC. As reported in the original manuscript, tumour stage positively correlated with tumour size. Beyond this, we identified four additional associations with genomic data; however, these were weak, often driven by outliers, and lacked biological plausibility (**see Figure below**). Statistically significant associations are provided below for completeness.

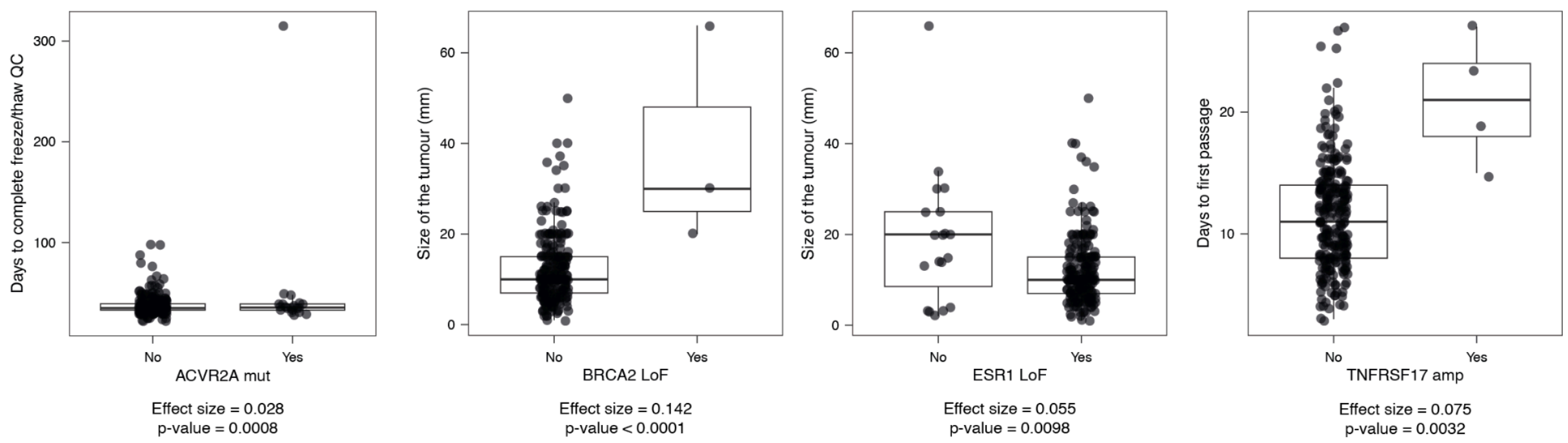

__Figure: Genomic Features Associated with Organoid Derivation.__ Features associated with derivation parameters include mutations (mut), gene loss of function (loF), and amplification (amp). A linear regression model was applied and only associations with a p-adj-value < 0.05 and with an effect size within the top absolute 10% were considered significant.

Overall, we did not find evidence for genomic features significantly influencing organoid derivation success or other derivation parameters. However, as shown in the accompanying HCMI study, gene set enrichment analysis (GSEA) indicates that failed derivations were enriched for DNA repair and cell-cycle–associated programs, whereas successful models showed lower activity of these pathways, providing a potential explanation for failures.

7- The authors conducted CRISPR screens on 98 organoid lines, each subjected to three rounds of lentiviral transduction (Cas9 transduction, sgRNA testing, genome-wide library). It is unclear whether validations were performed using freshly prepared batches of organoids. Additionally, the manuscript lacks validation of hits using individual sgRNAs. The authors should clarify these points and ideally perform validations with distinct batches of organoids and specific sgRNAs.

CRISPR screens were performed on organoids that underwent a single round of Cas9 transduction prior to screening. Apologies if this was unclear and this has been clarified in the Methods.

Screens were performed with a pool of Cas9-expressing organoids to reduce the risk of bias associated with selection of a single Cas9-expressing organoid clone that could effect screening results.

Additionally, we established three independent Cas9-expressing organoid lines, distinct from those used in the initial screen, and re-screened them with the MinLibCas9 library to assess the reproducibility of our platform across batches. There was a high correlation between normalized log fold changes (LFCs) processed with CRISPRcleanR between original and new data, demonstrating strong consistency and the absence of significant batch effects across independently transduced batches of organoid lines (**see Figure below**).

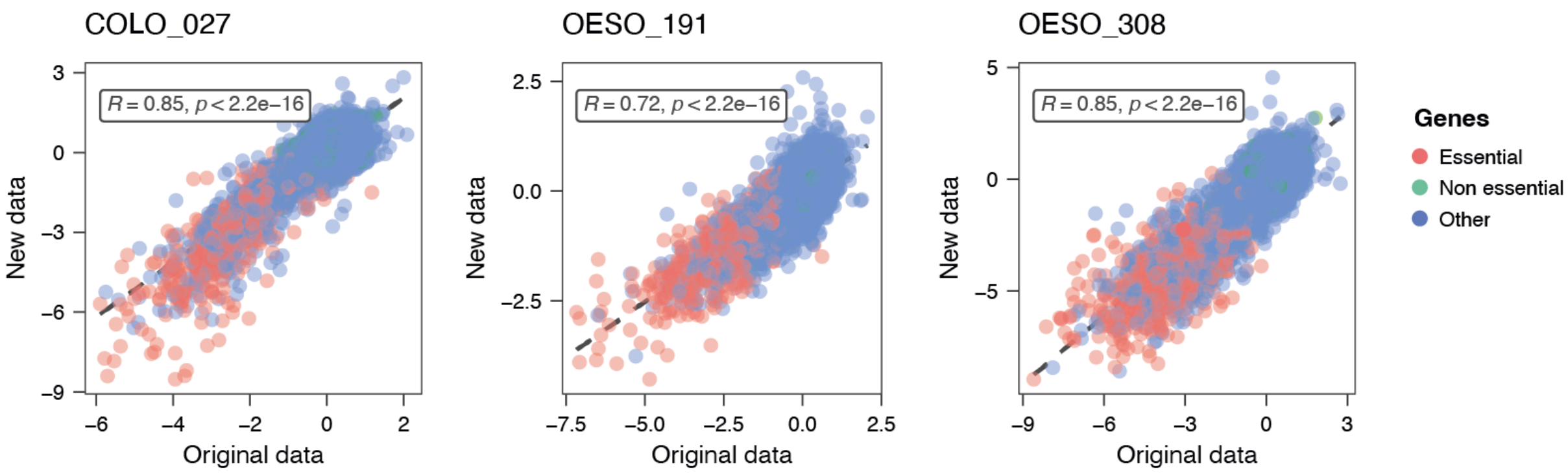

__Figure: High Reproducibility of CRISPR/Cas9 Screens in Two Batches of Cas9-expressing Organoids.__ Comparison of new screening data versus original data in two independent batches of Cas9-expressing organoids. Gene LFC values are shown for screens performed in 3 separate organoid cultures.

The Wellcome Sanger Institute is operated by Genome Research Limited, a charity registered in England with number 1021457 and a company registered in England with number 2742969, whose registered office is 215 Euston Road, London, NW1 2BE

We have additionally taken multiple approaches to validate our CRISPR screen results. Specifically,

1. Confirmatory drug sensitivity testing were performed in parental organoids (not Cas-9 transduced) based on results from CRISPR screens that serve as independent validation of results (Figure 5d and 5e; including 8 inhibitors in two organoids (two each of KRAS, DNMT1, proteasome inhibitors).

2. The correspondence of MDM2 dependency using Nutlin3a sensitivity in 49 COAD/READ organoids (Extended Data Fig. 16c) supports our CRISPR dependency data.
.
3. Additionally, we have tested the same drugs on Cas9-transduced and parental organoids **(see Figure below)**:

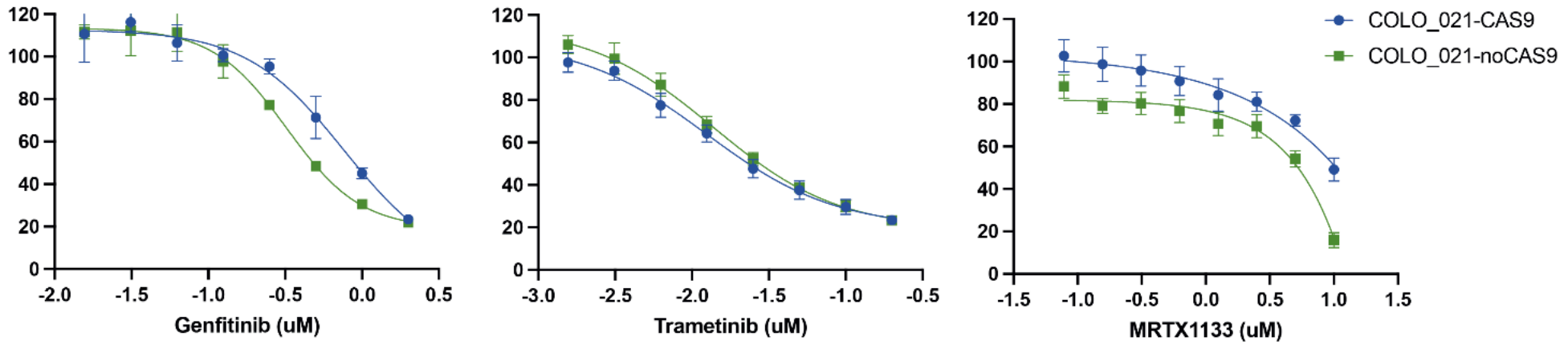

Figure: Consistent Dose Response Curve in Parental and Cas9-expressing Organoid. *Representative dose response curves for 3 drugs in Cas9-expressing and parental (noCas9) organoid COLO_021. Data are the mean and standard deviation of triplicate experiments and representative of duplicate experiments.*

8- The organoid pair HCM-SANG-0299-C15-A and -B, included in the screen, exhibited differences in the number of depleted genes. Could the authors provide insights into how treatment history or disease progression might influence genetic dependencies?

The two organoid models HCM-SANG-0299-C15-A and -B were derived from the same patient, but before and after chemotherapy, over a period of ~1 year. Genomic profiling reveals that these organoids share approximately 50% of somatic SNVs and indels (Extended Data Fig. 6a), and notably, the post-treatment organoid lacks a MYC amplification present in the pre-treatment organoid. Our data suggest the potential outgrowth of a drug-resistant subclone following therapy, consistent with a shift in the tumor's clonal architecture and selective pressures exerted by treatment (PMID: 40664650). Similar patterns of treatment-induced genomic remodeling, such as the loss of HER2 amplification after neoadjuvant therapy, have been described in HER+ breast cancers, with a negative impact on the prognosis and risk of disease recurrence (PMID: 40112659). Given this substantial genomic divergence, it is expected that genetic dependencies identified through CRISPR screening could differ between the two models.

9- The authors report increased genetic dependency on MDM2 in wild-type p53 and KrasG12D/Kras mutant COAD/READ organoids. However, the subsequent validation with Nutlin-3a treatment is incomplete and insufficiently convincing, given that many data points are missing. Only approximately half of the organoids identified in the screen as depleted for MDM2 dependency responded to Nutlin-3a. The authors should carry out Nutlin-3a treatment or MDM2 knockout experiments in the missing data points to strengthen the validation.

In the original manuscript, we performed Nutlin-3a sensitivity experiments in 42 of 55 COAD/READ organoids with accompanying CRISPR screening data. As suggested, we have performed Nutlin-3a sensitivity assays in 7 more COAD/READ organoids. In the Extended Data Fig.16c of the revised manuscript, we now show only models with both CRISPR and Nutlin-3a sensitivity data to improve clarity (n = 49). Our data show overall good concordance between MDM2-dependency and Nutlin-3a sensitivity, with some variation across our diverse cohort of models. There is a statistically significant difference in both MDM2-dependency (p-value = 0.037) and Nultin-3a pharmacological inhibition (p-value = 0.002) in *TP53* wild-type versus *TP53*-mutant organoids (**see Figure below**), supporting our conclusion.

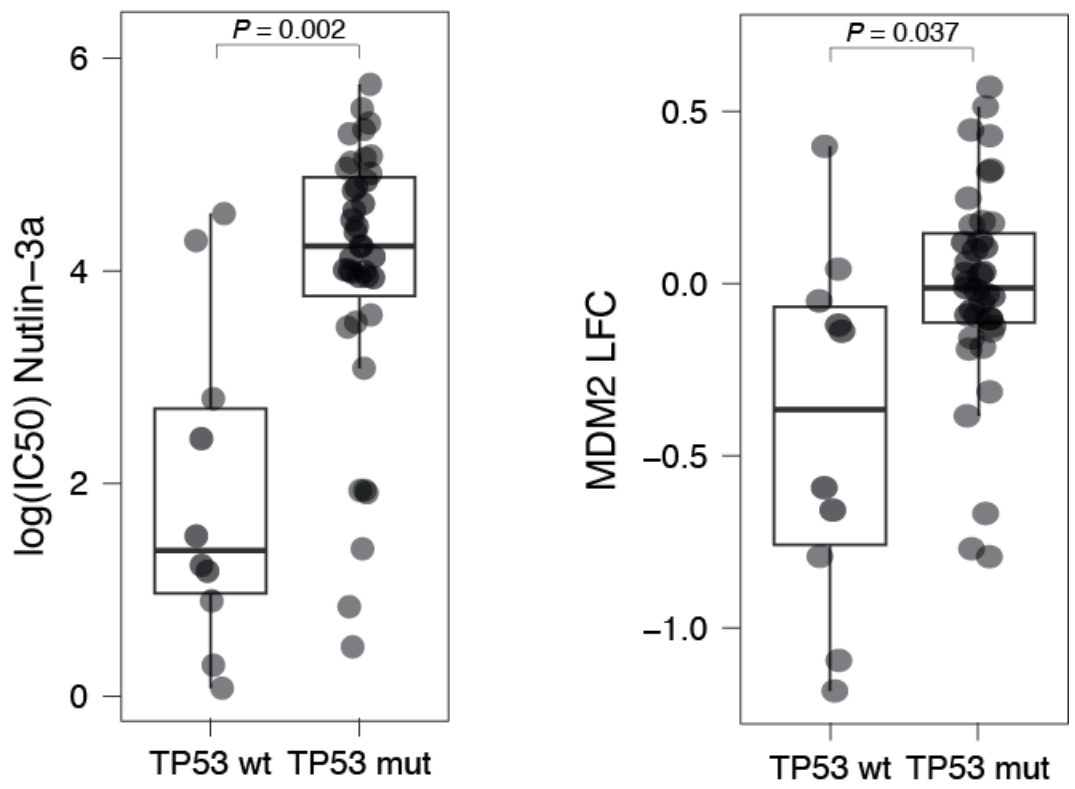

Figure: Sensitivity to Nutlin-3a and dependency to MDM2 in TP53 mutant colorectal COAD/READ. *Difference in Nutlin-3a sensitivity and MDM2 dependency between TP53 wild-type (wt) and mutant COAD/READ organoids. T-test applied.*

In addition, we have investigated the effect of specific *TP53* mutations on both Nutlin-3a response and MDM2 dependency to understand if this explains variable results, but found no significant differences across *TP53* variants, suggesting this does not explain the variability observed.

Finally, consistent with our broader analyses, KRASG12D mutant organoids exhibited a stronger dependency on MDM2 compared to organoids harboring other KRAS mutations or KRAS wild-type models (p-values = 0.001 and 0.002, respectively), and showed a trend toward greater Nutlin-3a sensitivity (**see Figure below**). All 4 dependent KRASG12D mutant organoids were *TP53* wild-type (one KRASG12D/TP53 mutant organoid was insensitive), and this has been stated in the manuscript text.

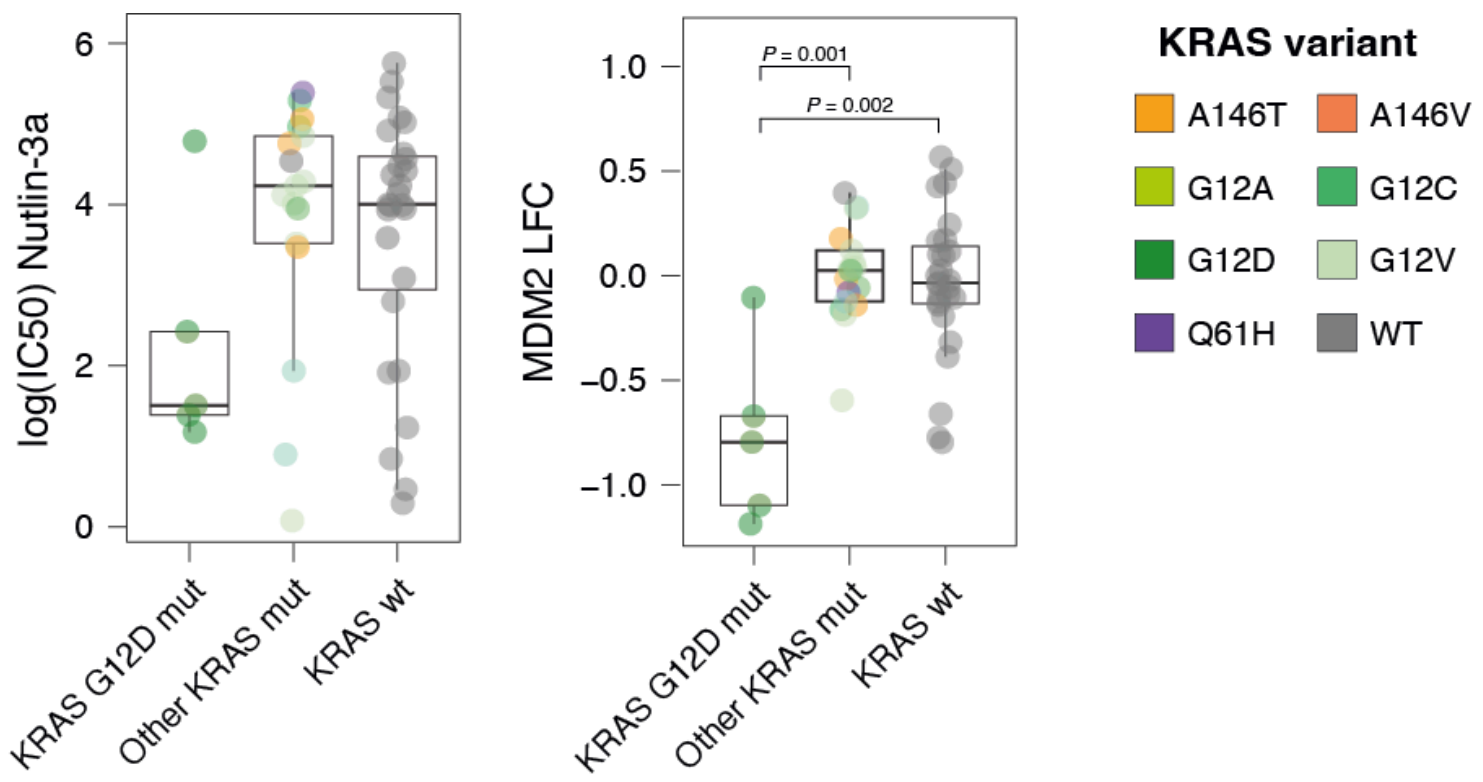

Figure: Sensitivity to Nutlin-3a and dependency to MDM2 in KRASG12D colorectal COAD/READ. *Difference in Nutlin-3a sensitivity and MDM2 dependency between KRASG12D mutants, other KRAS mutants or KRAS wild-type COAD/READ organoids. Wilcox-test applied.*

10- The statement that "KRAS wild-type organoids have increased dependency on EGFR, ERBB2, and PTPN11" currently lacks statistical analysis or experimental validation. The authors should provide appropriate statistical tests or additional experimental evidence supporting this claim.

We have updated Figure 5 in the revised manuscript and included the relevant statistical tests for this observation and new data for EGFR and ERBB2 inhibitor sensitivity in KRAS-mutant and wild-type organoid models to support this finding.

11- Could the authors specify clearly the genetic background of the organoids labeled as "WT"? Are these organoids wild-type for KRAS but mutated in other components of the EGFR-MAPK pathway? Additionally, could the authors comment on why Gefitinib appears ineffective in WT cells, whereas Afatinib alone is effective in KrasG12D-mutant organoids, contrasting previous findings (e.g., PMID: 27845624)?

We thank the referee for raising these important points. In the new version of the manuscript, we have changed Fig. 5a panel and its associated Extended Data Fig.17.

Now, in Fig. 5a, we show KRAS, EGFR, and PTPN11 LFCs in organoids grouped by KRAS status (KRASG12 mutant, KRAS other mutant or KRAS wild-type). In addition, we indicate if the models have any other alterations in EGFR-MAPK pathway genes such as BRAF/MAP2K1 mutations, EGFR/ERBB2 amplifications or PTEN deletions, clarifying the interpretation of results.

Our updated analyses are consistent with findings from the referenced study (PMID: 27845624). Specifically, RAS wild-type organoids had increased sensitivity to EGFR/ERBB2 inhibitors such as gefitinib and afatinib, compared to KRAS mutant organoids. However, responses were heterogeneous, and in some cases, resistance was associated with additional mutations in the EGFR-MAPK pathway (as previously described. e.g. PMID: 26416732).

Furthermore, we have demonstrated increased dependency on EGFR knockout and greater sensitivity to afatinib or erlotinib of KRASG12X mutants compared to organoids with other KRAS mutant alleles—though still less sensitive than KRAS wild-type cells. These findings align with emerging evidence that distinct KRAS mutant alleles have different biological consequences (PMID: 26985062). Notably, the KRASG12X mutant alleles exhibit higher

The Wellcome Sanger Institute is operated by Genome Research Limited, a charity registered in England with number 1021457 and a company registered in England with number 2742969, whose registered office is 215 Euston Road, London, NW1 2BE

intrinsic GTPase activity (PMID: 26037647), which may lead to a greater dependency on upstream RTK/EGFR signalling than seen in other alleles such as Q61 (PMID: 40294008), shown in Fig. 5c with the withdrawal of EGF from the media.

In addressing the referee's comments, we believe these additional analyses not only strengthen our study but also contribute substantively to the growing body of evidence that functional heterogeneity among RAS-pathway alterations has important implications for therapeutic response. These insights represent a significant advance and further underscore the value of our large, diverse biobank in capturing the spectrum of RAS–MAPK pathway mutations and their biological effects.

Minor comments:
1- Referring to "methods", specify exact location.

We have specified the exact location in the methods section as requested.

2- Numbers of successfully established STAD models seem to be discrepant: Figure 1b reports 6 models passing QC, Ext Data Fig 1b reports 3. Which one is correct?

We have clarified this discrepancy. Extended Data Figure 1b shows only models that have data on the preservation method (fresh vs. frozen). We have made this clear in the figure legend.

3- Please improve the resolution of Fig 1a.

We have modified and improved the resolution of Figure 1a.

4- Based on the data in Supp Figure 2 it remains unclear to me which models were derived from metastases and which were derived from primary tumors. This should be better annotated.

We have updated the annotation to make this clear in Supplementary Table 2.

5- Why was WGS only conducted on 249 of the 256 organoid lines? Will the author add the remaining WGS data?

We have updated the manuscript to include WGS data for all 256 organoids.

6- Drug sensitivity and cell viability assays utilized fluorescent readouts. Could the authors clarify how this method was adapted for organoid cultures? Specifically, were organoids dissociated before fluorescence measurement, or was another approach employed?

As described in our methods paper (PMID: 27628132) and consistent with previously published protocols (PMID: 27845624), organoids were first dissociated into single cells, resuspended in organoid culture medium, and then seeded into 96-well plates pre-coated with a 50:50 mixture of medium and ECM. Cell viability was quantified 72 hours post-treatment using CellTiter-Glo 2.0 (Promega). We avoided embedding organoids within the ECM matrix, as this can result in variable assay performance.

<u>Referee #3 (Remarks to the Author):</u>

Garnett's team describes an establishment of a patient-derived organoid biobank that encompasses colorectal, esophageal, ovarian, pancreatic and gastric cancer. The biobank is equipped with rich clinical, genomic and transcriptomic information, and genome-wide dropout CRISPR screening was conducted in a subset of organoid lines, leading to the discovery of some new fitness genes and drug targets. The authors state that all organoid lines will be available through a commercial vendor, and along with the accompanying papers, this work will provide a great resource for basic and preclinical research communities. While the effort spent to derive and analyze the organoids is very impressive, establishment and collection of organoids from major cancer types has been repeatedly reported previously, and insights from this work may not transcend our current knowledge from the compilation of prior studies on patient-derived organoid biobanks in the absence of some strong, new and compelling data that strongly drives forward the cancer research field. Below are my specific comments.

We thank the referee for their positive comments on the resource value of our study and we appreciate their constructive comments on how to improve our manuscript.

We believe our biobank is uniquely distinguished by both its scale and the depth of genomic and functional characterisation compared to previous efforts. The two next largest academic resources included 115 and 96 organoid models, respectively (PMID: 35719949; PMID: 31761724), and lack comprehensive genomic data (**see figure for comparison of biobanks**). Furthermore, for most studies, it is unclear if these are long-term renewable cultures and whether they are publicly available with appropriate consent, limiting their utility to the research community.

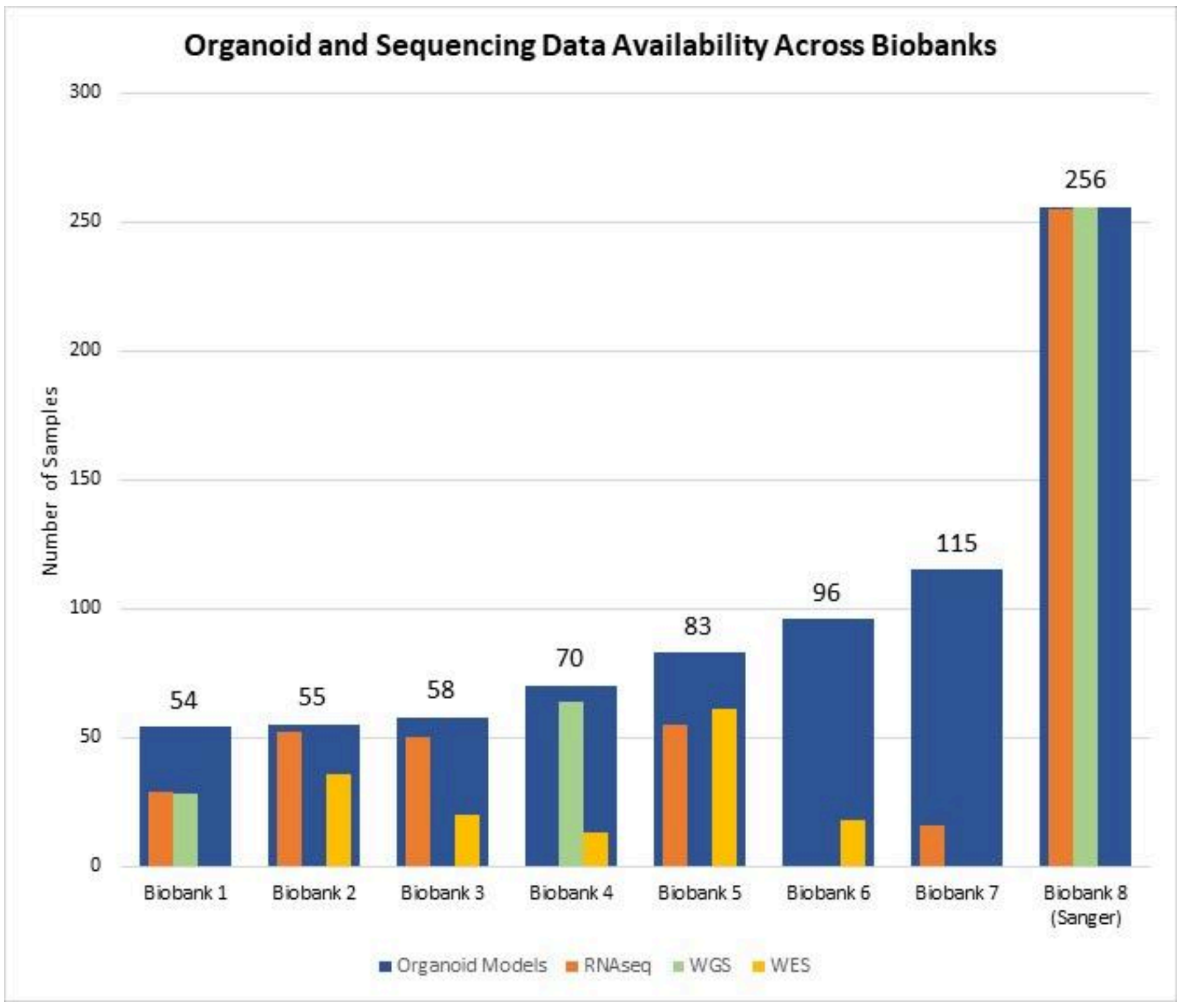

Figure: Relative scale and depth of genomic and transcriptomic characterisation of organoid biobanks. *Comparison of this study (Biobank 8) to several published academic biobanks directly derived from patient samples at the level of number of models and genomic/transcriptomic data available. The biobanks referenced in this figure are PMID Biobank 1: 32161258, PMID Biobank 2:*

The Wellcome Sanger Institute is operated by Genome Research Limited, a charity registered in England with number 1021457 and a company registered in England with number 2742969, whose registered office is 215 Euston Road, London, NW1 2BE

27212702, PMID Biobank 3: 36058001, PMID Biobank 4: 29472484, PMID Biobank 5: 34083237, PMID Biobank 6: 31761724, PMID Biobank 7: 35719949.

In contrast, our biobank is more than twice the size of previous academic reports, and delivers a renewable, well-characterised biobank with broad consent for model and data sharing, and is being distributed through third-party repositories. Furthermore, the depth of genomic and transcriptomic characterisation of our models, which includes SNVs, Indels, CNVs, mutational signatures, whole genome duplications, chromothripsis, clonal fraction, structural variants, molecular sub-types, and HRD signatures far exceeds previous studies.

Our study is also the first report of a genome-scale organoid dependency map, establishing not only a valuable and accessible resource for the field but also a robust proof-of-concept for using functional genomics in patient-derived models. Finally, in our revised manuscript, we interrogate genetic and pharmacological dependencies on the EGFR-RAS-MAPK pathway, demonstrating KRAS mutant allele-specific effects on pathway dependency and the requirement for upstream signalling, with potential implications for patient stratification to KRAS-targeted therapies now in the clinic.

Together, we believe our revised manuscript represents a major advance over previous efforts in both resource generation and the application of organoid models for discovery science.

Major comments:
1- The success rate of stable organoid culture appears to be exceeding low especially for colorectal cancer organoids compared to prior studies (van de Wetering et al., Cell 2015; Fujii et al., Cell Stem Cell 2016; Schütte et al., Nature Communications 2017). What do the authors think does this difference and the low take rake arise from?. The low establishment rate can result in a potential bias of the biobank toward some “easy-to-establish” cancer types or subtypes. Were there any clinical or perhaps technical variables associated with establishment failure? For instance, with respect to technical experience, does the establishment rate differ between the first and second halves of the study period? Was the same establishment procedure used throughout the study period, or was it modified to improve establishment efficiency?

We agree that these are important points and address them in turn. While we initially shared the referee’s concerns about the take rate, we now believe that the differences observed largely reflect variations in study design and quality control (QC) criteria. Unlike some studies (e.g., PMID: 29472484), we did not pre-select tumour specimens for derivation based on tumour size, stage, site, or cellularity, which likely negatively impacted our take rate. Our QC thresholds were deliberately stringent to ensure the generation of high-quality, renewable models. Each culture was required to yield a minimum of 25 cryovials (1×10^6 cells per vial), demonstrate post-thaw viability, and sustain growth for at least four additional passages. In contrast, many published studies do not define or report their QC criteria, or focus on short-term cultures (e.g. PMID: 39305899), making direct comparisons of derivation success rates challenging.

In our dataset, the most common reasons for model failure were the development of non-renewable short-term cultures (labelled ‘short term culture’) and insufficient tumour cellularity at the time of derivation (labeled ‘lack of cells’; Extended Data Figure 1a). When we account for these factors—by including short-term cultures and excluding samples with inadequate starting material—our overall success rate is 65%, in line with rates reported in previous studies.

Importantly, our observed take rate is consistent with those reported by another study with similar biobanking criteria (24% take rate; PMID: 34320344).

We have evidence that our biobank is not biased toward "easy-to-establish" cancer subtypes. We have shown that the genomic profiles of our models broadly reflect the TCGA tumour landscape (Extended Data Fig. 9). This includes for colorectal cancer, where we observe balanced representation across CRIS and CMS gene expression molecular subtypes (Extended Data Fig. 11). That said, we acknowledge that some cancer types are more inherently challenging to culture—for instance, ovarian cancer exhibits a lower establishment rate than colorectal cancer in our dataset—and we have clarified this in the revised manuscript by reporting relative success rates for each tumour type.

We would have liked to compare clinical data between tumors that failed or succeeded in organoid derivation; however, because clinical data were collected retrospectively only after a successful derivation, this analysis was not feasible. Instead, we evaluated clinical features in relation to derivation metrics among successfully established organoids. The only statistically significant association observed was that the size of the initial tumor sample correlated with tumor stage (Extended Data Fig. 1h).

In addition, we present here data on the temporal progression of our establishment success rates and highlight major protocol modifications (**see Figure below**). While there is some evidence for improvement over time for COAD/READ derivations, for most tumour types the success rate was consistent over time. However, there are multiple confounding factors over our 8 year derivation effort that make interpretation difficult, including modifications to protocols, improvements to media components, changes to vendors, turnover of staff at Sanger and clinical sites, and the impact of the Covid-19 pandemic.

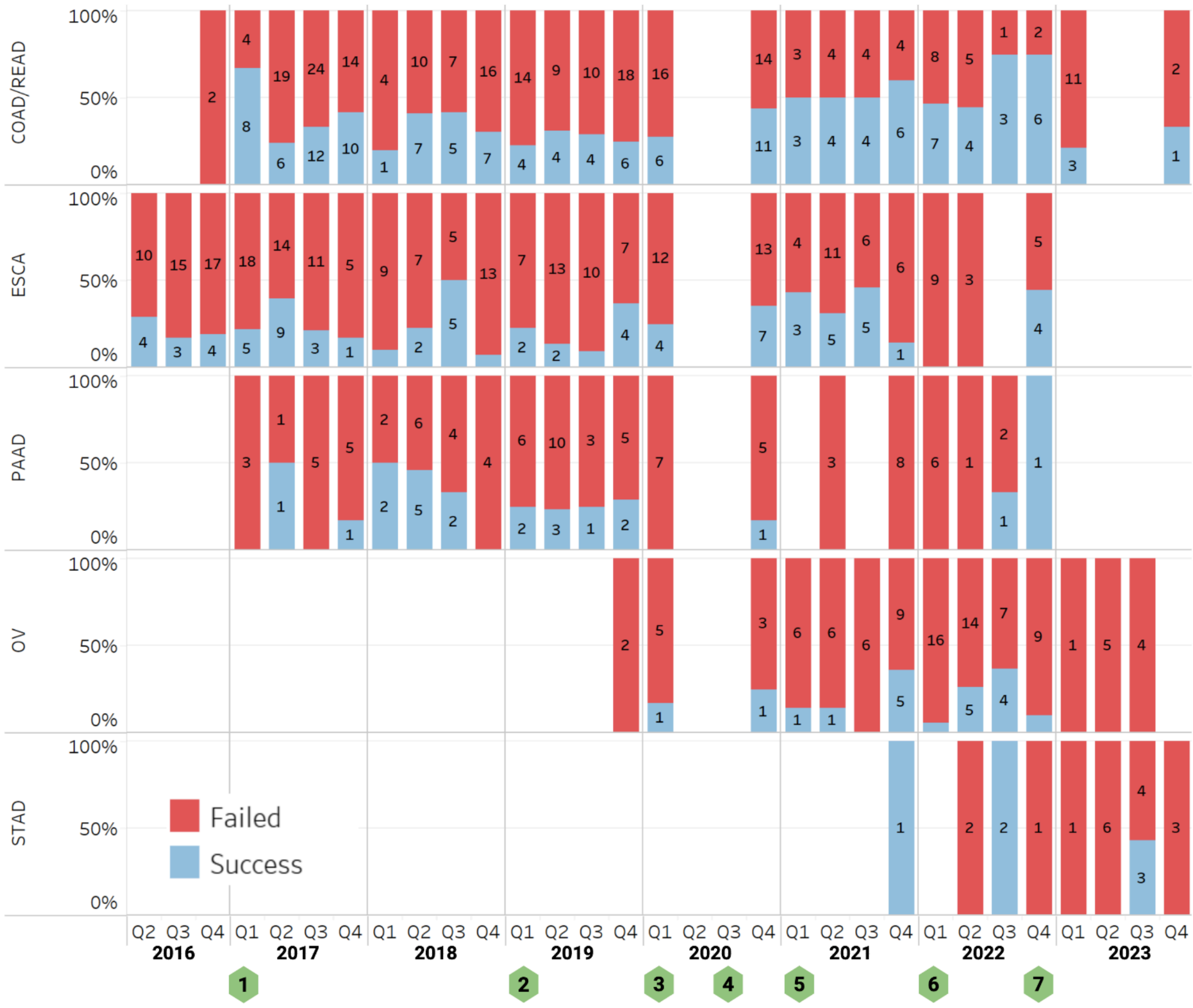

1. **Feb 2017** - Addition of N-2 Max Media Supplement (Cat #: AR009)
2. **Jan 2019** - Supplier of recombinant human EGF changed from Gibco (Cat #: PHG0313) to Peprotech (Cat #: AF-100-15-1000)
3. **Mar 2020** - Wnt surrogate (Cat #: N001) started to replace Wnt conditioned media (produced in-house at Sanger Institute)
4. **Jul 2020** - RSPO3-FC (Cat #: R001) started to replace R-Spondin conditioned media (produced in-house at Sanger Institute)
5. **Mar 2021** - Switched from N-2 Max Media Supplement (Cat #: AR009) to CTS N-2 Supplement (Cat #: A1370701). ***Ovarian*** media updated: BMP2 and Acetic Acid added (Cat #: PHC7145); Wnt conditioned media, Human Noggin and R-Spondin 1 conditioned media removed
6. **Jan 2022** - Noggin supplier changed from Peprotech (Cat #: 120-10C-500) to U-Protein Express (Cat #: N002)
7. **Oct - Nov 2022 -** Noggin supplier reverts back from U-Protein Express (Cat #: N002) to Peprotech (Cat #: 120-10C-500)

Figure: Derivation Success Rates Over Project Duration. *Success rates are shown for each tumour type separately for each 3 month period. Major changes to derivation protocols are annotated.*

2- The authors performed CRISPR screening in a large panel of patient-derived cancer organoids, which is impressive. However, dependency genes they found may be lineage-specific, but not cancer-specific, making them not strong enough as drug target candidates. One of the major strengths of organoid technology is the ability to stably expand normal epithelial tissues. As the goal of CRISPR screening in this study was to identify cancer vulnerabilities, the paper strongly merits from adding and analyzing CRISPR screening of normal organoids and perhaps discovery of cancer-specific genetic dependency.

We recognise the difficulty that the reviewer raises as a challenge for the community. Our biobank does not include normal organoid models. Although we are aware that individual

The Wellcome Sanger Institute is operated by Genome Research Limited, a charity registered in England with number 1021457 and a company registered in England with number 2742969, whose registered office is 215 Euston Road, London, NW1 2BE

and sequential targeted gene knockouts using CRISPR/Cas9 technology, specifically in organoid cultures from epithelial stem cells have been successfully performed (PMID:28912133, 37772705, 38857990), CRISPR screens in untransformed human organoids have (to the best of our knowledge) not been previously reported. We additionally note that to comprehensively address the reviewer's comment would require tissue/lineage-matched, and ideally patient-matched normal organoids, which would be exceptionally challenging. Instead, we have developed a new analytical framework, based on genetic constraint in human populations, that we believe substantially addresses the reviewers comment and has the benefit of being derived from an independent, orthogonal human data source.

Our original approach focused on identifying differential genetic dependencies across cancer organoid models, while excluding pan-fitness/essential genes and tissue-specific core fitness genes, consistent with the Dependency Map strategy in cell lines (e.g., PMID: 30971826; PMID: 28753430). We have now additionally annotated the genes from our differential dependency analysis using genetic constraint categories based on the LOEUF metric. This is a continuous measure of a genes' intolerance to loss-of-function variants, derived from gnomAD based on the analysis of 140,000 humans, and accessed via the Open Targets platform (PMID: 27535533 and 32461654). Specifically, genes under high levels of genetic constraint are more likely to be involved in human disease and essential for human physiology, and therefore may be associated with increased essentiality. We believe this information can help to identify generally essential and lineage-essential genes. Indeed, we find that known essential genes are enriched for genes with high genetic constraint (revised Fig. 4e). This approach additionally has the advantage of being directly inferred from orthogonal and population scale human genetic data (rather than cell models). We provide this annotation as an additional layer of information to aid in evaluating the therapeutic potential of newly identified gene dependencies from our dataset.

To extend functional context, we also incorporated mouse knockout phenotype data and tissue specificity of expression into the annotation of organoid-specific core fitness genes (Extended Data Fig. 15). Using these layers of annotation helped to distinguish between potential new cancer core fitness genes (low genetic constraint, low mouse knockout impact) versus potential new core fitness/essential genes (high genetic constraint, severe mouse knockout impact). Collectively, these annotations add valuable context to prioritizing targets. We have additionally included in the manuscript a discussion of distinguishing lineage-specific from cancer-specific dependencies. We thank the reviewer for this helpful comment.

3- Also, according to Methods, the authors used 1.25 or 3.3 x 10^7 cells with MOI = 0.3 for genome-wide CRISPR screening. How were these cell numbers determined? I consider this scale is not large enough to achieve sufficient coverage and depth of the guides, and to maintain the library diversity in every model. Not all cells can form organoids or spheroids after single cell dissociation, and the colony-formation efficiency can significantly differ among the models. For instance, if you use 1.25 x 10^7 cells with an MOI of 0.3 and Yusa v.1.1 sgRNA library with an library size of 10^5, assuming a colony formation efficiency of 0.2 (which is a fair value for normal organoids but can be lower in cancer organoids), a rough calculation shows that you only get 7.5 clones per sgRNA. The authors appear to have calculated fold change values based on the read counts from plasmids and post-screen models, but were all the guide RNAs detected at early time points after infection?

We appreciate the reviewer's technical questions, which highlight the complexity of systematically screening in 3D organoids and the technical challenges we addressed to

overcome these limitations. Our study establishes a robust framework for CRISPR screening in organoids, underscoring both its enabling value and the importance of the datasets generated. In response, we have also generated new experimental data to further substantiate the quality of our CRISPR screens.

Firstly, we thank the referee for the opportunity to clarify our protocol here and in the methods section of the manuscript. For the CRISPR screens, we used 3.3 × 10^7 cells per replicate for the Yusa v1.1 library and 1.25 × 10^7 cells per replicate for the MiniLib, targeting 100x library coverage with a multiplicity of infection (MOI) of 0.3.

We established a live-cell imaging approach to directly determine organoid colony formation efficiency at the single-cell level. Three organoids were labeled with a red dye, seeded as single cells in 5 µl BME drops in 96-well plates, and live-cell imaged using an IncuCyte. Images were acquired every 8 hours from the same focal plane, allowing us to track single-cell growth over time. The red dye was progressively lost as cells proliferated, enabling us to distinguish between single cells (retaining the red dye, marked as blue dots) and those that had formed organoids (green dots). For each organoid line, six wells were manually counted at four different time points. Representative images at days 1 and 4 are shown below. Across three models tested, we confirmed that over 75% of single cells seeded under these conditions successfully formed organoids (**see Figure below**). Thus, our colony formation efficiency is significantly higher than 20%, and likely close to our target of 100x coverage per replicate.

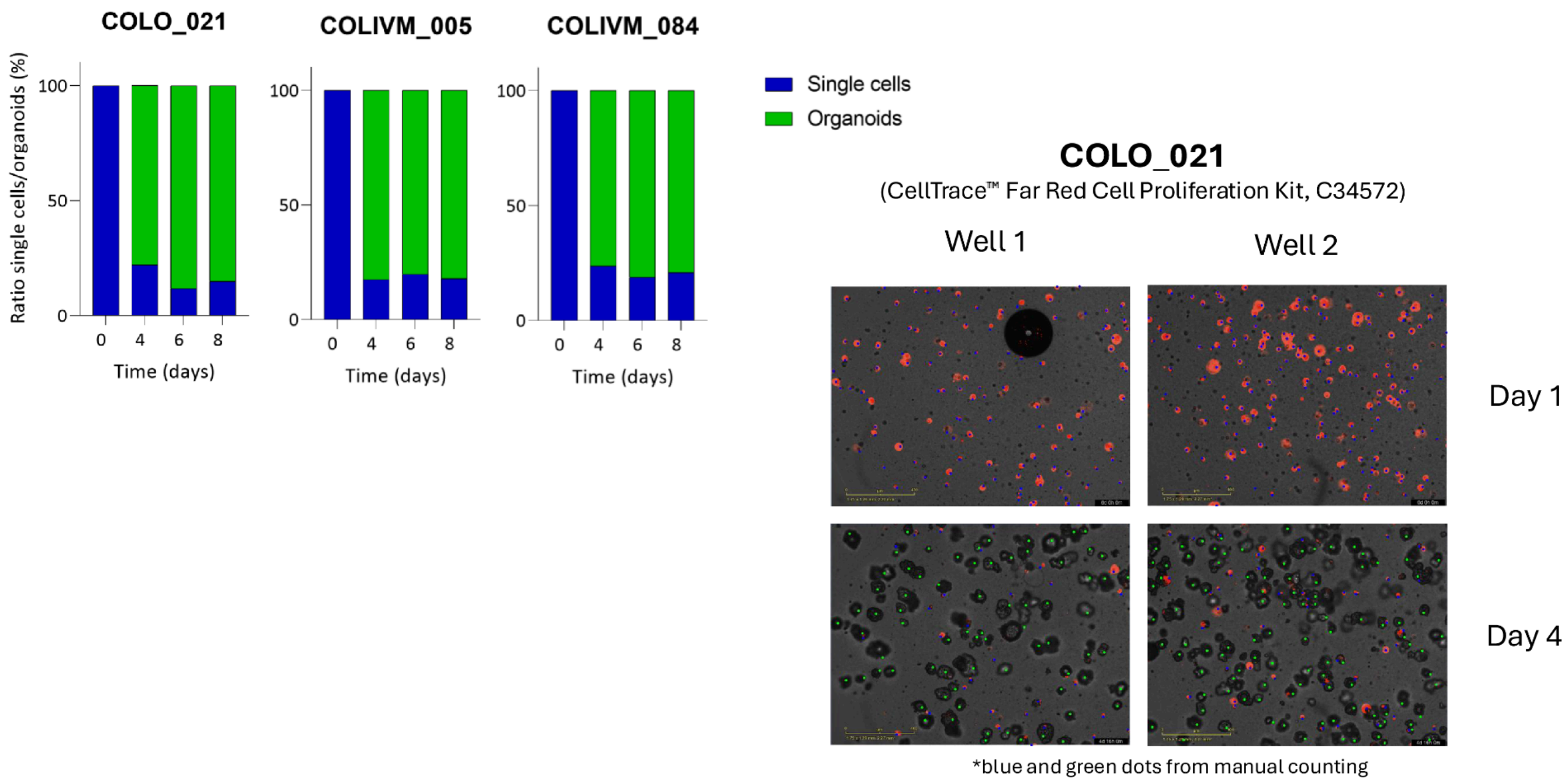

Figure: Assessment of Organoid Colony Formation Efficiency. *Top left: Proportion of cells forming cells at Days 0 (day of passage), 4, 6 and 8. Data are the average from 6 replicates. Bottom right: representative images for organoids at Day 1 and Day 4. Cells were stained with CellTrace to track proliferating organoids and formed organoids are marked with a green dot.*

CRISPR screens in organoids are technically challenging, nonetheless, our data meets established benchmarks for quality and reproducibility. This included strong replicate correlation and clear separation between essential and non-essential genes, comparable to benchmarks from large-scale cell line screens (e.g., the DepMap dataset). Specifically, our

organoid screens achieve an AUROC of 0.977 for essential gene classification, comparable to that observed in DepMap cell lines (AUROC = 0.92) (Fig.4c and e; Extended Data Fig. 14).

In further support of data quality, we recapitulate well-characterised genetic dependency–biomarker associations, examples include:

- KRAS dependency in KRAS-mutant models
- WRN dependency in MSI-high models
- MDM2 dependency in TP53 wild-type models
- EGFR dependency in KRAS wild-type contexts
- CCNE1 dependency in CCNE1-amplified models

These findings reinforce the biological relevance and robustness of our screens.

Guide log-fold changes were calculated by comparing to the plasmid readcounts, consistent with previous studies (PMID: 30103702, 30971826, 33478580, 36060092, 37028423), as opposed to comparing to a no Cas9 organoid control or including an early timepoint after infection. This was an experimental design trade-off between including an additional control for each organoid versus the additional statistical power achieved through screening additional organoids. To directly address the reviewers question, we have now repeated independent CRISPR screens in three organoid models with an early time point (5 days) to assess initial sgRNA representation and ensure uniform library delivery. Comparison of normalized counts shows that the distribution of sgRNAs at the early time point closely mirrors that observed in the plasmid, indicating consistent library coverage after transduction (**see Figure below**). In contrast, post-screen replicates exhibit a marked depletion of sgRNAs targeting essential genes and a broader distribution among non-essential and other genes, as expected following selective pressure (COLO_027 organoid shown as an example, same results in OESO_191 and OESO_308). These findings support the interpretation that the observed changes in guide abundance reflect true biological effects rather than initial library bias following transduction.

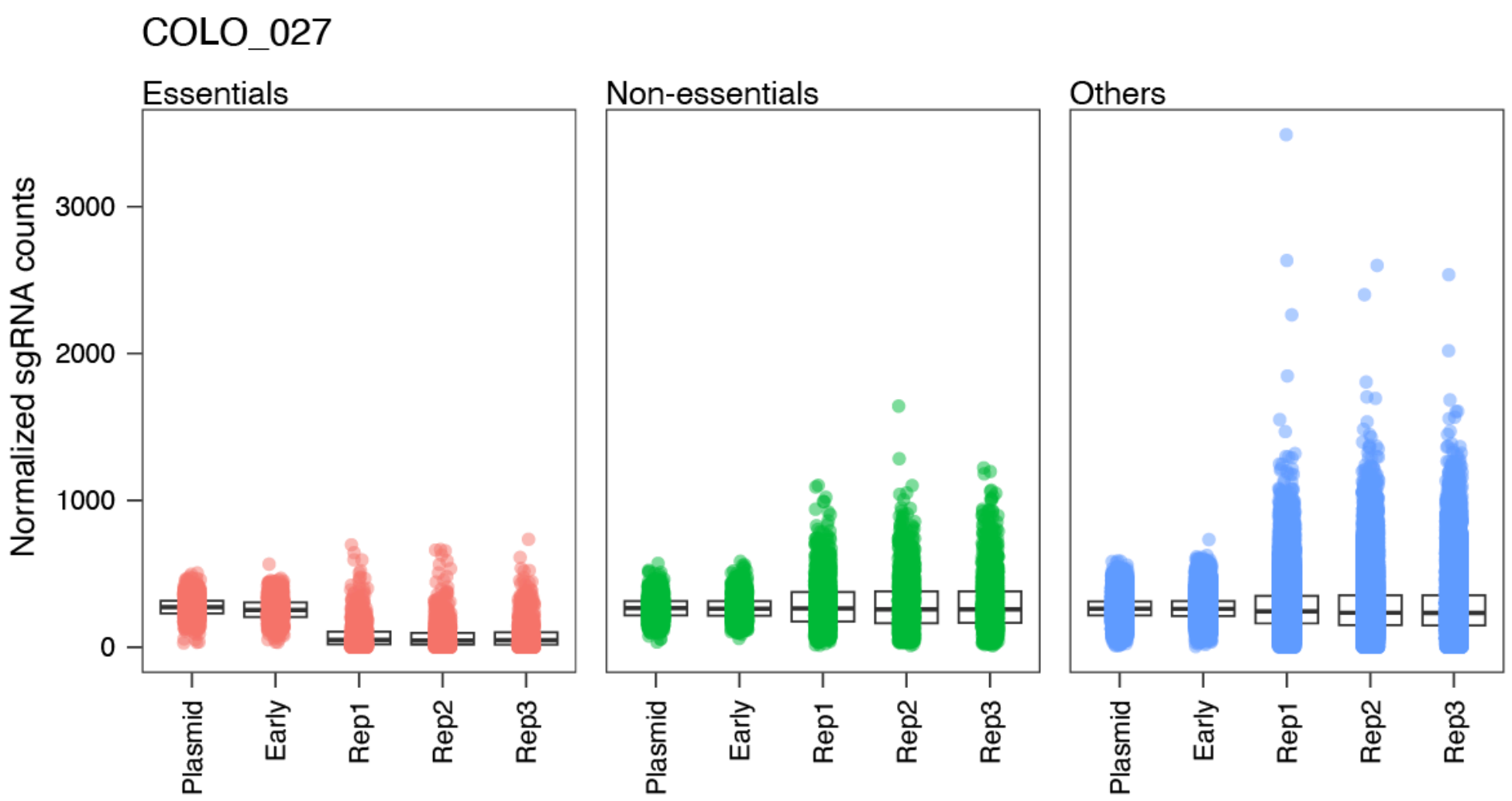

Figure: Equivalent Distribution of Guide Counts in Plasmid and Transduction Control. *Comparison of normalised sgRNA counts for library plasmid, 5 days post transduction (Early) and each experimental replicate at the end of the experiment. sgRNAs are grouped by whether they target a known essential or non-essential or other gene. These data are representative of 3 organoids.*

The Wellcome Sanger Institute is operated by Genome Research Limited, a charity registered in England with number 1021457 and a company registered in England with number 2742969, whose registered office is 215 Euston Road, London, NW1 2BE

In addition, when gene-level fold change values are calculated using either the plasmid or the early time point as a reference, the results correlate very strongly between the two conditions (**see Figure below**), further supporting that library delivery was unbiased and supporting the use of the plasmid read counts for calculating log-fold change values.

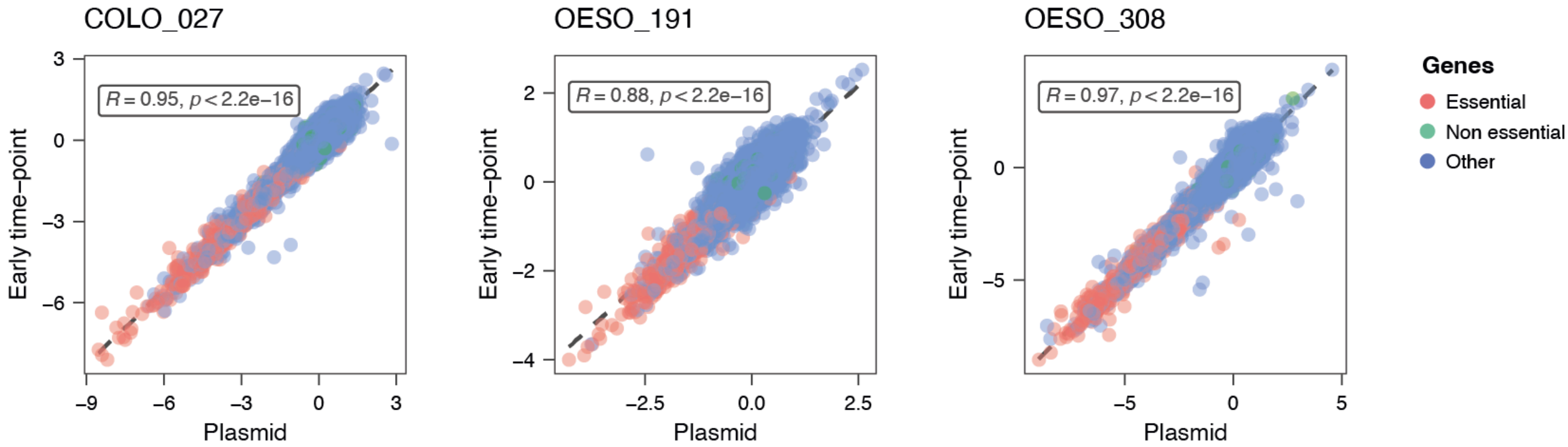

Figure: Gene level log-fold Change Values are Equivalent When Using Plasmid or Early Time Point Control. *Data are shown for 3 organoids.*

Collectively, we believe that these new results provide multiple strands of evidence that support the quality of our CRISPR screen framework and the unique value of the associated dependency datasets reported here.

4- Validation using in vivo experiments is completely lacking. Although the main focus of this article may be to show the fidelity of patient-derived organoids and applicability of CRISPR screening, the reviewer really thinks that some (but not all) sensitivity data should be validated using xenograft models of pertinent organoid lines.

The main objective of this manuscript is to present the organoid biobank and the associated datasets as a resource, showcasing their value through specific applications. As such, we believe in vivo validation is beyond the scope of this study. We now, however, acknowledge this limitation in the manuscript discussion and suggest that future studies incorporating xenograft studies could further validate the utility of our biobank and provide new biological insights

Minor comments:
1- In lines 201–202, the authors mention “Organoid derived from metastatic lesions were almost entirely derived from stage IV patients”, but this is confusing, as patients with distant metastasis automatically have a stage IV disease. What does the derivation of metastatic organoids from stage II and III patients in Extended Data Fig. 3b mean?

We thank the referee for pointing out this inconsistency. The referee is correct that organoids derived from metastatic lesions must originate from stage IV patients. After reviewing the clinical annotations with the contributing clinical sites, we identified a labeling error in the staging information for a small subset of samples. We have deleted Extended Data Fig. 3b panel and corrected the manuscript to ensure the terminology is accurate and consistent.

2- What are the definitions of the reasons of failure in Extended Data Fig. 1a? These should be stated in the legends or Methods. For example, what is the difference between slow culture growth, culture senescence and short-term culture? How do “normal tissue sample” and “no cancer drivers found” differ?

We have updated the manuscript to include more detailed explanations in Supplementary Table 1, providing clearer definitions of failure reasons. We will reference Supplementary Table 1 for further clarification of these terms in the legend of the Extended Data Fig.1. We acknowledge that the difference between culture senescence and short-term was minimal, so we have merged both categories. In addition, we have changed "normal tissue sample" to "unmatched normal sample" to better describe this category.

Referees #4 and #5 (Remarks to the Author):

This work reports the generation of 256 cancer organoid models, 123 of which are distinct from the HCMI models described in a companion paper. This paper represents a significant effort to establish a large biobank of organoid models for broad distribution within the scientific community. The study exemplifies the successful collaboration of multiple research teams necessary to develop such a valuable resource.

The techniques and approaches in the resource generation overall are appropriate. The inclusion of matched samples of multiple tumors/timepoints from the same individual will be extremely useful.

We thank the referees for highlighting the collaborative nature of our study and overall positive feedback.

Major comments:
1- There is an opportunity missed to determine whether the expense and low success rate of organoid modeling is needed, or whether traditional 2D cancer cell line models would show the same 1) success rate and 2) cancer dependencies. This group seems to be one of the only groups that are equipped to address this burning question, and unfortunately it was not addressed or even discussed at all. Based on this group's experience, would they recommend further investment in organoid generation, or in 2D cell line generation?

We appreciate the valuable insights and important questions raised. Our study was designed to establish a high-quality resource based on state-of-the-art organoid culture technology. Organoid technologies have substantially improved derivation efficiencies to expand the spectrum of tissues that can be propagated and studied *in vitro* (PMID: 31171691), which was a central focus of our effort.

Historically, success rates for establishing new cancer models have been low and variable, including for conventional 2D cell lines. For example, reported success rates were <10% for colorectal cancer cell lines derived directly from primary tissue (3/31 tumours; PMID: 17210723), 11–33% for head and neck squamous cell carcinoma cell lines (PMID: 2766286), 24% for lung adenocarcinoma cell lines (PMID: 31882684), and 9–38% across a wide range of tumour types (PMID: 29241554). These rates are similar to, or often lower than, those reported in our study. Moreover, success criteria are often inconsistently defined, and some studies used feeder layers, making direct comparisons of success rates difficult. We also note that derivation efficiencies are inherently context dependent—varying with

tissue type, histological subtype, clinical stage, tumour grade, genetic background, and culture conditions. Given the limited availability of patient tumour material, the historically lower success rates of 2D models, and budget available for this study, we did not consider a direct comparison of 2D versus 3D derivation success rates feasible. A dedicated, adequately powered study would be best suited to address this question.

A comparison of genetic dependencies between 2D and 3D cancer models presents several challenges due to multiple confounding factors, including differences in culture conditions and biases in the composition of available model cohorts. Such complications are not unique to 3D versus 2D comparisons: for example, CRISPR/Cas9 screens in the K562 cell line grown in human plasma-like medium (HPLM) versus RPMI revealed conditional dependencies shaped by media composition (PMID: 33651980), and variations in oxygen tension during culture have similarly been shown to influence CRISPR screen outcomes (PMID: 32259488). Consistent with this, the accompanying HCMI study reported that cancer cell states can vary across culture media Although some organoid cultures can be adapted to 2D growth formats to enable more direct comparisons, this process can impose selection pressures, leading to bottlenecking and potential loss of cellular heterogeneity. Additional validation studies will therefore be required to definitively attribute observed differences to culture-specific effects. We also note the accompanying study from the Broad Institute, which directly investigated dependencies across matched 2D and 3D culture systems.

Despite these complexities, large-scale dependency datasets such as those here, and the broader Cancer Dependency Map initiative, have proven invaluable community resources, with good concordance between datasets previously generated at the Broad and Sanger Institute (PMID: 33712601), and with many observations independently validated in follow-up studies—supporting the overall utility of building comprehensive dependency maps.

In summary, for these reasons, we believe that a focused, well-controlled comparison of 2D and 3D cultures should be pursued as a separate study. We continue to advocate investment in model generation, particularly for rare or underrepresented cancer types, while recognising the enduring value and complementarity of traditional 2D models.

2- Although the authors demonstrate that these models survive a single freeze-thaw cycle and meet QC metrics, it is crucial to assess whether genomic and phenotypic stability is maintained over multiple freeze-thaw cycles (meaning can users expand and store these models for long-term use or not). Establishing such stability is essential to ensuring the widespread usability, replicability and reliability of these models for researchers.

We recognise the importance of assessing the genomic stability of organoid models and have now addressed this through multiple lines of investigation. First, for 125 organoids, we have performed targeted gene sequencing (TGS) for 305 cancer driver genes both before and after the initial freeze–thaw QC step, with the two time-points separated by 4 passages. Overall, cancer driver mutations were highly retained in both samples (**see Figure below**), with a few exceptions.

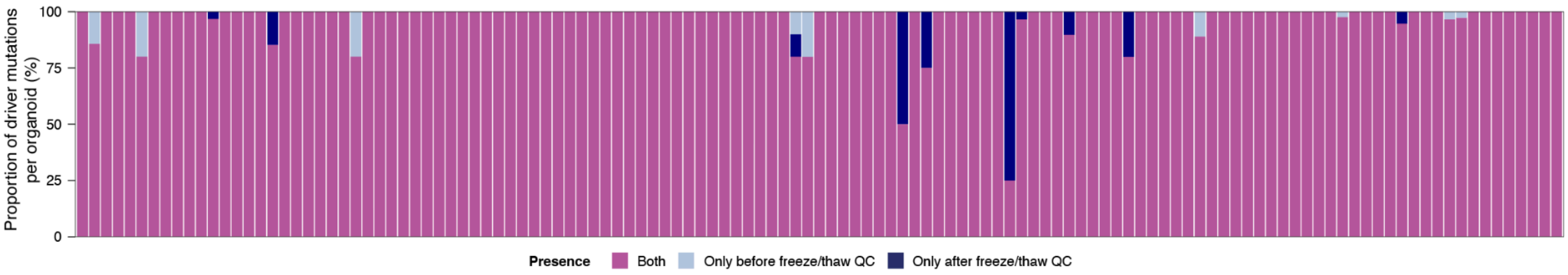

Figure: Cancer Driver Mutations are Retained in Culture. *Proportion of driver gene mutations shared between organoid cultures before and after freeze-thaw QC and 4 passages (both), versus exclusive to either samples.*

For six of these organoids, additional TGS data were also available following a second freeze–thaw cycle, with samples prepared at Sanger and independently by ATCC. In parallel, we conducted experiments involving multiple sequential freeze–thaw cycles in two of these organoid models, which were also cultured for up to 6 months (Extended Data Fig. 12). At each stage, we have performed both WGS and RNA-seq to assess the impact of repeated freeze–thaw events on genomic stability, comparing all available time-points (**see Figures below**). Across all 6 organoids, changes in the VAF of cancer driver mutations were minimal, and no new driver mutations emerged.

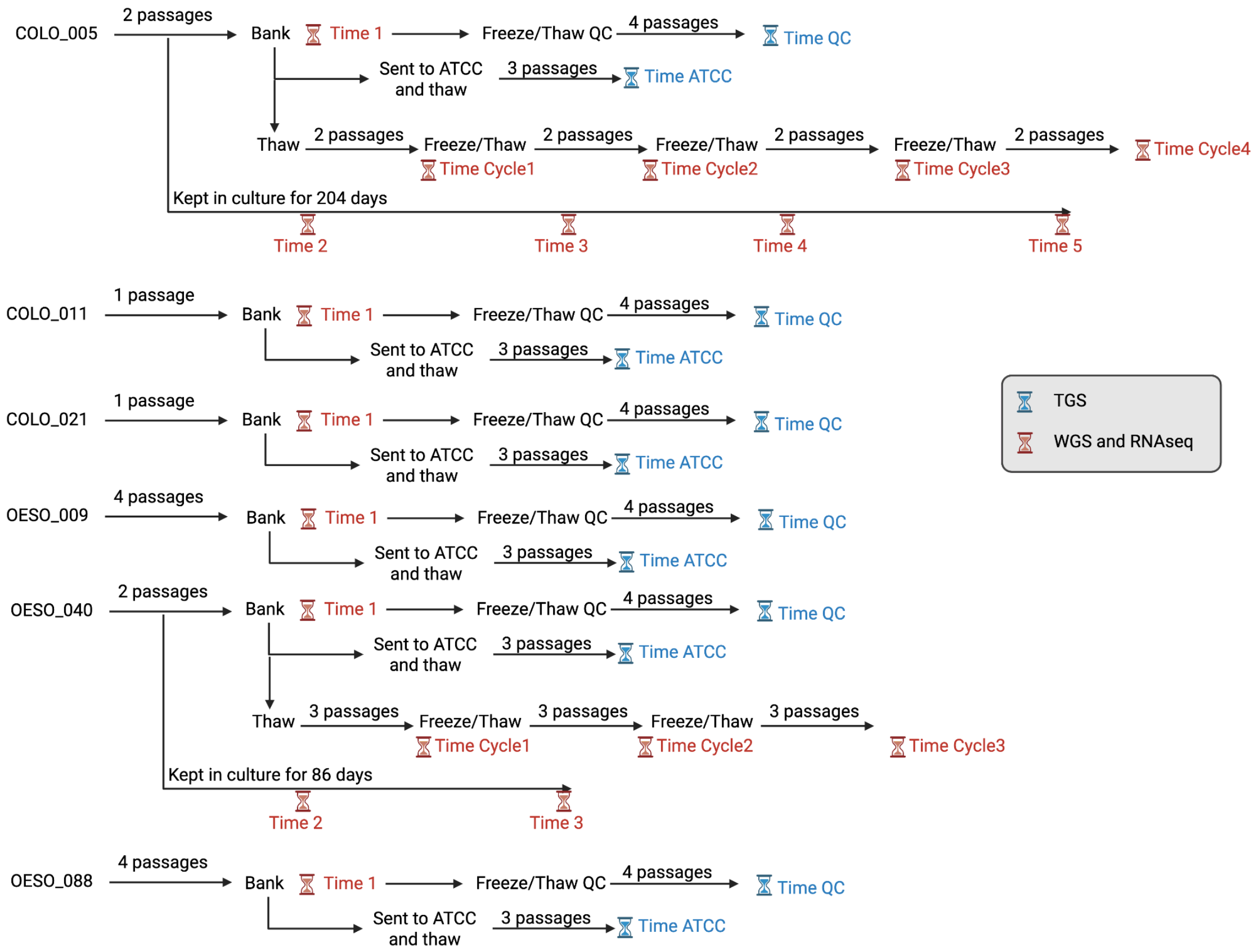

Figure: Sample Collection To Evaluate Organoid Stability in Culture. *Schematic showing prolonged culturing, freeze-thaw cycles and molecular analysis performed on organoid cultures to*

The Wellcome Sanger Institute is operated by Genome Research Limited, a charity registered in England with number 1021457 and a company registered in England with number 2742969, whose registered office is 215 Euston Road, London, NW1 2BE

assess their stability in culture. Samples were prepared at Sanger Institute and independently by ATCC.

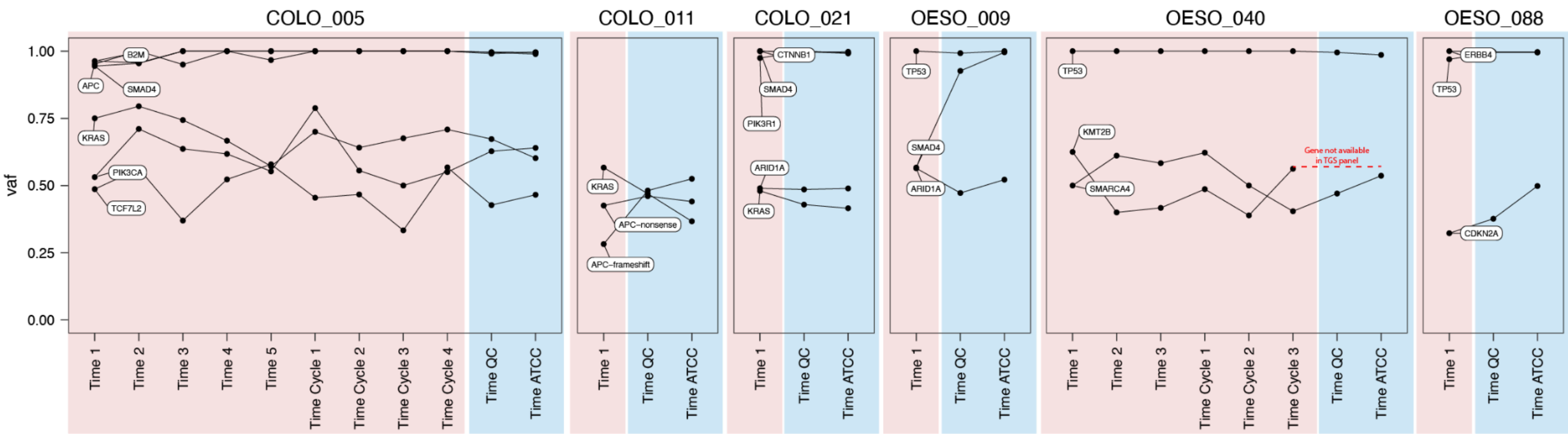

Figure: Organoid Cultures are Genetically Stable In Culture. *Variant allele frequencies of cancer driver genes in 6 organoids over prolonged culture and multiple rounds of freeze-thaw QC. Organoid cultures underwent TGS (blue) or WGS (pink background). No new cancer driver gene mutations emerged.*

In the two models subjected to extended culture and multiple freeze–thaw cycles, the Jaccard Index—measuring the proportion of shared SNPs and Indels—remained high across all time points (**see Figures below**). However, long-term culture introduced greater genetic drift than repeated freeze–thaw cycles. RNA-seq profiles were also stable, supporting the same conclusion that genetic drift is primarily driven by extended culture rather than freeze–thaw events.

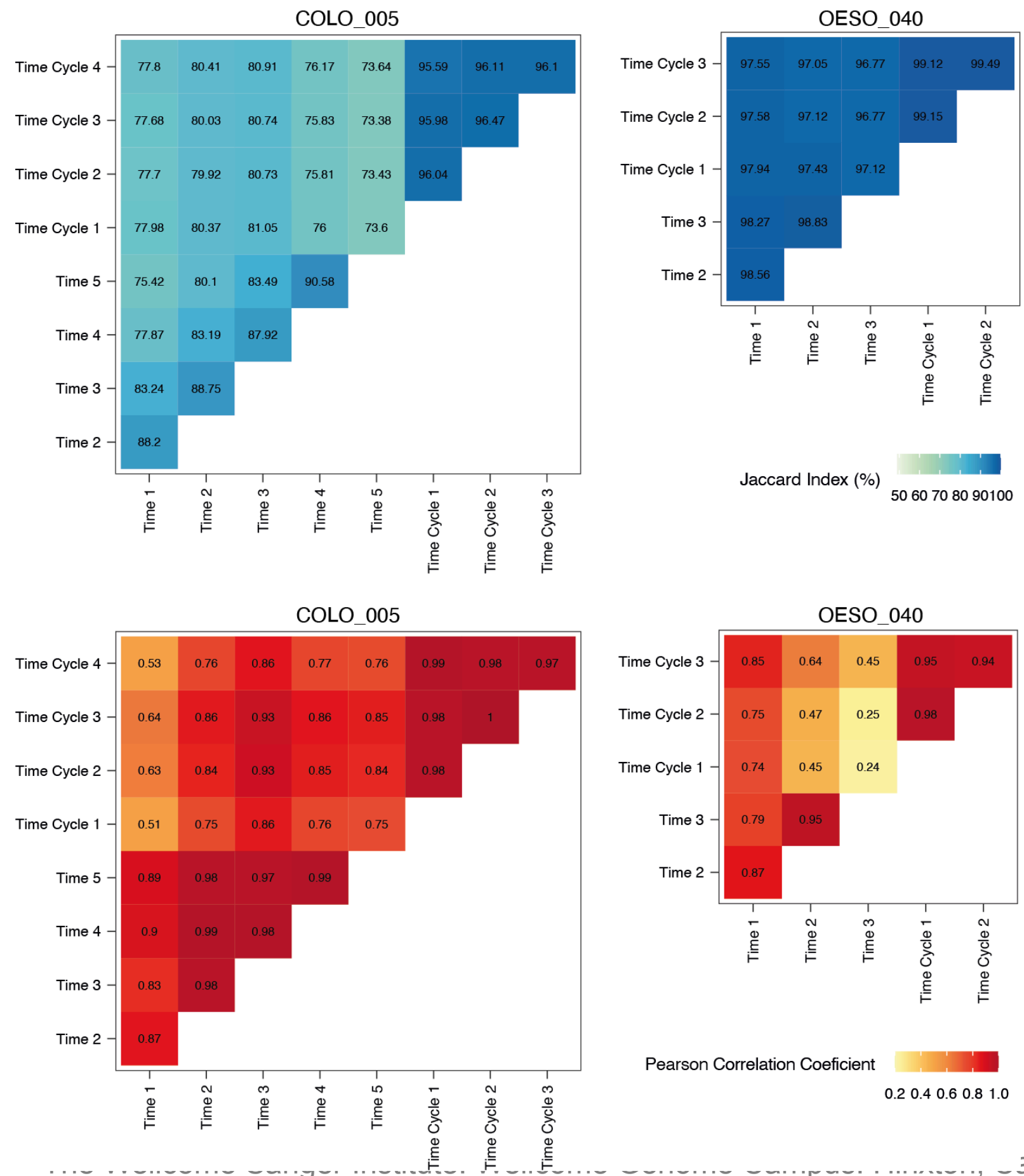

Figure: Conservation of Mutations and Gene Expression Over Prolonged Culture. *Upper panels: Jaccard index calculated from pairwise comparisons of SNPs and Indel's across samples acquired from multiple timepoints and freeze-thaw cycles. Lower panels: correlation of RNA sequencing gene expression profiles.*

Additionally, we have previously demonstrated the genomic and phenotypic stability of organoids during prolonged culture periods (PMID: 30061675, PMID: 35368031).

Lastly, several published studies have used organoids from this biobank, including multiple independent labs, demonstrating that these are robust models suitable for a wide range of applications by other groups, including scRNA-seq, base editing, drug screening, and CRISPR gene editing experiments (PMID: 38065081, 38587317, 39424923, 29853607, 37565053, 38744814, 38232180).

Collectively, these results demonstrate the robustness and reliability of our models.

3- The extremely unique data that this resource is providing is the CRISPR dataset, but it is underexplored. It appears raw data is shared, but will the CRISPR datasets be added to DepMap or other CRISPR resources?

Yes, the organoid CRISPR data will be made available through the DepMap Miner website (https://dataminer.depmap.sanger.ac.uk/) prior to publication, and longer term we support incorporation into the DepMap portal hosted by the Broad Institute. We provide detailed information about these data in the manuscript.

4- A more systematic analysis of the organoid-specific vulnerabilities should be performed that goes beyond gene-set enrichment analysis. Are there different dependencies identified in this approach compared to cell lines, and are these different due to the new genetic architecture of these new models, or due to culture conditions? The same cell line or organoid models should be screened in 2D vs 3D to understand to what effect the culture conditions rather than the model properties influence the cancer dependency landscape.

We agree that a deeper analysis of organoid-specific dependencies is of interest. We have focused our analysis on core fitness genes because of their critical role and this is less likely to be biased by the set of models screened when comparing cell lines versus organoids. While the majority of core fitness genes were conserved, we make the new finding that organoids have an increased requirement for steroid biosynthesis and cholesterol related genes (Extended Data Fig.15a), a finding independently verified in the accompanying paper from the Broad Institute. Furthermore, we demonstrate that these genes are enriched for genes under high genetic constraint based on studies of human populations, providing orthogonal support for our experimentally-derived identification of core fitness genes (**Fig.4e**).

With respect to screening the same organoid in 2D versus 3D, we refer the reviewer to our previous response to their major comment 1, where we argue that, given the importance of this topic, a well powered dedicated study would be more appropriate. To support future

investigations on this topic, we have made detailed culture protocols publicly available, and by making the models available as a community resource, we are enabling other groups to address these important questions. We have also explicitly discussed in the revised manuscript the potential impact of *in vitro* culture conditions on phenotypes and dependency landscapes, and highlight this as an important avenue for future research.

Minor comments:

1- A reviewer note on p. 5 indicates the models will be distributed commercially – please update vendor/information prior to publication. Overall, this paper is an incredible resource of models. However, the impact of this work will be limited by the commercial distribution of these models which will be cost prohibitive for many investigators.

Up-to-date vendor information is included in the Supplementary Table 2 of the manuscript.

We feel it would be useful to provide context for our choice of distribution approach. We investigated multiple strategies for biobank distribution, including commercial repositories, non-profit biobanks (e.g. CancerTools.org), and distribution directly by Sanger. The accessioning, maintenance, and long-term distribution of the biobank requires sustained financial investment and infrastructure that are not feasible at the Sanger Institute, or most other organisations. We have therefore deposited 125 organoid models with the non-profit repository ATCC. Currently, 48 Sanger-derived models are available for purchase (https://www.atcc.org/cell-products/collections-and-projects/human-cancer-models-initiative/hcmi-unmanufactured-models), with additional models scheduled for expansion guided by the community to prioritise model distribution (https://www.atcc.org/cell-products/collections-and-projects/human-cancer-models-initiative/hcmi-unmanufactured-models). In addition, we have licensed the Sanger organoid biobank to the Life Sciences business of Merck KGaA, with distribution via their platform expected to begin in Q1 2026. Both ATCC and Merck bring the financial resources, infrastructure, global distribution networks, and proven track record necessary to ensure consistent quality, wide accessibility, and the long-term sustainability of the biobank. We believe this dual-repository strategy provides a long-term, sustainable solution to maximise global access to the Sanger organoid biobank. The commercial success of ATCC and Merck to distribute organoids will depend on making the model costs accessible for investigators, mitigating the risk that these costs will be prohibitive for end users.

2- It would be valuable to determine whether clinical factors were significantly associated with the inability to establish organoid models, such as patient age, sex, or tumor stage.

We would have liked to compare clinical data between tumors that failed or succeeded in organoid derivation; however, because clinical data were collected retrospectively only after successful derivation, this analysis was not feasible. Instead, we evaluated clinical features in relation to derivation metrics among successfully established organoids. The only statistically significant association observed was that the size of the initial tumor sample correlated with tumor stage (Extended Data Fig. 1h)

3- P17 line 556-558 – the increased dependency of KRAS wt organoids on EGFR, PTPN11, and ERBB2 in Fig 5a is not readily apparent in the heatmap visualization and this statement should be strengthened with a statistical approach and an alternative data visualization.

We have substantially revised Fig. 5a and provide a statistical analysis to strengthen the dependencies observed in KRAS wild-type organoids.

4- In the methods, please cite specific protocols at protocols.io that were used. Many investigators may want to replicate the approaches used here, but the information seems to be missing from the Methods.

The relevant protocols on protocols.io are now properly cited.

5- The lollipop plots in Extended Data Fig 6 are confusing. Please number or color the sample IDs above and put the numbering on y axis to make meaning more readily apparent.

We have improved clarity by adding numbered sample IDs and labeling the y-axis appropriately.

6- Extended Data Fig 8 needs lettering to break up the figure and provide clarity with the link to the figure legend descriptions.

We have updated Extended Data Fig. 8 as suggested.

— end of reviewers’ comments and author response —-

The Wellcome Sanger Institute is operated by Genome Research Limited, a charity registered in England with number 1021457 and a company registered in England with number 2742969, whose registered office is 215 Euston Road, London, NW1 2BE
